# Targeting double-strand break indel byproducts with secondary guide RNAs improves Cas9 HDR-mediated genome editing efficiencies

**DOI:** 10.1038/s41467-022-29989-9

**Published:** 2022-05-09

**Authors:** Zsolt Bodai, Alena L. Bishop, Valentino M. Gantz, Alexis C. Komor

**Affiliations:** 1 Department of Chemistry and Biochemistry, University of California San Diego, La Jolla, CA 92093 USA; 2 Division of Biological Sciences, Section of Cell and Developmental Biology, University of California San Diego, La Jolla, CA 92093 USA

**Keywords:** Genetic engineering, Nucleic acids, CRISPR-Cas9 genome editing

## Abstract

Programmable double-strand DNA breaks (DSBs) can be harnessed for precision genome editing through manipulation of the homology-directed repair (HDR) pathway. However, end-joining repair pathways often outcompete HDR and introduce insertions and deletions of bases (indels) at the DSB site, decreasing precision outcomes. It has been shown that indel sequences for a given DSB site are reproducible and can even be predicted. Here, we report a general strategy (the “double tap” method) to improve HDR-mediated precision genome editing efficiencies that takes advantage of the reproducible nature of indel sequences. The method simply involves the use of multiple gRNAs: a primary gRNA that targets the wild-type genomic sequence, and one or more secondary gRNAs that target the most common indel sequence(s), which in effect provides a “second chance” at HDR-mediated editing. This proof-of-principle study presents the double tap method as a simple yet effective option for enhancing precision editing in mammalian cells.

## Abstract (web summary)

Programmable double-strand DNA breaks (DSBs) can be harnessed for precision genome editing through manipulation of the homology-directed repair (HDR) pathway. Here the authors report the development of the double tap - double tap implements secondary gRNAs which target Cas9 to common indel sequences and provides a second chance at HDR.

## Introduction

**Fig. 1:**
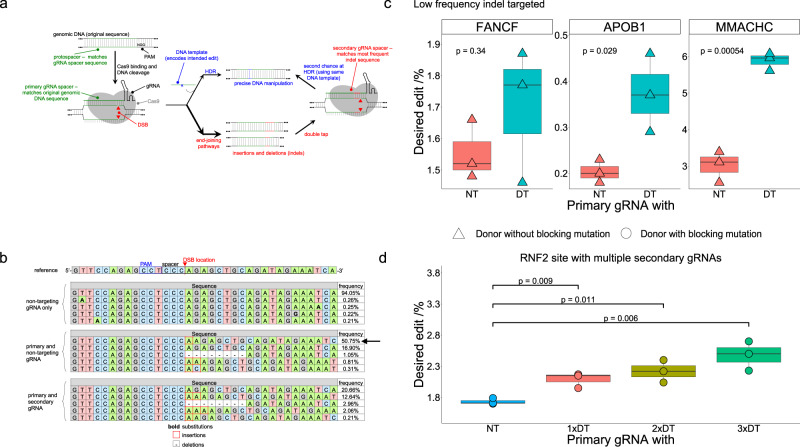
Schematic and initial results of the double tap method. **a** Schematic overview of the double tap method. Cas9 introduces a DSB at a locus of interest using the primary guide RNA. HDR processes a subset of the DSBs into the desired outcome using a donor template (blue sequence). Concurrently, indels are introduced at the DSB site via end-joining pathways (red sequences). These undesired indel sequences are subsequently targeted with secondary gRNAs to improve overall yields of the desired outcome through a second DSB introduction and sequential HDR repair. **b** Indel sequences and their corresponding introduction efficiencies at the *MMACHC* site after transfecting HEK293T cells with Cas9 and a non-targeting gRNA (top), the primary gRNA plus a non-targeting gRNA (middle), or the primary gRNA plus a secondary gRNA targeted to the indel sequence indicated with the black arrow (bottom). **c** HDR-mediated genome editing efficiencies at the *FANCF* (in which a low-frequency indel was targeted), *APOB1*, and *MMACHC* sites when HEK293T cells are transfected with an ssODN and plasmids encoding Cas9, the primary gRNA, and either a non-targeting gRNA (NT, left) or secondary gRNA(s) (DT for “double tap”, right; two secondary gRNAs were used with the *FANCF* primary gRNA, and one secondary gRNA was used at the other two sites). Plotted are the percent of total DNA sequencing reads with the desired modification introduced (perfect HDR products without indels). **d** HDR-mediated genome editing efficiencies at the *RNF2* locus when HEK293T cells are transfected with an ssODN and plasmids encoding Cas9, the primary gRNA, and either a non-targeting gRNA (NT, far left) or one (1 x DT), two (2 x DT), or three (3 x DT) secondary gRNAs. Values on whisker plots represent the lowest observation, lower quartile, median, upper quartile, and the highest observation of three independent replicates. Data were analyzed with univariate statistics (one-way ANOVA [one-sided]), and *p* values are labeled on the graphs.

Clustered regularly interspaced short palindromic repeat (CRISPR) systems have revolutionized the genome editing field over the past decade. The most widely used type II CRISPR system consists of two main elements: an engineered chimeric single guide RNA (gRNA) and the DNA endonuclease protein Cas9 (CRISPR-associated protein 9)^1^. The gRNA is easily programmed as it facilitates Cas9 to bind to a target site of interest via sequence complementarity with the target DNA sequence (called the protospacer), which must be directly next to a protospacer adjacent motif (PAM). In the *Streptococcus pyogenes* (Sp) system (used in this work), the protospacer is 20 bases long, and the PAM sequence is NGG (Fig. 1). After successful DNA binding, the SpCas9 protein cleaves the DNA backbone to introduce a double-strand break (DSB) at the desired genomic locus.

The DSB can be repaired via two main pathways: either re-ligation of the broken ends by end-joining pathways, or templated repair via homology-directed repair (HDR). Re-ligation is mainly mediated by non-homologous end joining (NHEJ) or microhomology-mediated end joining (MMEJ), which result in insertion and deletion (indel) sequences at the site of the DSB under genome editing conditions. In contrast, HDR uses a sister chromatid as a template to repair the DSB in a precise manner^2^. The endogenous HDR pathway can be manipulated to precisely insert DNA sequences by providing the cell with an artificial donor template harboring modifications of interest. Under typical genome editing conditions, both pathways are active and compete to process the DSB intermediate, resulting in mixtures of precision HDR-mediated products as well as end-joining-mediated indel products.

Since the initial demonstration of HDR-mediated genome editing using Cas9 in human cells^3–6^, there have been numerous studies that have improved the ratio of HDR-mediated to end-joining-mediated genome editing products^7,8^. Specifically, a variety of strategies involving donor template modifications have improved HDR-mediated editing efficiencies, including: (1) phosphorothioate end modification of the template, potentially due to the longer residence time within the cells of the template when modified^9^; (2) optimization of homology arm length of the donor template when using a single-stranded oligodeoxynucleotide (ssODN) template, both with symmetric^10^ and asymmetric homology arms^11^; (3) fusion of the ssODN donor template to the Cas9 protein, potentially due to enhanced nuclear import of the donor template when covalently attached to Cas9^12,13^; and (4) installation of silent mutations in the PAM or PAM-proximal regions of the protospacer, which prevents the Cas9:gRNA complex from binding and re-cutting the genomic DNA following a successful HDR event^14^. Additionally, as HDR is primarily limited to the synthesis (S) and gap 2 (G2) phases of the cell cycle, methods to manipulate cell cycle phases have been shown to impact HDR outcomes^15,16^. In addition, small molecules have been used to inhibit end-joining pathways (by targeting key end-joining repair proteins such as DNA Ligase IV^17^, DNA-PKcs^18^, and 53BP1^19^) to increase relative HDR to end-joining ratios as well. Finally, fusion of Cas9 to different DNA repair proteins, such as CtIP^20^ and Rad51^21^, have also been shown to enhance HDR-mediated editing efficiencies.

Motivated by this need to enhance the efficiency of precision genome editing outcomes, other CRISPR-based genome editing technologies have emerged recently, such as base editing^22,23^ and prime editing^24^. Although these technologies enable genome editing with greatly enhanced precision, they have certain restrictions and limitations that are not an issue with traditional HDR-based methods. For example, base editors can only install transition mutations and have strict protospacer design requirements that prevent certain bases from being viable base editor targets. Furthermore, if multiple target bases are present within the “base editing window” for a given protospacer, they may all become edited at once, reducing the precision of base editing (referred to as bystander editing). Although prime editing can overcome these issues, editing efficiency is often low without use of additional “nicking gRNAs”, which has the undesired side effect of increasing indel formation at the target site. Additionally, the sheer possible number of prime editing gRNA (pegRNA)-nicking gRNA combinations for a given modification of interest makes finding the optimal construct cumbersome. Finally, neither base editing nor prime editing can facilitate the insertion of large DNA sequences such as gene knock-ins^25–27^, and certain specialized applications, such as gene drive technologies^28^, explicitly require HDR and therefore cannot be performed with base editing or prime editing.

It has recently been acknowledged that indel sequences arising from a given DSB are generally reproducible and depend on the sequence surrounding the DSB. Sites with low microhomology (<4-nt of homology) are thought to be mainly processed by NHEJ, which often generates one base pair insertions^29,30^. In contrast, sites with high microhomology (5- to 25-nt of microhomology) are efficiently processed by MMEJ, which results in well-defined deletions of the bases between the microhomology sites. Inspired by these observations, researchers have developed algorithms to predict indel products. One such software, “Microhomology-Predictor,” can predict MMEJ deletion outcomes, and was developed to help researchers identify optimal cut sites that avoid MMEJ-mediated deletions that do not result in frame-shift mutations^31^. Another, inDelphi, was generated using machine learning based off a dataset of 2,000 gRNA-DNA target site pairs and corresponding indel sequences and can predict indel sequence outcomes (including both NHEJ-mediated insertions and deletions, as well as MMEJ-mediated deletions) in different cell lines^32^. In addition, inDelphi can predict the distribution frequency of indel products. While for many sites, indel products are heterogenous, it is estimated that 5–11% of gRNAs produce a single repair outcome that represents more than 50% of repair products, and 27–47% of gRNAs produce a single repair outcome that represents more than 30% of repair products. We therefore envisioned a method that takes advantage of the reproducible and predictable nature of these high frequency indel sequences to improve HDR-mediated genome editing.

This study describes the development of the “double tap” method, which uses additional gRNAs (called secondary gRNAs) to target high frequency indel products created by end joining pathways during an attempted HDR event (Fig. 1a). Normally, these indel products cannot be processed by Cas9 as they do not match the original gRNA sequence. However, when complemented with secondary gRNAs, these sequences can be re-targeted, providing a second opportunity for the DSB to be processed by HDR using the same donor template. We reasoned that these secondary gRNAs could decrease unwanted indel products and increase the desired precision genome editing outcome. Here, we test the double tap method in multiple human cell lines at 15 different genomic loci. We design and test secondary gRNAs targeted to indel sequences with a wide range of frequencies and observe larger improvements in HDR-mediated genome editing efficiencies when targeting higher frequency indel sequences, with no increases in indel rates (in many instances, we in fact observe decreases in indel rates). We demonstrate the ability of the double tap method to improve HDR-mediated genome editing efficiencies for the installation of point mutations, small insertions, and deletions with ssODNs, as well as for gene knock-in using dsDNA donor templates. This method can be easily integrated into any routine HDR experiment to boost precision editing efficiencies by characterizing the sequences of the most common indel products and incorporating secondary gRNAs to target these sequences.

## Results

### Initial testing of the double tap method in HEK293T cells for introducing small edits

We first selected four well-characterized genomic loci to test our hypothesis that targeting reproducible indel sequences with secondary gRNAs could boost HDR-mediated genome editing efficiencies. Specifically, we chose previously validated protospacers that target loci within the *APOB*, *MMACHC*, *RNF2*, and *FANCF* genes (hereafter referred to as the *APOB1*, *MMACHC*, *RNF2*, and *FANCF* loci or sites, respectively)^22,32^. To characterize the most common indel sequences introduced using these primary gRNAs, we transfected human embryonic kidney (HEK293T) cells with plasmids encoding Cas9 and primary gRNA. After 72 h, cells were lysed, genomic DNA (gDNA) was extracted, and loci of interest were amplified, sequenced using next-generation sequencing (NGS), and analyzed with CRISPResso2 to identify recurrent indels. The experimentally determined and predicted indel sequences (using inDelphi) are shown in Fig. 1b (for the *MMACHC* locus) and Supplementary Fig. 1. We will refer to the indel introduction efficiencies in these non-double tap experiments as “initial indel rates” from now on. Based on these indel data we designed one secondary gRNA each for the *APOB1* and *MMACHC* sites, two for the *FANCF* site, and three for the *RNF2* site. We chose to target these particular indel sequences as they were reproducible (occurred in all replicates and the inDelphi analysis) and represented a large range of initial indel rates (from 3 to 50%), allowing us to investigate the relationship between initial indel rate(s) of the targeted indel(s) and enhancement of editing efficiency after implementing the double tap method.

**Fig. 2:**
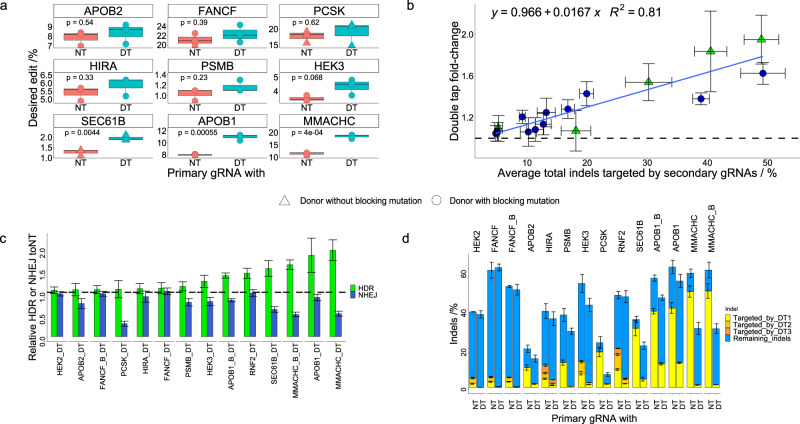
Improvements in HDR-mediated genome editing with ssODNs using the double tap method. **a** Shown are the percent of DNA sequencing reads with the desired modification introduced (perfect HDR products without indels) for cells treated with primary gRNA and a non-targeting gRNA (NT, left), or primary gRNA and secondary gRNA(s) (DT, right; three secondary gRNAs were used at the *HIRA* and *RNF2* sites, two secondary gRNAs were used at the *HEK2*, *HEK3* and *FANCF* sites, and one secondary gRNA was used at the *APOB1, APOB2, PSMB, PCSK, SEC61B* and *MMACHC* sites). Values on the whisker plots represent the lowest observation, lower quartile, median, upper quartile and the highest observation of three independent replicates. Data were analyzed with univariate statistics (one-way ANOVA [one-sided]) and *p* values are labeled on the graphs. **b** Average fold-change values plotted against the average of the total initial rates of the indels targeted by secondary gRNAs for all of the genomic loci tested in Figs. 1 and 2. Error bars represent the propagation of uncertainty of the SD for *n* = 3 biological replicates. **c** Shown are the relative changes in HDR (green) and NHEJ (blue) frequencies relative to the primary and non-targeting gRNA samples. Values and error bars represent the mean and propagation of uncertainty of the SD for *n* = 3 biological replicates. **d** Shown are total indel rates of all samples, with the specific indels targeted by secondary gRNAs shown in yellow, orange, and red (depending on how many secondary gRNAs were used for a particular site, there may only be yellow or yellow and orange bars). Blue represents indels not targeted by secondary gRNAs. Values and error bars represent the mean of the number of sequencing reads with indel sequences divided by the total number of sequencing reads ± SD for *n* = 3 biological replicates. In (**c**), (**d**), when the ssODN encoded a blocking mutation, the site is labeled with an “_B”.

We next designed ssODN templates to install either a point mutation (for the *RNF2* and *MMACHC* sites) or a small insertion (for the *FANCF* and *APOB1* sites) so that editing efficiencies could be monitored. Unless explicitly noted otherwise, all our ssODNs were designed symmetrically, with 50 or 70-nt homology arms (listed in Supplementary Data 1). We transfected HEK293T cells with ssODN and plasmids encoding Cas9, primary gRNA, and either non-targeting gRNA (to keep the total amount of gRNA plasmid constant when comparing to the double tap experiments) or secondary gRNA(s). After 72 h, cells were lysed and analyzed via NGS and CRISPResso2 to determine HDR and indel introduction efficiencies. We observed increases in absolute HDR-mediated genome editing efficiencies in all cases, with the relative size of the increase roughly correlated to the initial rates of the indel sequences that were targeted with the secondary gRNAs (we later expand our dataset and further analyze this relationship, Fig. 2b). Specifically, when using secondary gRNAs targeted to indels with high (40.5 ± 2.7% for *APOB1* and 50 ± 2.9% for *MMACHC*, see Methods for statistical analysis details) initial rates, the average HDR-mediated editing efficiency improved 1.8 ± 0.4 -fold for *APOB1*, and 2.0 ± 0.2 -fold for *MMACHC* (see Methods for statistical analysis details; data shown in Fig. 1c). When targeting indel products with moderate (9.2 ± 0.5% for the *RNF2* site) initial rates, the average overall HDR-mediated genome editing efficiency improved 1.2 ± 0.1-fold (Fig. 1d). As the *RNF2* site had two additional indel products with high frequencies, we additionally tested the impact of using two and three secondary gRNAs. When using two secondary gRNAs whose corresponding indels collectively had initial rates of 17.0 ± 1.1%, the double tap method boosted the average HDR-mediated editing efficiency by 1.3 ± 0.1-fold (Fig. 1d). Using three secondary gRNAs that collectively corresponded to initial indel rates of 19.9 ± 1.2%, the average HDR-mediated genome editing efficiency improved 1.4 ± 0.1-fold (Fig. 1d). Finally, when targeting indel products with low (5.3 ± 0.6% for the *FANCF* site) initial rates, we observed only a 1.1 ± 0.1-fold improvement (Fig. 1c; note two secondary gRNAs were used in this case to target two indels whose initial indel rates summed to 5.3 ± 0.6%). These data show that the double tap method can improve precision genome editing efficiencies. Additionally, these results suggest that the use of secondary gRNAs targeted to indel sequences with higher frequencies leads to larger improvements in HDR-mediated genome editing efficiencies than secondary gRNAs targeted to indel sequences with lower frequencies.

### Characterization of the double tap method

For further characterization and validation, we tested the double tap method at seven additional protospacers (within the *LOC110120638*, *LINC01509*, *HIRA*, *PSMB2*, *PCSK9*, *APOB*, and *SEC61B* genes, hereafter referred to as the *HEK2, HEK3, HIRA, PSMB, PCSK, APOB2*, and *SEC61B* loci or sites, respectively), using HDR to install point mutations, small deletions, and small insertions. Again, the double tap method increased HDR-mediated genome editing efficiencies at all tested sites, with larger fold-change values when using secondary gRNAs targeted to indel sequences with larger initial rates (Fig. 2a–c and Supplementary Fig. 2). We graphed fold-change values as a function of the collective initial indel rates targeted by the secondary gRNAs (Fig. 2b) to better visualize the correlation between these two factors. With this larger dataset, we confirmed our previous observation that larger increases in HDR efficiencies occur when targeting indels with larger initial rates. In fact, these data can be fit with a linear regression model (fold-change = 0.966 + 0.0167*[initial rate of indel(s) targeted with secondary gRNA(s)], r^2^ = 0.81 Fig. 2b), allowing for the approximation of fold-change values in future experiments (see “Disease-relevant sites” section for examples).

Furthermore, we also observed decreases in the total absolute indel rates when using the double tap method in ten out of eleven cases (Fig. 2c, d). In all cases, introduction rates of the specific indels targeted with secondary gRNAs decreased (Fig. 2d). At the same time, certain indel sequences that were present in the primary gRNA-only experiments at very low (generally <1%) frequencies increased in the double tap samples. In general, sites with lower initial indel rates targeted by secondary gRNAs showed smaller decreases in total indel rates (for example, at the *APOB2* site, an indel with an initial rate of 10.2 ± 1.3% was targeted with a secondary gRNA, and overall indel rates decreased by 25.4 ± 13.4%). Conversely, sites with higher initial indel rates targeted by secondary gRNAs showed larger decreases in total indel rates (for example, at the *MMACHC* site, a 1-bp insertion with an initial rate of 49.2 ± 3.7% was targeted with a secondary gRNA, and overall indel rates decreased by 48 ± 7%). However, these percent decreases in overall indel rates were not as well-correlated with initial indel rates as the fold-changes in HDR efficiencies. For example, at the *ABOB1* site, a 1-bp insertion with an initial rate of 40.5 ± 2.7% was targeted with a secondary gRNA, and the total indel rate decreased only by 12 ± 8% (while the HDR efficiency was improved 1.8 ± 0.4-fold). This relatively small decrease in the total indel rate is partially because the targeted indel was still present with a rate of 12.6 ± 0.4% in the double tap sample (in all other cases, the rates of the targeted indel(s) decreased to below 5%). Additionally, a 2-bp insertion present in the primary gRNA-only experiment at a rate of 0.14 ± 0.02% increased to 6.8 ± 1.1% in the double tap sample. The incomplete elimination of the 1-bp insertion secondary gRNA target in combination with the generation of this new indel product caused the overall indel rate to decrease only slightly. On the other hand, at the *PCSK* site, a 1-bp insertion indel with an initial rate of 18.1 ± 2.5% was targeted with a secondary gRNA, and the total indel rate decreased by 71 ± 17% (while the HDR efficiency improved only 1.1 ± 0.2-fold). However, we will note that while the double tap method does seem to decrease rates of small indels, the frequency of large on-target deletions may be changing^33,34^. We did not observe any large deletions within the sequenced amplicon, but deletions that occur outside of the PCR primer binding sequences would not be detected, and may account for the apparent decrease in small indel efficiencies at certain sites that were not accompanied by a significant increase in HDR rates. Nevertheless, the relative HDR to NHEJ ratios for all sites tested was either within error of the non-double tap samples (at two out of eleven sites) or improved up to 3.8 ± 0.6-fold (Supplementary Fig. 3). Overall, these data show that the double tap method not only improves HDR efficiencies but may also decrease overall indel rates.

### Additive effects combining double tap with other methods to improve precision editing outcomes

The use of blocking mutations at the PAM or the PAM-proximal region of the protospacer has been shown to improve HDR-mediated genome editing yields^14^, and we sought to combine this method with the double tap method to improve HDR efficiencies even further. Therefore, we tested the double tap method at the *FANCF*, *APOB1*, and *MMACHC* sites (which we previously tested without blocking mutations, Fig. 1c) using ssODNs identical to those used previously but with additional mutations incorporated to block re-cleavage of the target genomic locus by Cas9 after a successful editing event. Consistent with prior studies, we found that the use of blocking mutations boosted HDR yields considerably (Figs. 1c and 2a, and Supplementary Fig. 4). Furthermore, we found that the use of secondary gRNAs facilitated similar fold-improvements in HDR efficiencies as we observed previously when using ssODNs without blocking mutations. Specifically, double tapping produced a 1.1 ± 0.1-fold improvement at the *FANCF* site (compared to 1.1 ± 0.1-fold when using an ssODN lacking a blocking mutation), a 1.4 ± 0.1-fold improvement at the *APOB1* site (compared to 1.8 ± 0.4-fold with an ssODN lacking a blocking mutation), and a 1.6 ± 0.1-fold improvement at the *MMACHC* site (compared to 2.0 ± 0.2-fold with an ssODN lacking a blocking mutation). When comparing HDR efficiencies of samples with primary gRNAs only used with ssODNs without blocking mutations to samples with secondary gRNAs used with ssODNs with blocking mutations, we observed a 14.4 ± 1.2-fold improvement at the *FANCF* site, a 53.9 ± 5.8-fold improvement at the *APOB1* site, and a 6.1 ± 0.7-fold improvement at the *MMACHC* site (Supplementary Fig. 4). While the double tap method can be used independently to improve HDR yields without requiring additional mutations, these data demonstrate that the double tap method can be combined with blocking mutations to further improve HDR efficiencies. Importantly, in both cases HDR rates are improved without perturbing gene expression levels or the cell cycle.

**Fig. 3:**
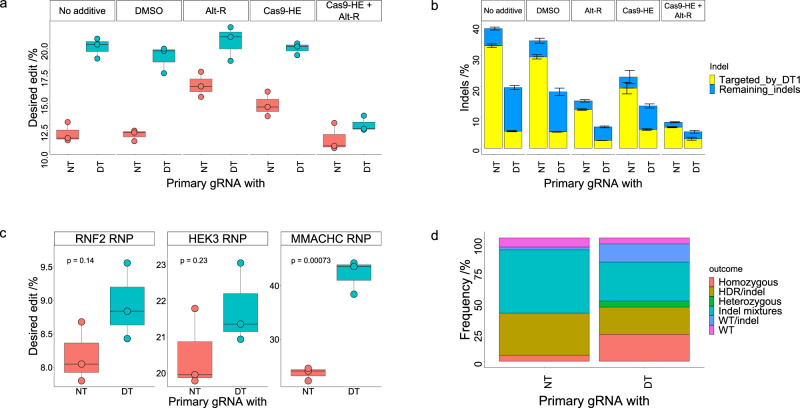
Further characterization of the double tap method. **a** Additive effects of double tap and previously developed HDR-improving methods were investigated at the *MMACHC* site. Shown are the percent of DNA sequencing reads with the desired modification introduced (perfect HDR products without indels) for cells treated with primary gRNA and a non-targeting gRNA (NT, left), or primary gRNA and secondary gRNA (DT, right; only one secondary gRNA was used at the *MMACHC* site). NT and DT samples were additionally treated with the small molecule HDR enhancer (Alt-R) or with a Cas9-CtIP fusion construct (Cas9-HE). DMSO-treated and no additive samples served as a base line for comparison (the Alt-R molecule is dissolved in a DMSO solution). **b** Shown are total indel rates of samples from (**a**), with the specific indels targeted by the secondary gRNA shown in yellow. Blue represents indels not targeted by a secondary gRNA. Values and error bars represent the mean of the number of sequencing reads with indel sequences divided by the total number of sequencing reads ± SD for *n* = 3 biological replicates. **c** Double tap improvements using Cas9:gRNA RNP complex at 3 sites. Shown are the percent of DNA sequencing reads with the desired modification introduced (perfect HDR products without indels) for cells treated with primary gRNA and a non-targeting gRNA (NT, left), or primary gRNA and secondary gRNA(s) (DT, right; three secondary gRNAs were used at the *RNF2* site, two secondary gRNAs were used at the *HEK3* site, and one secondary gRNA was used at the *MMACHC* site). **d** Analysis of zygosity of genome edited isogenic cells (*n* = 41 for each groups) at the *MMACHC* locus. Shown are the frequency of the indicated genome editing outcomes from each set of edited cells. Samples in (**a**–**c**) were analyzed by NGS after 72 h, and samples in (**d**) were clonally expanded and genotyped by NGS after 3 weeks. **a**, **c** Values on the whisker plots represent the lowest observation, lower quartile, median, upper quartile and the highest observation of three independent replicates. Data were analyzed with univariate statistics (one-way ANOVA [one-sided]) and *p* values are labeled on the graphs.

To further investigate potential synergistic effects of the double tap method with existing methods to improve HDR:NHEJ ratios, we compared and combined the double tap method with a small molecule inhibitor of NHEJ and a Cas9-CtIP fusion construct. Specifically, we used IDT’s “Alt-R ^TM^ HDR Enhancer V2” (which we will refer to as Alt-R) and the Cas9-HE fusion protein (wherein Cas9 is tethered to the HDR enhancer domain of the CtIP protein). We tested these strategies at the *MMACHC* site using primary gRNA with additional non-targeting or secondary gRNA to compare them to and evaluate their additive effects with the double tap method. Both the Alt-R molecule and the Cas9-HE increased HDR rates relative to the wild-type Cas9 (wtCas9) with primary and non-targeting gRNA sample with no additives or dimethyl sulfoxide (DMSO) added (the Alt-R molecule is dissolved in a DMSO solution, Fig. 3a). Specifically, we observed a 1.4 ± 0.1-fold improvement with the Alt-R sample and a 1.2 ± 0.1-fold improvement with the Cas9-HE sample relative to the no additive sample (which was within error of the DMSO sample). Notably, both samples had absolute HDR rates below that of the wtCas9 double tap sample with no additives (which improved the HDR rate 1.7 ± 0.1-fold compared to the wtCas9 primary and non-targeting gRNA sample, Fig. 3a). Both methods decreased overall indel rates as well (from 38.6 ± 0.5% to 15.3 ± 0.4% with the Alt-R, and to 23.0 ± 2.2% with Cas9-HE), resulting in similar overall indel rates to the wtCas9 double tap sample with no additives (Fig. 3b). However, we will note that we targeted a particularly high efficiency indel with a secondary gRNA at this site, and other sites with lower efficiency indels may benefit more from the Alt-R molecule of Cas9-HE than they would from the double tap method. Interestingly, combining both HDR enhancer methods (Alt-R and Cas9-HE) with each other did not improve HDR rates (1.0 ± 0.1-fold improvement to the no additive sample, Fig. 3a). Significantly, the combination of either the Alt-R molecule or the Cas9-HE construct with the double tap method further increased precision genome editing compared to their respective primary and non-targeting gRNA sample. Specifically, we observed a 1.7 ± 0.2-fold improvement with the Alt-R double tap sample and a 1.7 ± 0.1-fold improvement with the Cas9-HE double tap sample relative to the no additive sample (which are both within error of the double tap sample with no additives, but the overall indel rates were decreased in these combination treatments, Fig. 3a, b). These combinations additionally further reduced the overall indel rates compared to the no additive double tap sample. In particular, the Alt-R double tap combination yielded the lowest overall indel rates (6.9 ± 0.3%, Fig. 3b). We will note however that usage of the Alt-R molecule induced changes in the morphology of the cells (Supplementary Fig. 5). As these methods manipulate the cell cycle and/or expression levels of DNA repair pathways, the cells’ ability to perform native DNA repair functions may be impaired, leading to additional, unwanted genomic modifications elsewhere in the genome. This may be responsible for the significantly reduced editing yields in the Alt-R Cas9-HE combination samples. Importantly, these data show that the double tap method can be combined with additional HDR-enhancing methods to further improve precision genome editing rates, and decrease the rates of unwanted indels.

### Double tap using Cas9 ribonucleoprotein (RNP) complexes

The Cas9:gRNA complex is often delivered into cells as a ribonucleoprotein (RNP) complex due to lower toxicity, decreased off-target editing efficiencies, and enhanced on-target editing efficiencies. To assess if RNP delivery is compatible with the double tap method, we transfected HEK293T cells with purified Cas9 RNP complexes targeting the *HEK3*, *RNF2*, or *MMACHC* sites (using the same primary gRNA and non-targeting or secondary gRNA(s) as used previously) and the same ssODNs as used previously. We observed similar results when utilizing RNP delivery as we did when using plasmid-based delivery; HDR rates increased and rates of indel products decreased (Fig. 3c). The average HDR-mediated genome editing efficiencies improved 1.1 ± 0.1-fold at the *HEK3* site, 1.1 ± 0.1-fold at the *RNF2* site, and 1.8 ± 0.1-fold at the *MMACHC* site. We also observed a decrease in overall indel rates for double tap samples, driven by large decreases in introduction efficiencies of the specific indels targeted by the secondary gRNAs. Specifically, the collective indel frequencies of the indels targeted by secondary gRNAs decreased from 3.0 ± 0.1% to 0.2 ± 0.05% at the *HEK3* site, from 21.9 ± 0.9% to 10.6 ± 0.4% at the *RNF2* site, and from 40.5 ± 1.3 to 4.0 ± 0.4% at the *MMACHC* site (Supplementary Fig. 6).These data demonstrate that the double tap method can be implemented with RNP delivery to enhanced HDR efficiencies and decreases unwanted indel frequencies, albeit with slightly less drastic improvements as when using plasmid-based delivery.

### Analysis of zygosity of double tap edited cells

Isogenic cell lines are useful model systems with which to study the effects of mutations. Generation of such models can often be hampered by “hemizygous-like” clones, in which one allele contains the edit of interest, and the other an indel^14^. Therefore, we sought to characterize the zygosity of cell lines generated using the double tap method. HEK293T cells were transfected with ssODN, Cas9-p2A-GFP plasmid, and gRNA plasmids (primary gRNA with non-targeting or secondary gRNA plasmid) to target the *MMACHC* locus. After 72 h, individual GFP-positive cells were sorted into separate wells of a well-plate using fluorescence activated cell sorting (FACS) and clonally expanded. Cells were expanded for 21 days, and 41 colonies per experiment (non-targeting or secondary gRNA) were genotyped via NGS. As the HEK293T cell line is pseudotriploid^35,36^ (the *MMACHC* locus resides on chromosome 1, which is triploid), a variety of zygosities were observed. We have simplified them into the categories of homozygous (all copies have the HDR edit, with no indels), heterozygous (mixture of wild-type and HDR edits, with no indels), HDR/indel products (mixture of HDR edits and indels), indel mixtures (all copies have indels), WT/indel (mixture of wild-type and indels), and WT (all copies unedited). The breakdown can be seen in Fig. 3d. Importantly, while only two homozygous colonies were identified from the primary plus non-targeting gRNA samples, nine were identified from the double tap samples, representing an increase in frequency from 5% to 22% using the double tap method, or a >4-fold improvement. Consistent with previous reports^14^, no heterozygous clones were obtained from the primary plus non-targeting gRNA sample. However, we were able to obtain 2 heterozygous clones in our double tap samples, which may have originated from a WT/indel-containing cell. We also observed large decreases in the number of clones with indel mixture genotypes and HDR/indel genotypes in the double tap samples compared to the primary plus non-targeting samples (Fig. 3d). Specifically, the frequency of indel mixture colonies decreased from 51% to 32%, and the HDR/indel- mixed genotype clone frequency decreased from 34 to 22%. These data show that the double tap method can be used to improve the frequency of homozygous and heterozygous isogenic clones when using HDR to generate disease-relevant model systems.

### Double tap using dsDNA donor templates to perform gene knock-in

**Fig. 4:**
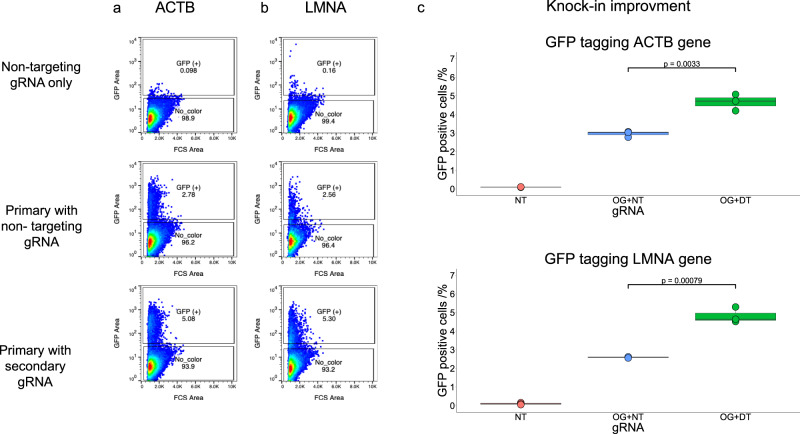
Improvements in gene knock-in with dsDNA donor templates using the double tap method. Selected scatter plots of GFP fluorescence (y-axis) and cell forward scatter (x-axis), showing gating for GFP fluorescence for HEK293T cells transfected with plasmids encoding dsDNA donor template, Cas9, and non-targeting gRNA only (top), primary and non-targeting gRNAs (middle), or primary and secondary gRNAs (bottom) for the *ACTB* gene (**a**) and the *LMNA* gene (**b**). **c** Quantification of the percent of cells with GFP fluorescence in the *GFP* knock-in experiment for the *ACTB* (top) and *LMNA* (bottom) genes. NT stands for non-targeting, OG + NT stands for primary with non-targeting, and OG + DT stands for primary and gRNAs. One secondary gRNA was used at both sites. Values on the whisker plots represent the lowest observation, lower quartile, median, upper quartile and the highest observation of three independent replicates. Data were analyzed with univariate statistics (one-way ANOVA [one-sided]) and *p* values are labeled on the graphs.

The installation of small modifications is typically carried out using ssODNs as a donor template. However, the introduction of larger (typically, >100 bps) modifications, such as knocking-in a gene to a targeted locus, is usually carried out using dsDNA donor templates. These two precision genome editing methods have been shown to function via different mechanisms (ssODN-mediated knock-in occurs in a Rad51-independent manner, while dsDNA donor-mediated knock-in occurs in a Rad51-dependent manner^7^). We therefore sought to determine if the double tap method was compatible with both. We used the double tap method to knock-in the green fluorescent protein (*GFP*) gene just after the start codon of two different genes (*ACTB* and *LMNA*) using dsDNA donor plasmids. We used donor template and primary gRNA designs that had been described previously for *ACTB*^25^, as well as for *LMNA*^27^. To design secondary gRNAs, we first transfected HEK293T cells with plasmids encoding Cas9 and primary gRNA, then analyzed the genomic loci of interest with NGS after 72 h to determine the indel product distribution (Supplementary Fig. 7). We designed one secondary gRNA for each site, as the initial rates of the most frequent indel product at each site was >4 times larger than that of the next most frequent indel product (Supplementary Fig. 8). We then transfected HEK293T cells with plasmids encoding the dsDNA donor, Cas9, and gRNA (either non-targeting gRNA only, primary and non-targeting gRNAs, or primary and secondary gRNAs). Knock-in of *GFP* was monitored by flow cytometry fourteen days post-transfection, after continuous passaging of the cells. At this time, all negative control samples (untransfected cells, and cells transfected with Cas9, dsDNA donor, and non-targeting gRNA only) showed minimal GFP fluorescence (<0.2% of cells with GFP fluorescence). *GFP* knock-in to the *ACTB* gene increased 1.6 ± 0.1-fold when using the double tap method, and *GFP* knock-in to the *LMNA* gene increased 1.9 ± 0.1-fold when using the double tap method (Fig. 4). These data show that the double tap method can be used successfully independently of the donor template type (ssODNs and dsDNA templates).

### Double tap in K562 and HeLa cell lines

We then tested the double tap method in human erythroleukemic (K562) and human cervical cancer (HeLa) cell lines using the *APOB1* and *MMACHC* primary gRNAs and secondary gRNAs that we previously validated in HEK293T cells. Cells were transfected with ssODN, Cas9-p2A-GFP plasmid, and gRNA plasmids. After 72 h, GFP positive cells were enriched using fluorescence activated cell sorting (FACS) and analyzed by NGS (FACS enrichment was used due to the significantly lower transfection efficiencies of these cell lines as compared to HEK293T cells).

**Fig. 5:**
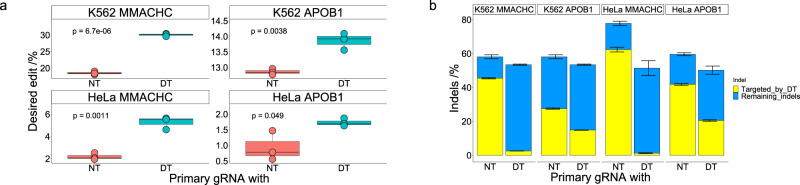
Improvements in HDR-mediated genome editing with ssODNs using the double tap method in human erythroleukemic (K562) and human cervical cancer (HeLa) cell lines. **a** HeLa or K562 cells were transfected with ssODN, Cas9-p2A-GFP plasmid, and gRNA plasmids. After 72 h, cells were enriched with FACS and analyzed by NGS and HDR-mediated genome editing efficiencies were quantified. Shown are the percent of DNA sequencing reads with the desired modification introduced (perfect HDR products without indels) for cells treated with primary gRNA and a non-targeting gRNA (NT, left), or primary gRNA and secondary gRNA(s) (DT, right; one secondary gRNA was used at both sites). Data from the *MMACHC* site are on the left and those from the *APOB1* site are on the right. Data acquired from K562 cells are on the top and those from HeLa cells are on the bottom. Values on the whisker plots represent the lowest observation, lower quartile, median, upper quartile and the highest observation of three independent replicates. Data were analyzed with univariate statistics (one-way ANOVA [one-sided]) and *p* values are labeled on the graphs. **b** Shown are total indel rates of all samples, with the specific indels targeted by secondary gRNAs shown in yellow. Blue represents indels not targeted by secondary gRNAs. Values and error bars represent the mean of the number of sequencing reads with indel sequences divided by the total number of sequencing reads ± SD for *n* = 3 biological replicates. Data points are marked as circles when the ssODN encoded an extra blocking mutation, and as triangles when no additional mutation was installed.

At the *MMACHC* site, the average HDR-mediated genome editing efficiency improved 1.6 ± 0.04-fold in K562 cells and 2.4 ± 0.3-fold in HeLa cells (compared to 1.6 ± 0.1-fold in HEK239T cells, Fig. 5a). At the *APOB1* site, the average HDR-mediated genome editing efficiency improved 1.1 ± 0.02-fold in K562 cells and 1.9 ± 0.8-fold in HeLa cells (compared to 1.4 ± 0.1-fold in HEK239T cells, Fig. 5a). We attribute the slight differences in fold-change values for a given target site among the different cell lines to the differences in initial rates of the double tap-targeted indels (Figs. 2d and 5b). While in general indel sequences are reproducible among different cell lines, their relative introduction rates will fluctuate^32^ (Supplementary Fig. 9), which will impact the effect of the double tap method. Furthermore, we again observed near complete disappearance of the double tap-targeted indel at the *MMACHC* site (in HeLa cells, the rate of this indel dropped from 62.3 ± 1.4% to 1.2 ± 0.3% after double tapping, with similar results in K562 cells Fig. 5b), while the indel at the *APOB1* site persisted (in HeLa cells, the rate of this indel dropped from 41.8 ± 0.6% to 20.5 ± 0.4% after double tapping, with similar results in K562 cells, Fig. 5b). This incomplete disappearance of the *APOB1* double tap-targeted indel is potentially responsible for the reduced fold-improvement observed at this site, and suggests that the double tap method is most effective when targeting indel sequences with high rates, and when the corresponding secondary gRNAs are highly efficient at targeting their respective sequences. We used CRISPick^37^ to analyze the predicted efficiencies of all secondary gRNAs, but did not find the efficiency score of the *APOB1* secondary gRNA to be significantly lower than those of the secondary gRNAs that effectively targeted their respective indels. We therefore recommend explicitly testing all secondary gRNAs for efficiency, and re-designing if necessary. In the case of the *APOB1* secondary gRNA, an alternative protospacer/PAM could be used to target this indel (Supplementary Fig. 10), and may facilitate greater fold-improvements. Importantly, these data show that the double tap method can be used in a variety of human cell lines.

### Disease modeling and comparison to prime editing

We next tested the ability of the double tap method to install two disease-relevant mutations to demonstrate its utility for generating disease models and to compare its performance with that of prime editing. We chose the sickle cell-relevant mutation E6V in hemoglobin, which is an A to T transversion mutation in the *HBB* gene, and the Tay-Sachs disease-relevant TATC 4-bp insertion in the *HEXA* gene, as pegRNA-nicking gRNA combinations have already been optimized to introduce these mutations with prime editing. Five potential primary gRNAs (referred to as *HBB1*, *HBB2*, etc. and *HEXA1*, *HEXA2*, etc. primary gRNAs) were designed for each site using inDelphi to aid in identifying “high precision” protospacers (i.e. those predicted to produce outcomes in which the top three indel sequences would represent >40% of products) with cut sites within 15 bp of the intended mutation (Supplementary Fig. 11). We transfected HEK293T cells with plasmids encoding Cas9-NG (a variant of Cas9 that has a relaxed PAM requirement of NG instead of NGG) and each of these candidate primary gRNAs, lysed the cells after 72 h, and analyzed genomic loci of interest with NGS and CRISPResso2. The total indel rates, as well as the individual introduction efficiencies of the top three indel sequences acquired with each of the candidate primary gRNAs are shown in Supplementary Fig. 8a. The sequences and efficiencies of the individual indels, along with the inDelphi predictions, are shown in Supplementary Fig. 1. We found that five out of the ten protospacers closely matched the inDelphi predictions; that is, these gRNAs (*HBB1*, *HBB3*, *HEXA2*, *HEXA4*, and *HEXA5*) generated the top three inDelphi predicted indels, and their collective introduction efficiencies represented >40% of all repair products. One protospacer (*HBB4*) was inefficient and therefore precluded an accurate analysis of indel products, two protospacers (*HBB2* and *HEXA1*) produced the top three inDelphi predicted indels, but their collective introduction efficiencies represented <25% of all repair products, and two protospacers (*HBB5* and *HEXA3*) produced only one or two of the top three inDelphi predicted indels. Overall, we recommend using inDelphi to guide protospacer design for identifying “high precision” protospacers, but additionally recommend testing multiple gRNAs for a given target site given the 50% success rate we observed here (and with the protospacers we tested earlier, discussed later). We chose two primary gRNAs per site to proceed with preliminary double tap experiments; primary gRNAs that produced high frequencies of a (preferably) single indel product were chosen (the *HBB1*, *HBB3*, *HEXA2*, and *HEXA5* primary gRNAs). We then designed ssODNs compatible with both primary gRNA options for each site (cut sites were within 15 bases of each other). In the case of the *HBB* mutation, we added a silent blocking mutation to boost HDR efficiencies. For the *HEXA* mutation, additional silent mutations were not deemed necessary as the 4-bp insertion disrupted both protospacers (details on ssODNs are listed in Supplementary Data 1). To assess initial HDR efficiencies when using these primary gRNAs, we transfected HEK293T cells with ssODNs and plasmids encoding Cas9 and primary gRNA. After 72 h, cells were lysed and analyzed via NGS and CRISPResso2 to determine HDR and indel introduction efficiencies. We observed a low (<5%) HDR efficiency with the *HBB3* primary gRNA (Supplementary Fig. 8b), so we repeated the experiment to assess the initial HDR efficiency with the next best candidate primary gRNA (the *HBB5* primary gRNA). The initial HDR efficiency with the *HBB5* primary gRNA was almost 3-fold higher, so we proceeded with this primary gRNA. Indeed, using the equation from Fig. 2b, we would estimate an improvement of 1.6-fold for *HBB3* (which would result in an increase in HDR efficiency from 4.6% to 7.4%), and an improvement of 1.3-fold for *HBB5* (which would result in an increase in HDR efficiency from 11.6% to 15.3%). This experiment highlights the importance of balancing the initial HDR efficiency with the indel distribution of a putative primary gRNA when assessing its potential for the double tap method. We then designed one secondary gRNA for both *HEXA* primary gRNAs, one secondary gRNA for the *HBB1* primary gRNA, and three secondary gRNAs for the *HBB5* primary gRNA (Supplementary Fig. 9).

**Fig. 6:**
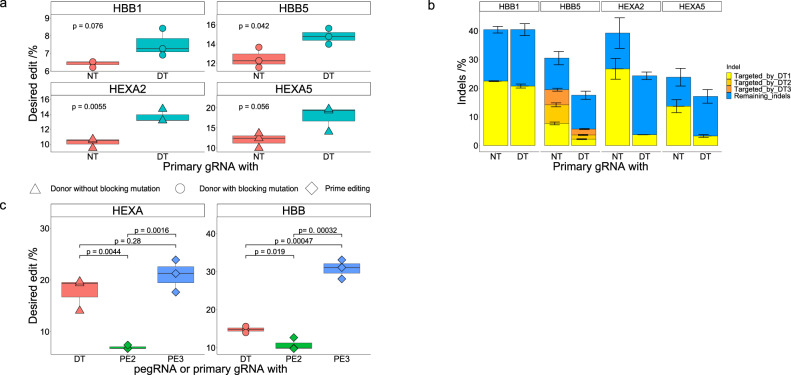
Installation of disease relevant mutations in the HBB and HEXA genes using the double tap method. **a** Shown are the percent of DNA sequencing reads with the desired modification introduced (perfect HDR products without indels) for cells treated with primary gRNA and a non-targeting gRNA (NT), or primary gRNA and secondary gRNA(s) (DT; three secondary gRNAs were used at the *HBB5* site, and one secondary gRNA was used at the *HBB1, HEXA2* and *HEXA5* sites). **b** Shown are total indel rates of all samples from (**a**), with the specific indels targeted by secondary gRNAs shown in yellow, orange, and red (depending on how many secondary gRNAs were used for a particular site, there may only be yellow bars). Blue represents indels not targeted by secondary gRNAs. **c** HEK293T cells were transfected with plasmids encoding the prime editor and pegRNA only (PE2 sample), or pegRNA and nicking gRNA (PE3 sample) to introduce the same mutations as in (**a**). After 72 h, cells were analyzed by NGS to determine the efficiencies of introduction of the intended edit. Shown are the percent of DNA sequencing reads with the desired modification introduced (perfectly edited products without indels) for double tap samples from (**a**) (labeled as DT), PE2 treated cells (labeled as PE2), or PE3 treated cells (labeled as PE3). Values on the whisker plots in (**a**) and (**c**) represent the lowest observation, lower quartile, median, upper quartile and the highest observation of three independent replicates. Data were analyzed with univariate statistics (one-way ANOVA [one-sided]) and *p* values are labeled on the graphs. Values and error bars in (**b**) represent the mean of the number of sequencing reads with indel sequences divided by the total number of sequencing reads ± SD for *n* = 3 biological replicates. Data points are marked as circles when the ssODN encoded an extra blocking mutation, and as triangles when no additional mutation was installed. Data points are marked as squares for prime editing samples.

We next transfected HEK293T cells with ssODNs and plasmids encoding Cas9, primary gRNA, and either non-targeting gRNA or secondary gRNA(s). After 72 h, cells were lysed and analyzed via NGS and CRISPResso2 to determine HDR and indel introduction efficiencies. We observed improvements in all four double tap samples compared to samples without secondary gRNAs. Using the equation from Fig. 2b, we first calculated a rough estimate of the expected improvement. While a perfect match was not expected as the coefficient of determination (R^2^) was only 0.81, improvements of 1.3-fold for the *HBB1* secondary gRNA, 1.3-fold for the *HBB5* secondary gRNAs, 1.4-fold for the *HEXA2* secondary gRNA, and 1.2-fold for the *HEXA5* secondary gRNA were calculated given the initial indel rates of the respective indels targeted with these secondary gRNAs. We observed improvements of 1.2 ± 0.1-fold for the *HBB1* secondary gRNA, 1.2 ± 0.1-fold for the *HBB5* secondary gRNAs, 1.3 ± 0.1-fold for the *HEXA2* secondary gRNA, and 1.5 ± 0.3 fold for the *HEXA5* secondary gRNA (Fig. 6a), all of which are within error of the calculated values. As with previous experiments, we observed decreases in both the total indel frequencies as well as the efficiencies of the specific indels targeted by the secondary gRNAs in all cases except the *HBB1* sample (Fig. 6b). The average absolute total indel frequency as well as that of the double tap targeted indel did not change in this sample, suggesting the secondary gRNA may be inefficient at facilitating Cas9 binding and/or cleavage (although we did observe an increase in the HDR efficiency for this sample). However, in all other samples we observed robust decreases in overall indel frequencies (a decrease from 30.5 ± 2.3% to 17.6 ± 1.4% for the *HBB5* secondary gRNAs sample, from 30.5 ± 2.3 to 17.6 ± 1.4% for the *HEXA2* secondary gRNA sample, and from 39.2 ± 5.3% to 24.3 ± 1.3% for the *HEXA5* secondary gRNA sample). These data further demonstrate the ability of the double tap method to simultaneously enhance HDR-mediated genome editing efficiencies and decrease overall indel rates using gRNAs targeted to high frequency indel sequences. Furthermore, this increase in genome editing precision does not require cell perturbations of any kind and can easily be implemented by simply including additional gRNAs in classic HDR experiments. Importantly, the enhanced precision of this method will greatly aid researchers with generating disease models.

We next compared the performance of the double tap method to that of prime editing. We used previously reported pegRNAs and nicking gRNAs to install these mutations^24^. It is important to mention that these two pegRNA-nicking gRNA combinations were extensively optimized; specifically, to identify the *HEXA* combination, the authors tested 43 pegRNAs and three nicking gRNAs (for a total of 129 different combinations tested). In contrast, for the double tap method, only five primary gRNAs were screened per site, and all double tap experiments that were performed displayed improvements in HDR efficiency. We transfected HEK293T cells with plasmids encoding PE2 and pegRNA only (PE2 sample), or pegRNA and nicking gRNA (PE3 sample). After 72 h, cells were lysed and analyzed via NGS and CRISPResso2 to determine the efficiency of introduction of the intended edit. We found that intended edit introduction efficiencies with PE2 were lower than that with the double tap method (10.8 ± 1.3% at the *HBB* site, and 7 ± 0.3% at the *HEXA* site), while those with PE3 were similar at the *HEXA* site (20.9 ± 2.6%), and higher at the *HBB* site (30.8 ± 2.1%, Fig. 6c). These results demonstrate the utility and simplicity of the double tap method for disease modeling.

### Off-target editing assessment

**Fig. 7:**
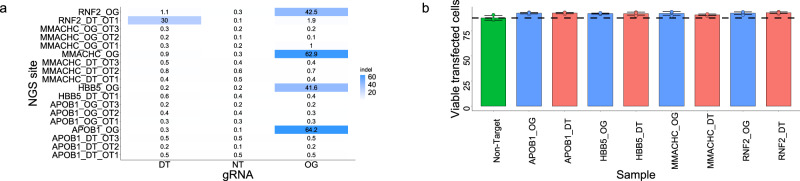
Assessment of off-target editing due to the double tap method. **a** HEK293T cells were transfected with Cas9 and gRNA plasmids (non-targeting, primary, or secondary gRNAs). After 72 h, cells were analyzed by NGS at the primary (on-target) and all predicted off-target loci. Shown are total indel rates of all samples. The primary (on-target) loci are labeled as OG, while predicted off-target sites for primary gRNAs are labeled as OG_OT, and predicted off-target sites for secondary gRNAs are labeled as DT_OT on the y axis. The label on the x-axis indicates which gRNA the cells were transfected with; the secondary (DT), non-targeting (NT) or primary (OG). Only one gRNA was used at a time, **b** HEK293T cells were transfected with plasmids encoding Cas9-p2A-GFP, primary gRNA, and either non-targeting gRNA or secondary gRNA(s). As a control, HEK293T were transfected with plasmids encoding Cas9-P2A-GFP and non-targeting gRNA only. After 72 h cells were stained with propidium iodide to quantify cell viability FACS. The percentage of transfected cells (as determined by GFP fluorescence) that were viable are plotted with respect to the primary gRNA used (*RNF2*, *HBB5*, *APOB1*, and *MMACHC*). Samples with primary and non-targeting gRNAs are shown in blue, while those with primary and secondary gRNAs are in pink. Three secondary gRNAs were used with the *RNF2* and *HBB5* primary gRNA, and one secondary gRNA was used at *APOB1* and *MMACHC* sites. The sample with non-targeting gRNA only is in green. Values and error bars represent the mean and standard deviation of viable cells within the transfected population for *n* = 3 biological replicates.

We recognized that a potential drawback of the double tap method is the possibility of introducing DSBs at additional off-target sites compared to when only a single gRNA is used. Indeed, the introduction of multiple DSBs within a given cell can cause cytotoxicity and chromosomal rearrangements^38–41^. Therefore, we first analyzed all secondary gRNAs used in this study for potential full matches with other sites in the human genome. We found only one secondary gRNA (one of the *RNF2* secondary gRNAs) that fully matched a location in the human reference genome that is directly next to an NGG PAM sequence (this locus is labeled *RNF2_DT_OT1*). To quantify editing at this site, we transfected HEK293T cells with plasmids encoding Cas9 and either a non-targeting gRNA, the *RNF2* primary gRNA, or the *RNF2* secondary gRNA, then lysed cells after 72 h and analyzed the primary on-target and the secondary matched loci of all samples for indel frequencies using NGS and CRISPResso2. Unsurprisingly, we observed a 30% indel introduction efficiency with the *RNF2* secondary gRNA at its fully matched locus (Fig. 7a). Additionally, the *RNF2* primary gRNA (which differs from the *RNF_DT_OT1* locus sequence by a 1-bp deletion) introduced indels at this locus with an efficiency of 1.9% (Fig. 7a). These data demonstrate that secondary gRNAs should always be analyzed for matching sequences elsewhere in the genome when using this method. When this occurs, we recommend using another PAM sequence nearby (if possible) to target a given indel sequence (Supplementary Fig. 10).

We additionally analyzed all secondary gRNAs for putative off-targets containing a single mismatch using Cas-OFFinder^42^, as these types of off-targets are the most common^43^. We found that only one secondary gRNA (one of the *HBB5* secondary gRNAs*)* had a potential off-target with a single mismatch (this locus is labeled as *HBB5_DT_OT1*). We then chose two additional sets of primary and secondary gRNAs (those for the *APOB1* and *MMACHC* sites) and identified and analyzed their predicted off-target sites in silico using a combination of Cas-OFFinder (to identify putative off-target sites with up to five mismatches and three bulges)^42^ and Benchling^44^ (to assess their predicted off-target scores). The *MMACHC* primary gRNA had the highest predicted off-target site (labeled as *MMACHC_OG_OT1*) with only a single mismatch, and a predicted off-target score of 100 (out of a highest possible score of 100). All other putative off-target sites had predicted off-target scores of less than 6 (the closest predicted off-target sites had at least two mismatches). Nevertheless, we selected three predicted off-target sites for each gRNA based off these two analyses for both the primary gRNAs (which we call the original guide off-target sites, or OG_OT), as well as for the secondary gRNAs (which we call the double tap guide off-target sites, or DT_OT, Supplementary Fig. 11). We transfected HEK293T cells with plasmids encoding Cas9 and either a non-target gRNA, the primary gRNA, or the secondary gRNA. Cells were lysed after 72 h, and the on-target and all off-target loci were analyzed for indel frequencies using NGS and CRISPResso2. To our surprise, we did not observe indel rates above non-targeting controls at any off-target loci (Fig. 7a). While an unbiased off-target identification method (such as GUIDE-seq, Digenome-seq, or DISCOVER-seq^30,43,45^) is required to fully evaluate the extent of off-target editing with the double tap method, these data suggest that the extent of off-target editing with the double tap method is similar to that of experiments using single gRNAs, unless the secondary gRNA fully matches a site in the genome.

We additionally sought to quantify cell viability following use of the double tap method. As previously stated, multiplexed DSB introduction can cause cytotoxicity; we therefore reasoned if the use of secondary gRNAs causes significant off-target editing, we would observe reduced viability of the cells. We chose primary and secondary gRNAs for the *RNF2* (in which case we used all three secondary gRNAs, including the one that has a fully matched site in the genome), *HBB5*, *APOB1* and *MMACHC* sites, as these had been previously evaluated for indel introduction efficiencies at putative off-target sites. We then transfected HEK293T cells with plasmids encoding Cas9-P2A-GFP (to allow for identification of transfected cells using GFP fluorescence) and gRNA (non-targeting gRNA only as a control, primary and secondary gRNA, or primary and non-targeting gRNA) and stained the cells with propidium iodide to monitor cell viability after 72 h (Fig. 7b). We did not observe any decrease in viability compared to the non-targeting gRNA samples; all samples had >80% total viability (Supplementary Fig. 12), with ≥90% viability of transfected cells (as determined by cells with GFP fluorescence, Fig. 7b), even with the RNF2 sample, which utilized three secondary gRNAs. These data show that the use of secondary gRNAs does not introduce off-target DSBs at a level that impacts cell viability.

Off-target editing remains a key challenge for all genome editing agents, and the use of high-fidelity Cas enzymes has been shown to alleviate off-target editing by CRISPR nucleases^46–51^. The use of these high-fidelity variants in combination with off-target score prediction software could minimize unwanted off-target editing for the double tap method. However, in silico off-target identification has major limitations, and thus in cases where off-target editing must be completely eliminated, the use of unbiased experimental methods to identify putative off-target edits would be required.

## Discussion

Here we describe the development and characterization of the double tap method to improve HDR-mediated genome editing efficiencies in human cell lines. The double tap method takes advantage of the modularity of the Cas9 system and the reproducibility of indel sequences by using additional secondary gRNAs that target unwanted, high-frequency indel sequences generated during the end-joining repair of DSBs. In this manner, the double tap method provides researchers with a second chance at a successful HDR event when performing precision genome editing at a locus of interest. Importantly, the double tap method does not perturb the cell by modulating gene expression levels or synchronizing the cell cycle phase which may introduction additional artifacts to the system being studied.

We sought to characterize the impact of the double tap method by first quantifying the improvements in HDR-mediated genome editing efficiencies following the use of secondary gRNAs targeted to indel sequences with a wide range of frequencies (ranging from 4.8 ± 0.2% to 49.2 ± 3.7%). We found a direct correlation between the fold-improvement afforded by this method and the collective frequencies of the indels targeted by secondary gRNAs; this correlation allows a user to estimate a fold-change in HDR efficiency for the double tap method following analysis of indel distribution frequencies for a particular gRNA of interest. We will note that initial HDR efficiencies can vary drastically depending on the primary gRNA used, and thus this value will need to be balanced with the estimated fold-change to identify the ideal conditions to maximize absolute HDR efficiencies. We found that overall indel rates also decreased when using the double tap method, mostly driven by large decreases in the frequencies of the indels that were targeted by secondary gRNAs. Overall, this led to enhancements in HDR:NHEJ ratios up to 3.8-fold. However, we will note that our targeted amplicon sequencing methods may miss larger deletion products that occur outside the sequencing primer binding sites.

The double tap method was found to be compatible with multiple cell lines, RNP delivery, and with both small modifications (using ssODN donors) and large insertions (using dsDNA donors). The design of secondary gRNAs is straight-forward when 1-bp insertions or deletions are targeted, in which case the original PAM can be used, and the resulting secondary gRNA will rarely match the original sequence. However, we found that in certain instances when small deletions (likely facilitated by MMEJ) were targeted, using the original PAM would result in a secondary gRNA that could target the original DNA sequence, but with an unwanted alternate cut site (Supplementary Fig. 13). In these cases, unwanted targeting should be avoided by using a secondary gRNA with an alternate PAM (see Supplementary Fig. 13 for an example). Overall, it is important to analyze each putative secondary gRNA for a full match with the original target sequence, or indeed with any other locations in the genome (as with our *RNF2* example, see below).

Overall, we tested the double tap method with 23 different primary protospacer sequences and compared their experimentally determined indel sequence distribution outcomes with their inDelphi predictions (Supplementary Fig. 1). Sixteen of our tested primary gRNAs are predicted to be “high precision” protospacers by inDelphi (i.e. those predicted to produce outcomes in which the top three indel sequences would represent >40% of products). Out of these 16 gRNAs, ten of them were experimentally determined to be “high precision”, with the same three inDelphi-predicted indel sequences representing >40% of repair products. Due to this high rate (>50%) of successfully predicting “high precision” sites, we recommend using inDelphi to guide the design of protospacers to use with the double tap method, but additionally recommend testing at least 2–3 primary gRNAs per experiment.

We have additionally demonstrated that the double tap method can be combined with existing HDR-enhancing methods to further improve precision genome editing efficiencies. Combining the use of secondary gRNAs with additional blocking mutations on the ssODN (to prevent Cas9 from re-cutting the target site after a successful HDR event) was found to produce additive improvements in HDR efficiencies. As neither of these methods disturb the cell cycle or DNA repair protein levels, this represents a simple and robust non-perturbative method for improving precision editing outcomes. We also demonstrated that the double tap method can be combined with DNA repair pathway alteration methods to achieve higher HDR:NHEJ ratios compared to using any of these strategies in isolation. The double tap method represents a simple yet effective strategy that can be effortlessly implemented into existing HDR-enhancing pipelines to further improve genome editing outcomes.

We additionally demonstrated the utility of the double tap method for generating of isogenic cell lines. Overall success rates of generating homozygous and heterozygous cell lines were improved, as the secondary gRNAs provides a “second chance” to convert indel-containing alleles into the desired edit. This improvement would allow for a decrease in the number of colonies screened during isogenic cell line generation, as well as an increase in the throughput of cell line generation, which is incredibly valuable for laboratories studying the functional effects of genetic variants. This method could be particularly useful for genome editing in organisms with high chromosomal copy numbers such as plants or applications that cannot take advantage of precision editing-enhancing strategies such as base editing, prime editing, and cell cycle/DNA repair manipulation, including gene drive applications. In fact in a complementary manuscript^52^ we have applied the double tap method to improve gene drive efficiencies by recycling resistance alleles. We demonstrated the utility of the double tap method by installing two disease-relevant mutations (an A to T point mutation in the *HBB* gene that causes sickle cell disease, and a 4-bp insertion in the *HEXA* gene that causes Tay-Sachs disease). For both mutations, we easily identified secondary gRNAs to boost HDR efficiencies. The double tap method can therefore be easily integrated into researchers’ current HDR experiments by simply analyzing their DNA sequencing data to identify high-frequency indel products. For experiments such as disease modeling (particularly for the generation of isogenic cell lines), absolute HDR rates are often the most important factor, and dictate whether homozygous variants can be obtained. The double tap method was shown to improve HDR yields up to 2.4-fold here, and because fold-changes can be estimated based on the initial indel frequencies, HDR rates can potentially be modulated if heterozygous models are desired. The decrease in indel rates facilitated by this method is also an important factor and can help to avoid generating cells in which the mutation of interest is present at one allele and an indel is present at the other. Enhancements in absolute HDR efficiencies are invaluable for modeling of polygenic disorders, in which the introduction of multiple mutations is necessary. In these cases, the increase in likelihood of successfully generating the model is proportional to the product of the individual increases in HDR rates for each mutation.

Off-target editing is always a factor to consider with genome editing experiments and the usage of additional gRNAs increases the number of potential off-target edits, and therefore the possibility of translocations, large-scale deletions, and chromothripsis. This scales with the number of gRNAs, thus experiments that require multiple secondary gRNAs have an increased probability of suffering from off-target issues. While in silico off-target prediction tools have been developed and can identify certain putative off-target loci for a given gRNA (including secondary gRNAs), for experiments in which off-target editing is unacceptable, each gRNA needs to be individually assessed using unbiased methods. High-fidelity Cas9 variants have also been used to reduce or eliminate off-target editing in DSB-reliant genome editing experiments, and we expect these mutants could also be used successfully with the double tap method. It is imperative to analyze secondary gRNAs to assess if they are a perfect match with other sites in the genome prior to using them. If this is the case, we recommend re-designing the secondary gRNA to use a different PAM nearby if this is possible (see Supplementary Fig. 10 for an example). Nevertheless, for each experiment, an analysis of the risks (in terms of potential off-target editing) versus the benefits (the extent to which a secondary gRNA could enhance the HDR efficiency) of the double tap method will need to be performed by the researcher.

There are now a variety of “next-generation” genome editing tools for researchers to choose from, such as base editors and prime editors, and each editor comes with its own unique pros and cons. Here we directly compared the double tap method to prime editing to introduce small modifications, and found that with minimal optimization, we could approach PE3 efficiencies and surpass PE2 efficiencies using this method. A drawback of prime editing is the requirement of extensive optimization of the length of the primer binding region and the reverse transcription template portions of the pegRNAs to find a combination with satisfactory efficiency for each protospacer option (and there are often multiple protospacer options for a given modification of interest). Additionally, again with minimal optimization, we were able to improve our efficiencies of GFP knock-in with the double tap method up to 90%. Next-generation genome editing technologies such as base editing and prime editing are unable to facilitate such large insertions. Overall, we believe a major benefit of the double tap method is the simplicity of its implementation; a handful of candidate primary gRNAs can be tested and analyzed for initial HDR efficiencies and indel distributions, and fold-changes can then be estimated to identify the optimal primary-secondary gRNA combination to maximize HDR yields. Overall, this significantly reduces the time and resources required for construct optimization as compared to prime editing.

In summary, the double tap method presents researchers with an easily implemented method to increase HDR-mediated genome editing efficiencies using a combination of a primary gRNA that produces high frequency indel products with a secondary gRNA that targets these indel sequences. A major benefit of this method is its ease of integration with any previously developed HDR system; minimal optimization is required. We anticipate that this method will aid researchers working in the fields of plant, mammalian cell, or animal genome editing.

## Methods

### Cloning and constructs

JDS246 (NGG-WT-Cas9, Addgene plasmid # 43861), pCMV_ABEmax_P2A_GFP (Addgene plasmid # 112101), pCMV-PE2 (Addgene plasmid # 132775), pFYF1320 (gRNA expression plasmid, Addgene plasmid # 47511), pX330 (Addgene plasmid # 42230), pCas9-HE (Addgene plasmid # 109400), and the donor plasmid for the *ACTB* knock-in experiments (AICSDP-15:ACTB-mEGFP, Addgene plasmid # 87425) were obtained from Addgene. pCMV_ABEmax_P2A_GFP was used as a template to create Cas9-P2A-GFP and Cas9-NG-P2A-GFP constructs using USER cloning, following New England Biolabs (NEB) protocols^53^. Sequences of all plasmids are available in Supplementary Data 1.

Two BsmbI (a type IIS restriction enzyme) recognition sites were installed into the spacer region of the pFYF1320 plasmid using USER cloning, following NEB protocols, to produce the gRNA destination vector pU6-sgRNA-BsmbI. Custom guide RNA plasmids for each target site were then generated from pU6-sgRNA-BsmbI using Golden Gate assembly protocols as described by NEB. Briefly, pU6-sgRNA-BsmbI was digested with BsMBI-v2 (NEB #0739) overnight following the manufacturer’s instructions. The digested backbone was gel purified using a QIAquick Gel Extraction kit (#QIAGEN 28704), and inserts encoding custom spacer sequences (sequences are available in Supplementary Data 1) were annealed and ligated into the backbone with T4 DNA ligase (NEB #M0202) following the manufacturer’s instructions. As GFP tagging of *LMNA* was previously done in our lab, those plasmids were cloned into a different backbone. The *LMNA* primary gRNA was cloned into the pX330 backbone (which has BbsI recognition sites), creating pU6_LMNA_SpCas9. Briefly, the pX330 backbone was digested with BbsI (NEB #R3539S) following the manufacturer’s instructions, gel extracted, and the annealed inserts encoding custom spacer sequences (sequences are available in Supplementary Data 1) were ligated into the digested, purified backbone with T4 DNA ligase. pLMNA_HA_donor_GFP plasmid was cloned in multiple steps: first the *LMNA* homology arms were amplified from genomic DNA using primers detailed in Supplementary Data 1, then the PCR product was TOPO cloned into the pCR2.1 TOPO backbone (ThermoFisher #K450002) to make a pLMNA_reservoir plasmid following the manufacturer’s instructions. The entirety of the pLMNA_reservoir plasmid was then amplified by PCR using primers detailed in the Supplementary Data 1, which created a linearized DNA product. The linearized product was assembled with TurboGFP (synthesized gene block – sequence is in Supplementary Data 1) using Gibson assembly following the NEB protocol #E2611.

Prime editing gRNAs were generated in two steps. First the spacer sequence was incorporated into the pU6-sgRNA-BsmbI plasmid as previously described to generate a stepping-stone plasmid, followed by incorporation of the reverse transcriptase template (RTT) and primer binding sequence (PBS) sequences using site directed mutagenesis. Site directed mutagenesis primers designed to install the RTT and PBS sequences (sequences are available in Supplementary Data 1) were obtained from integrated DNA technologies, and 5ʹ phosphorylated using T4 Polynucleotide Kinase (NEB #M0201) following the manufacturer’s instructions. PCR was then performed with Phusion High-Fidelity DNA Polymerase (NEB #M0530) with the phosphorylated primers and the stepping-stone plasmid as a template. PCR products were purified using the QIAquick PCR purification kit (QIAGEN #28104) following the manufacturer’s instructions. PCR products were ligated using Quick Ligase (NEB #M2200), and ligation products were transformed into NEB 10-beta (NEB #C3019H) cells following the manufacturer’s instructions. Endotoxin-free plasmids were prepared using either the Zymo mini (Zymo #D4037) or midiprep (Zymo #11-550B) kit following the manufacturer’s instructions. Plasmids generated using USER cloning were fully sequenced with Sanger sequencing, while gRNA plasmids generated using Golden Gate cloning were sequenced around the insert to confirm correct ligation. Protospacer sequences for all gRNA plasmids are available in Supplementary Data 1. The selected primary gRNAs were either previously used in prior publications^22,24,25,27,32^ or designed to have cut sites within 15 bp of the intended mutation and to be “high precision” protospacers by inDelphi (i.e. those predicted to produce outcomes in which the top three indel sequences would represent >40% of products).

### Cell culture and transfections

All cells were cultured at 37 °C with 5% CO_2_ in a humidified environment. HEK293T (ATCC CRL-3216), HeLa (ATCC CCL-2), and K562 (ATCC CCL-243) cells were obtained from ATCC. HEK293T and HeLa cells were maintained in Dulbecco’s Modified Eagle’s Medium (DMEM, Gibco #10566-016) supplemented with 10% (*V*/*V*) fetal bovine serum (FBS, Gibco #10437-028), while K562 cells were maintained in Roswell Park Memorial Institute 1640 (RPMI 1640, Gibco #11875-093) media supplemented with 10% (*V*/*V*) FBS. HEK293T and HeLa cells were plated at a density of 100,000 cells per well in 48-well plates in a total volume of 250 µL per well, and transfected four hours after plating using 1.5 µl Lipofectamine 2000 (Invitrogen #11668-019) and a custom DNA mixture (described below) in 25 µL total volume, made up with Opti-MEM (Gibco #31985-070). For PE2 experiments, 750 ng of PE2 plasmid and 250 ng of pegRNA plasmid were used per transfection. For PE3 experiments, 750 ng of PE2 plasmid, 250 ng of pegRNA plasmid, and 83 ng of nicking gRNA plasmid were used per transfection. For ssODN double tap experiments, 750 ng of Cas9-P2A-GFP plasmid (except for experiments involving the *SEC61B*, *HEXA*, and *HBB* loci, in which case Cas9-NG-P2A-GFP was used) or 750 ng of Cas9-HE plasmid, 300 ng of gRNA plasmid, and 10 nM final concentration of ssODN were used per transfection. The gRNA plasmid mixture was comprised of 200 ng of primary gRNA and 100 ng of non-targeting gRNA or secondary gRNA(s), except for non-targeting negative control samples, in which case 300 ng of non-targeting gRNA was used. For the *LMNA* knock-in experiment, Cas9 and primary gRNA were expressed from the same plasmid (pU6_LMNA_SpCas9). In this case, 1,000 ng of pU6_LMNA_SpCas9, 100 ng of non-targeting or secondary gRNA plasmid, and 300 ng dsDNA donor plasmid (pLMNA_HA_donor_GFP) was used. For the ACTB knock-in experiment, 750 ng of JDS246 plasmid (Cas9 expression without GFP), 300 ng of gRNA plasmid, and 300 ng of dsDNA donor plasmid was used. The gRNA plasmid mixture was comprised of 200 ng primary gRNA and 100 ng non-targeting or secondary gRNA. For off-target analysis experiments, 750 ng of Cas9- P2A-GFP plasmid and 200 ng of gRNA plasmid (either non-targeting gRNA, primary gRNA, or secondary gRNA only) was used. K562 cells were plated at a density of 1 × 10^6^ cells per well in 6-well plates in a total volume of 2.5 mL per well, and transfected four hours after plating using 15 µl Lipofectamine 2000 (Invitrogen #11668-019) and a custom DNA mixture (described below) in 250 µL total volume, made up with Opti-MEM (Gibco #31985-070). For these experiments, 3750 ng Cas9-P2A-GFP plasmid, 1500 ng gRNA plasmid, and 10 nM final concentration of ssODN were used per transfection. The gRNA plasmid mixture was comprised of 1,000 ng primary gRNA and 500 ng non-targeting or secondary gRNA. When the small molecule Alt-R ^TM^ HDR Enhancer V2 (Integrated DNA Technologies IDT #10007910) was tested, 0.435 µl of the Alt-R enhancer was diluted in Opti-MEM (Gibco #31985-070) to 25 µl and added immediately after the transfection. The same volume of DMSO was diluted in Opti-MEM (Gibco #31985-070) and added to a separate well as a control. The media was replaced 24 h after transfection to reduce cytotoxicity.

For the RNP transfections, Cas9 (TrueCut v2, #A36497) and custom TrueGuide synthetic sgRNAs (with the same spacer sequences that were used with the plasmid-based delivery samples, see Supplementary Data 1) were purchased from Thermo Fisher. Transfection was performed into HEK293T cells plated in 48 well as described above. First 750 ng TrueCut Cas9 was complexed with 4.5 pmoles TrueGuide gRNA. The gRNA mixture was comprised of 3 pmoles of primary gRNA and 1.5 pmoles of non-targeting gRNA or secondary gRNA(s). After RNP complex generation, ssODNs were added as described above (10 nM final concentration) and transfected with 1.5 µl Lipofectamine 2000 (Invitrogen #11668-019) with Opti-MEM (Gibco #31985-070) as described above. Samples from the ssODN experiments were harvested three days after transfection and processed for NGS analysis while *GFP* knock-in experiments were continuously passaged for fourteen days followed by flow cytometry analysis.

### Flow cytometry and fluorescence activated cell sorting (FACS)

HEK293T cells were analyzed via flow cytometry to assess *GFP* knock-in efficiency fourteen days after transfection. Cells were washed with 250 µL phosphate buffered saline (PBS, Gibco #10010-023) in the plate and then detached from the plate with Accumax (Innovative-Cell Technology #AM-105) according to the manufacturer’s instructions. After harvesting, cells were resuspended in 500 µL PBS. Samples were filtered into FACS tubes (Falcon, #352235) and kept on ice until analysis. A S3e cell sorter (Bio-Rad) equipped with 488 nm, 561 nm and 640 nm lasers was used for all analysis. The instrument was calibrated and quality control checked before each flow cytometry or FACS experiment. GFP positive samples were quantified using the 525/30 nm channel. Single color (pool of the transfected samples for each group) and no color (untransfected cells) control cell populations were used to set up gating. Single color (GFP positive cells for knock-in) had higher intensity than the untransfected cells for the corresponding channels (GFP channel for knock-in). We selected the GFP population based on untransfected cells. Gates were set up or checked with the untransfected and single color controls for each flow cytometry or FACS experiment. Example of the gates are shown in Supplementary Fig. 14. Doublets were gated out using forward and side scattering width against area, and 20,000 events were analyzed. HEK293T cell viability for off-target experiments was also analyzed via flow cytometry 72 h after transfection. Cells were washed with 250 µL PBS on the plate and then detached from the plate with Accumax (Innovative-Cell Technology #AM-105) according to the manufacturer’s instructions. After harvesting, cells were resuspended in propidium iodide staining buffer (PI, Invitrogen #1304MP) following the manufacturer’s instructions. Samples were filtered into FACS tubes and kept on ice until analysis. Cells stained with PI were quantified using the 615/25 nm channel and GFP samples were monitored on the 525/30 nm channel. Single color (non-transfected cells stained with PI, and separately transfected cells without PI staining) and no color (no transfection) control cell populations were used to set up gating. Doublets were gated out using forward and side scattering width against area, and 20,000 events were analyzed.

Isogenic cells for the zygosity experiment were generated using FACS. Cells were prepared for sorting as described above. Samples were gated against untransfected samples as described above. Single GFP positive cells (cells expressing Cas9) were sorted into 96 well plates 48 h post transfection using a BD AriaII cell sorter. Prior to sorting, wells were filled with 200 µL of 30% (*V*/*V*) FBS DMEM media and incubated at 37 °C. After sorting, plates were kept in the incubator for 3 weeks for clonal expansion, then harvested for NGS analysis.

All HeLa and K562 cell experiments required FACS (using GFP fluorescence) before NGS analysis. HeLa cells were prepared the same as the HEK293T cells described above. For K562 cells, cells were spun down at 300 g for 5 min, the supernatant was decanted, and cells were washed with another 500 µL PBS. Following the second wash, the cell pellets were resuspended in 500 µL PBS and kept on ice until sorting. The 525/30 nm channel was used to identify cells with GFP fluorescence, and untransfected cells were used as negative controls to set up gating. Doublets were gated out using forward and side scattering width against area, and 40,000 GFP positive cells were collected using purity mode. K562 cells were collected into RPMI 1640 supplemented with 20% (*V*/*V*) FBS, and HeLa cells were collected into DMEM supplemented with 20% (*V*/*V*) FBS. Both cell lines were then spun down, washed with 500 µL PBS, and then prepped for NGS.

### Next-generation sequencing

After 72 h of editing, cells were washed with PBS either on the plate (HEK239T cells) or after FACS (HeLa and K562 cells), followed by proteinase K digestion (in a buffer made up of 10 mM Tris, pH 7.5; 0.05% SDS, and 25 μg/mL freshly added proteinase K) at 37 °C for 1 h, followed by an 80 °C heat treatment for 30 min. HEK293T cells were digested in 100 µL total volume of buffer while the sorted HeLa and K562 cells were digested in 50 µL total volume of buffer. After the lysis, genomic loci of interest were PCR amplified using locus-specific primers (listed in Supplementary Data 1). These primers were designed to contain an adapter sequence, allowing for sample barcoding with a second round of PCR. PCR reactions were performed using Phusion High-Fidelity DNA Polymerase following the manufacturer’s instructions with the following modifications: all PCR reactions were performed using GC buffer, 3% DMSO was utilized, and 25% of the recommended primer amount was used to reduce the amount of primer dimers. 25 cycles of amplification were used for round one PCRs, while 15 cycles of amplification were used for round two PCRs. An annealing temperature of 61 °C, and an extension time of 45 sec was used for both rounds. In total, 0.5 μL of genomic DNA was used a template for round one PCRs, and 0.5 μL of round one PCR product was used as a template for round two PCRs at 10 μL total reaction volume. Second round PCR products were pooled together based on the amplicon size and purified from a 2% agarose gel using the QIAGEN gel extraction kit (QIAGEN #28704) following the manufacturer’s instructions. The resulting purified libraries were quantified with the Qubit dsDNA high sensitivity kit (Thermo Fisher #Q32851) and diluted to 1.8pM following Illumina’s sample preparation guidelines. The final library was mixed with 1.8pM PhiX in a nine to one ratio. Samples were then sequenced on a MiniSeq (Illumina) via paired end sequencing.

### Data analysis and statistics

NGS samples were processed in CRISPResso2^54^ (version 2.0.20b) using the default and HDR outputs. Values from the CRISPResso2 were further processed in R Studio (version 1.4.1717) and plotted with the “ggplot2”^55^ package. Univariate statistics were performed in R Studio using the “ggpubr” package. FACS data was analyzed with FlowJo (version 10.7.2) to assess knock-in efficiencies. InDelphi^32^ (version 0.18.1) was used to predict insertions and deletions at the Cas9 cut site. Indel frequency values and errors were calculated as follows: values represent the mean of the number of sequencing reads with the indel sequence of interest (or any indel, when calculating total indel rates) divided by the total number of sequencing reads ± standard deviation (SD) for *n* = 3 biological replicates. For biological replicates, cells were plated into three different wells on the same day. Transfection reagents were prepared in three different tubes and transfected into independent replicates. Day to day transfection variability (from different splits of the same HEK293T cells) is demonstrated in Figs. 2 and 3 at the *MMACHC* site.

Fold-change values and errors were calculated as follows: values represent the mean of the number of sequencing reads with perfect HDR outcomes divided by the total number of sequencing reads for double tap samples divided by that of the samples with primary and non-targeting gRNA ± propagation of uncertainty of the SD for *n* = 3 biological replicates.

Percent decrease values and errors were calculated as follows: The mean total indel rates were first calculated for the sample with primary and non-targeting gRNA and for the sample with primary and secondary gRNA(s) (as described above). Then the difference of these two values were calculated and then divided by the mean total indel rate of the primary and non-targeting gRNA sample, multiplied by 100 ± propagation of uncertainty of the SD for *n* = 3 biological replicates.

### Reporting summary

Further information on research design is available in the Nature Research Reporting Summary linked to this article.

## Supplementary information

Supplementary Information

Reporting Summary

Supplementary Data 1

## Data Availability

The high-throughput sequencing data generated in this study have been deposited in the NCBI Sequencing Read Archive database under Accession Number PRJNA819982. Source data is available in the Source Data file. Source data are provided with this paper.

## Source data

Source Data

## References

[1] Jinek M (2012). A programmable dual-RNA-guided DNA endonuclease in adaptive bacterial immunity. Science.

[2] Ranjha L, Howard SM, Cejka P (2018). Main steps in DNA double-strand break repair: an introduction to homologous recombination and related processes. Chromosoma.

[3] Mali P (2013). RNA-guided human genome engineering via Cas9. Science.

[4] Cong L (2013). Multiplex genome engineering Using CRISPR/Cas systems. Science.

[5] Jinek M (2013). RNA-programmed genome editing in human cells. eLife.

[6] Cho SW, Kim S, Kim JM, Kim J-S (2013). Targeted genome engineering in human cells with the Cas9 RNA-guided endonuclease. Nat. Biotechnol..

[7] Yeh CD, Richardson CD, Corn JE (2019). Advances in genome editing through control of DNA repair pathways. Nat. Cell Biol..

[8] Liu M (2019). Methodologies for improving HDR efficiency. Front. Genet..

[9] Liang X, Potter J, Kumar S, Ravinder N, Chesnut JD (2017). Enhanced CRISPR/Cas9-mediated precise genome editing by improved design and delivery of gRNA, Cas9 nuclease, and donor DNA. J. Biotechnol..

[10] Yang L (2013). Optimization of scarless human stem cell genome editing. Nucleic Acids Res.

[11] Richardson CD, Ray GJ, DeWitt MA, Curie GL, Corn JE (2016). Enhancing homology-directed genome editing by catalytically active and inactive CRISPR-Cas9 using asymmetric donor DNA. Nat. Biotechnol..

[12] Aird EJ, Lovendahl KN, St Martin A, Harris RS, Gordon WR (2018). Increasing Cas9-mediated homology-directed repair efficiency through covalent tethering of DNA repair template. Commun. Biol..

[13] Savic N (2018). Covalent linkage of the DNA repair template to the CRISPR-Cas9 nuclease enhances homology-directed repair. eLife.

[14] Paquet D (2016). Efficient introduction of specific homozygous and heterozygous mutations using CRISPR/Cas9. Nature.

[15] Lin S, Staahl BT, Alla RK, Doudna JA (2014). Enhanced homology-directed human genome engineering by controlled timing of CRISPR/Cas9 delivery. eLife.

[16] Wienert B (2020). Timed inhibition of CDC7 increases CRISPR-Cas9 mediated templated repair. Nat. Commun..

[17] Maruyama T (2015). Increasing the efficiency of precise genome editing with CRISPR-Cas9 by inhibition of nonhomologous end joining. Nat. Biotechnol..

[18] Riesenberg S (2019). Simultaneous precise editing of multiple genes in human cells. Nucleic Acids Res.

[19] Canny MD (2018). Inhibition of 53BP1 favors homology-dependent DNA repair and increases CRISPR–Cas9 genome-editing efficiency. Nat. Biotechnol..

[20] Charpentier M (2018). CtIP fusion to Cas9 enhances transgene integration by homology-dependent repair. Nat. Commun..

[21] Rees HA, Yeh W-H, Liu DR (2019). Development of hRad51–Cas9 nickase fusions that mediate HDR without double-stranded breaks. Nat. Commun..

[22] Komor AC, Kim YB, Packer MS, Zuris JA, Liu DR (2016). Programmable editing of a target base in genomic DNA without double-stranded DNA cleavage. Nature.

[23] Gaudelli NM (2017). Programmable base editing of A•T to G•C in genomic DNA without DNA cleavage. Nature.

[24] Anzalone AV (2019). Search-and-replace genome editing without double-strand breaks or donor DNA. Nature.

[25] Roberts B (2017). Systematic gene tagging using CRISPR/Cas9 in human stem cells to illuminate cell organization. Mol. Biol. Cell.

[26] Koch B (2018). Generation and validation of homozygous fluorescent knock-in cells using CRISPR–Cas9 genome editing. Nat. Protoc..

[27] Lackner DH (2015). A generic strategy for CRISPR-Cas9-mediated gene tagging. Nat. Commun..

[28] Gantz VM, Bier E (2015). The mutagenic chain reaction: A method for converting heterozygous to homozygous mutations. Science.

[29] Shou J, Li J, Liu Y, Wu Q (2018). Precise and Predictable CRISPR Chromosomal Rearrangements Reveal Principles of Cas9-Mediated Nucleotide Insertion. Mol. Cell.

[30] Wienert B (2019). Unbiased detection of CRISPR off-targets in vivo using DISCOVER-Seq. Science.

[31] Bae S, Kweon J, Kim HS, Kim J-S (2014). Microhomology-based choice of Cas9 nuclease target sites. Nat. Methods.

[32] Shen MW (2018). Predictable and precise template-free CRISPR editing of pathogenic variants. Nature.

[33] Kosicki M, Tomberg K, Bradley A (2018). Repair of double-strand breaks induced by CRISPR–Cas9 leads to large deletions and complex rearrangements. Nat. Biotechnol..

[34] Leibowitz ML (2021). Chromothripsis as an on-target consequence of CRISPR–Cas9 genome editing. Nat. Genet..

[35] Bylund L, Kytölä S, Lui W-O, Larsson C, Weber G (2004). Analysis of the cytogenetic stability of the human embryonal kidney cell line 293 by cytogenetic and STR profiling approaches. Cytogenet. Genome Res..

[36] Lin Y-C (2014). Genome dynamics of the human embryonic kidney 293 lineage in response to cell biology manipulations. Nat. Commun..

[37] Doench JG (2014). Rational design of highly active sgRNAs for CRISPR-Cas9–mediated gene inactivation. Nat. Biotechnol..

[38] Kuscu C (2017). CRISPR-STOP: gene silencing through base-editing-induced nonsense mutations. Nat. Methods.

[39] Aguirre AJ (2016). Genomic Copy Number Dictates a Gene-Independent Cell Response to CRISPR/Cas9 Targeting. Cancer Disco..

[40] Ihry RJ (2018). p53 inhibits CRISPR–Cas9 engineering in human pluripotent stem cells. Nat. Med..

[41] Haapaniemi E, Botla S, Persson J, Schmierer B, Taipale J (2018). CRISPR–Cas9 genome editing induces a p53-mediated DNA damage response. Nat. Med..

[42] Bae S, Park J, Kim J-S (2014). Cas-OFFinder: a fast and versatile algorithm that searches for potential off-target sites of Cas9 RNA-guided endonucleases. Bioinformatics.

[43] Tsai SQ (2015). GUIDE-seq enables genome-wide profiling of off-target cleavage by CRISPR-Cas nucleases. Nat. Biotechnol..

[44] Hsu PD (2013). DNA targeting specificity of RNA-guided Cas9 nucleases. Nat. Biotechnol..

[45] Kim D (2015). Digenome-seq: genome-wide profiling of CRISPR-Cas9 off-target effects in human cells. Nat. Methods.

[46] Kulcsár PI (2017). Crossing enhanced and high fidelity SpCas9 nucleases to optimize specificity and cleavage. Genome Biol..

[47] Kulcsár PI (2020). Blackjack mutations improve the on-target activities of increased fidelity variants of SpCas9 with 5′G-extended sgRNAs. Nat. Commun..

[48] Slaymaker IM (2016). Rationally engineered Cas9 nucleases with improved specificity. Science.

[49] Kleinstiver BP (2016). High-fidelity CRISPR–Cas9 nucleases with no detectable genome-wide off-target effects. Nature.

[50] Chen JS (2017). Enhanced proofreading governs CRISPR–Cas9 targeting accuracy. Nature.

[51] Casini A (2018). A highly specific SpCas9 variant is identified by in vivo screening in yeast. Nat. Biotechnol..

[52] Bishop, A. L. et al. Double-tap gene drive uses iterative genome targeting to help overcome resistance alleles. 10.1038/s41467-022-29868-3. 10.1038/s41467-022-29868-3 PMC9085836 35534475

[53] Bitinaite J (2007). USER friendly DNA engineering and cloning method by uracil excision. Nucleic Acids Res.

[54] Clement K (2019). CRISPResso2 provides accurate and rapid genome editing sequence analysis. Nat. Biotechnol..

[55] Wickham, H. *Ggplot2: elegant graphics for data analysis*. (Springer, 2009).

