# Supplementary material for: Targeting double-strand break indel byproducts with secondary guide RNAs improves Cas9 HDR-mediated genome editing efficiencies
Source: Nat Commun. 2022 May 9;13:2351. doi: 10.1038/s41467-022-29989-9 (PMC9085776; doi:10.1038/s41467-022-29989-9)
Supplement: Supplementary file 1 — Supplementary Information [file 41467_2022_29989_MOESM1_ESM.pdf]

Supplementary information for

Targeting double-strand break indel byproducts with secondary guide RNAs improves Cas9 HDR-mediated genome editing efficiencies

Zsolt Bodai, Alena L. Bishop, Valentino Gantz, Alexis C. Komor

**Supplementary Figure 1**	InDelphi predictions and experimentally determined indel sequences for all genomic loci studied in this work.
**Supplementary Figure 2**	Improvements in HDR-mediated genome editing with ssODNs using the double tap method at the *HEK2* site.
**Supplementary Figure 3**	Improvements in HDR to NHEJ ratios using the double tap method.
**Supplementary Figure 4**	Combined improvements in HDR-mediated genome editing using the double tap method and ssODN blocking mutations at the *FANCF* (in which a low-frequency indel was targeted), *APOB1* and *MMACHC* sites.
**Supplementary Figure 5**	Morphology changes of HEK293T cells after dimethyl sulfoxide (DMSO) and Alt-R ™ HDR Enhancer V2 treatment 24 hours after transfection.
**Supplementary Figure 6**	Indel frequencies for Cas9 ribonucleoprotein (RNP)-treated cells.
**Supplementary Figure 7**	Secondary and alternative secondary gRNAs for the *APOB1* site to target the most frequent indel (a 1-bp insertion product).
**Supplementary Figure 8**	Indel frequencies generated with candidate primary gRNAs at the *HBB* and *HEXA* loci.
**Supplementary Figure 9**	Secondary gRNA designs for the *HBB1*, *HBB5*, *HEXA2* and *HEXA5* primary gRNAs.
**Supplementary Figure 10**	Alternative secondary gRNA designs at the *RNF2* site to avoid unwanted off-target editing.
**Supplementary Figure 11**	Sequences of primary and secondary protospacers and PAMs, and their respective off-target sites that we evaluated for the *APOB1*, *MMACHC* and *HBB5* sites.
**Supplementary Figure 12**	Assessment of the effect of secondary gRNAs on cell viability.
**Supplementary Figure 13**	The design of secondary gRNAs when indels with small deletions (likely facilitated by MMEJ) are targeted can result in a secondary gRNA that targets the original DNA sequence, but with an undesired alternate cut site.
**Supplementary Figure 14**	Example of flow cytometry and FACS gating.

ACTB

InDelphi

Summary of predictions at target site with gRNA: GCTATTCTCGCAGCTCACCA

<table>
<tr><th>Alignment</th><th>Category</th><th>%</th></tr>
<tr><td>GCGCGCCCGGCTATTCTCGCAGCTCA|CCATGGATGATGATATCGCCGCGCTC</td><td>Reference</td><td>-</td></tr>
<tr><td>GCGCGCCCGGCTATTCTCGCAGCTCA|---TGGATGATGATATCGCCGCGCTC</td><td>3-bp deletion</td><td>22.5</td></tr>
<tr><td>GCGCGCCCGGCTATTCTCGCAGCTCAACCATGGATGATGATATCGCCGCGCTC</td><td>1-bp insertion</td><td>14.5</td></tr>
<tr><td>GCGCGCCCGGCTATTCTCGCA-----|---TGGATGATGATATCGCCGCGCTC</td><td>8-bp deletion</td><td>6.4</td></tr>
<tr><td>GCGCGCCCGGCTATTCTCGCAGCTC-|-CATGGATGATGATATCGCCGCGCTC</td><td>2-bp deletion</td><td>5.3</td></tr>
<tr><td>GCGCGCCCGGCTATTCTCGCAGCTCA|--ATGGATGATGATATCGCCGCGCTC</td><td>2-bp deletion</td><td>5.1</td></tr>
<tr><td>GCGCGCCCGGCTATTCTCGCAGC---|CCATGGATGATGATATCGCCGCGCTC</td><td>3-bp deletion</td><td>3.8</td></tr>
<tr><td>GCGCGCCCGGCTATTCTCGCAGCTC-|CCATGGATGATGATATCGCCGCGCTC</td><td>1-bp deletion</td><td>3.3</td></tr>
<tr><td>GCGCGCCCGGCTATTCTCGCAGCTCA|-CATGGATGATGATATCGCCGCGCTC</td><td>1-bp deletion</td><td>3.3</td></tr>
<tr><td>GCGCGCCCGGCTATTCTCGCAGC---|-CATGGATGATGATATCGCCGCGCTC</td><td>4-bp deletion</td><td>3.2</td></tr>
<tr><td>GCGCGCCCGGCTATTCTCGCAGC---|--ATGGATGATGATATCGCCGCGCTC</td><td>5-bp deletion</td><td>2.5</td></tr>
</table>

CRISPRESSO

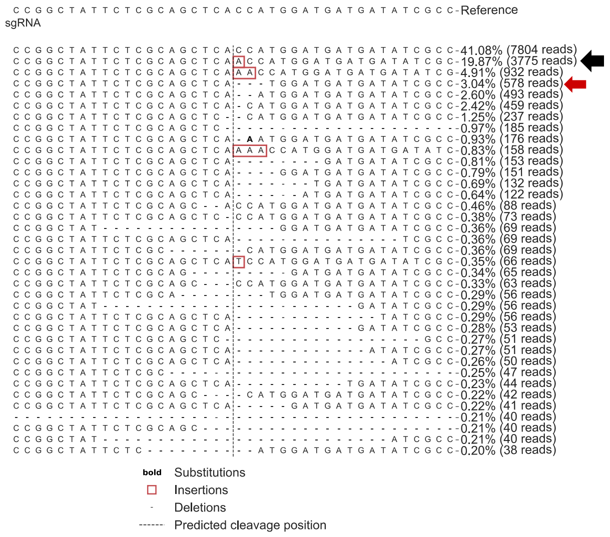

APOB1

InDelphi

HEK293T

Summary of predictions at target site with gRNA: GCCGACATCTCTATGGTGAA

<table>
<tr><th>Alignment</th><th>Category</th><th>%</th></tr>
<tr><td>TAGCCGAATGCCGACATCTCTATGGT|GAATGGAGACACTTCAACATTGACAA</td><td>Reference</td><td>-</td></tr>
<tr><td>TAGCCGAATGCCGACATCTCTATGGTTGAATGGAGACACTTCAACATTGACAA</td><td>1-bp insertion</td><td>39.4</td></tr>
<tr><td>TAGCCGAATGCCGACATCTCTATGG-|------AGACACTTCAACATTGACAA</td><td>7-bp deletion</td><td>29.1</td></tr>
<tr><td>TAGCCGAATGCCGACATCTCTATGG-|-AATGGAGACACTTCAACATTGACAA</td><td>2-bp deletion</td><td>2.7</td></tr>
<tr><td>TAGCCGAATGCCGACATCTCTATGGT|--ATGGAGACACTTCAACATTGACAA</td><td>2-bp deletion</td><td>2.6</td></tr>
<tr><td>TAGCCGAATGCCGACATCTCTATG--|-AATGGAGACACTTCAACATTGACAA</td><td>3-bp deletion</td><td>2.1</td></tr>
<tr><td>TAGCCGAATGCCGACATCTCTATGG-|GAATGGAGACACTTCAACATTGACAA</td><td>1-bp deletion</td><td>1.7</td></tr>
<tr><td>TAGCCGAATGCCGACATCTCTATGGT|-AATGGAGACACTTCAACATTGACAA</td><td>1-bp deletion</td><td>1.7</td></tr>
<tr><td>TAGCCGAATGCCGACATCTCTATGGT|----GGAGACACTTCAACATTGACAA</td><td>4-bp deletion</td><td>1.5</td></tr>
<tr><td>TAGCCGAATG----------------|-----GAGACACTTCAACATTGACAA</td><td>21-bp deletion</td><td>1.5</td></tr>
<tr><td>TAGCCGAATGCCGACATCTCTATGGTAGAATGGAGACACTTCAACATTGACAA</td><td>1-bp insertion</td><td>1.3</td></tr>
</table>

K562

Summary of predictions at target site with gRNA: GCCGACATCTCTATGGTGAA

<table>
<tr><th>Alignment</th><th>Category</th><th>%</th></tr>
<tr><td>TAGCCGAATGCCGACATCTCTATGGT|GAATGGAGACACTTCAACATTGACAA</td><td>Reference</td><td>-</td></tr>
<tr><td>TAGCCGAATGCCGACATCTCTATGGTTGAATGGAGACACTTCAACATTGACAA</td><td>1-bp insertion</td><td>33.0</td></tr>
<tr><td>TAGCCGAATGCCGACATCTCTATGG-|------AGACACTTCAACATTGACAA</td><td>7-bp deletion</td><td>32.8</td></tr>
<tr><td>TAGCCGAATGCCGACATCTCTATGG-|-AATGGAGACACTTCAACATTGACAA</td><td>2-bp deletion</td><td>3.0</td></tr>
<tr><td>TAGCCGAATGCCGACATCTCTATGGT|--ATGGAGACACTTCAACATTGACAA</td><td>2-bp deletion</td><td>3.0</td></tr>
<tr><td>TAGCCGAATGCCGACATCTCTATG--|-AATGGAGACACTTCAACATTGACAA</td><td>3-bp deletion</td><td>2.4</td></tr>
<tr><td>TAGCCGAATGCCGACATCTCTATGG-|GAATGGAGACACTTCAACATTGACAA</td><td>1-bp deletion</td><td>1.9</td></tr>
<tr><td>TAGCCGAATGCCGACATCTCTATGGT|-AATGGAGACACTTCAACATTGACAA</td><td>1-bp deletion</td><td>1.9</td></tr>
<tr><td>TAGCCGAATGCCGACATCTCTATGGT|----GGAGACACTTCAACATTGACAA</td><td>4-bp deletion</td><td>1.7</td></tr>
<tr><td>TAGCCGAATG----------------|-----GAGACACTTCAACATTGACAA</td><td>21-bp deletion</td><td>1.7</td></tr>
<tr><td>TAGCCGAATGCCGACATCTCTATGG-|-----GAGACACTTCAACATTGACAA</td><td>6-bp deletion</td><td>1.1</td></tr>
</table>

CRISPRESSO

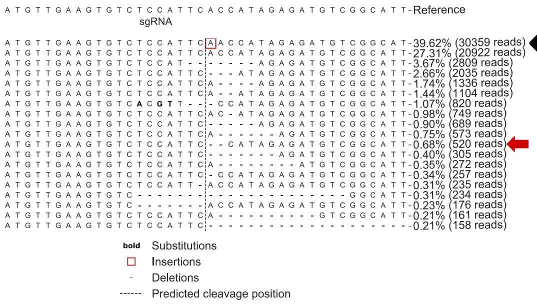

APOB2

InDelphi

Summary of predictions at target site with gRNA: GGAACTCTCGAGATGGCAGA

Alignment	Category	%
TAGGGACTTGGAACTCTCGAGATGGC|AGATGGAATCCCACAGACTTGAAGTG	Reference	-
TAGGGACTTGGAACTCTCGAGATGG-|------AATCCCACAGACTTGAAGTG	7-bp deletion	69.6
TAGGGACTTGGAACTCTCGAGATGGCCAGATGGAATCCCACAGACTTGAAGTG	1-bp insertion	7.3
TAGGGACTTGGAACTCTCGAGATGGCTAGATGGAATCCCACAGACTTGAAGTG	1-bp insertion	2.1
TAGGGACTTGGAACTCTCGAGATGGCAAGATGGAATCCCACAGACTTGAAGTG	1-bp insertion	1.7
TAGGGACTTGGAACTCTCGA------|---TGGAATCCCACAGACTTGAAGTG	9-bp deletion	1.5
TAGGGACTTGGAACTCTCGAGATGG-|--ATGGAATCCCACAGACTTGAAGTG	3-bp deletion	1.2
TAGGGACTTGGAACTCTCGAGATGGCGAGATGGAATCCCACAGACTTGAAGTG	1-bp insertion	1.0
TAGGGACTTGGAACTCTCGAGATGG-|AGATGGAATCCCACAGACTTGAAGTG	1-bp deletion	1.0
TAGGGACTTGGAACTCTCGAGATGGC|-GATGGAATCCCACAGACTTGAAGTG	1-bp deletion	1.0
TAGGGACTTGGAACTCTCGAGATG--|--ATGGAATCCCACAGACTTGAAGTG	4-bp deletion	1.0

CRISPRESSO

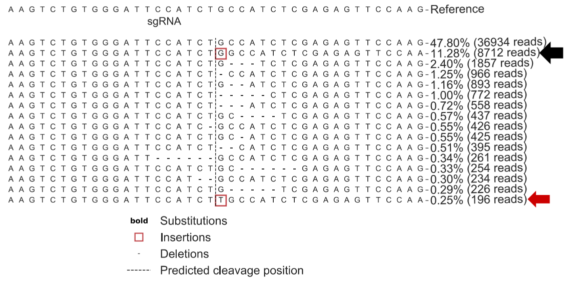

FANCF

InDelphi

Summary of predictions at target site with gRNA: GGAATCCCTTCTGCAGCACC

Alignment	Category	%
GCACCTCATGGAATCCCTTCTGCAGC|ACCTGGATCGCTTTTCCGAGCTTCTG	Reference	-
GCACCTCATGGAATCCCTTCTGCAGCCACCTGGATCGCTTTTCCGAGCTTCTG	1-bp insertion	17.3
GCACCTCATGGAATCCCTTCTG----|-----GATCGCTTTTCCGAGCTTCTG	9-bp deletion	13.3
GCACCTCATGGAATCCCT--------|----GGATCGCTTTTCCGAGCTTCTG	12-bp deletion	6.3
GCACCTCATGGAATCCCTTCTGCAGCTACCTGGATCGCTTTTCCGAGCTTCTG	1-bp insertion	5.0
GCACCTCATGGAATCCCTTCTGCAGCAACCTGGATCGCTTTTCCGAGCTTCTG	1-bp insertion	4.1
GCACCTCATGGAATCCCTTCTGCAGC|--CTGGATCGCTTTTCCGAGCTTCTG	2-bp deletion	3.9
GCACCTCATGGAATCCCTTCTGCA--|ACCTGGATCGCTTTTCCGAGCTTCTG	2-bp deletion	3.8
GCACCTCATGGAATCCCTTCTGCAGC|-----------TTTTCCGAGCTTCTG	11-bp deletion	3.1
GCACCTCATGGAATCCCTTCTGCAGC|---TGGATCGCTTTTCCGAGCTTCTG	3-bp deletion	3.0
GCACCTCATGGAATCCCTTCTGCA--|-CCTGGATCGCTTTTCCGAGCTTCTG	3-bp deletion	2.8

CRISPRESSO

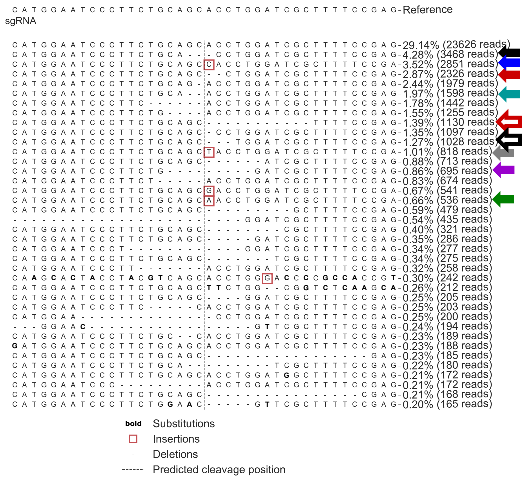

HBB1

InDelphi

Summary of predictions at target site with gRNA: CATGGTGCACCTGACTCCTG

Alignment	Category	%
AACAGACACCATGGTGCACCTGACTC|CTGAGGAGAAGTCTGCCGTTACTGCC	Reference	-
AACAGACACCATGGTGCACCTGA---|----GGAGAAGTCTGCCGTTACTGCC	7-bp deletion	40.4
AACAGACACCATGGTGCACCTGACT-|--GAGGAGAAGTCTGCCGTTACTGCC	3-bp deletion	15.9
AACAGACACCATGGTGCACCTGACTC|-TGAGGAGAAGTCTGCCGTTACTGCC	1-bp deletion	9.5
AACAGACACCATGGTGCACCTGACTCCCTGAGGAGAAGTCTGCCGTTACTGCC	1-bp insertion	4.1
AACAGACACCATGGTGCACCTGA---|-------GAAGTCTGCCGTTACTGCC	10-bp deletion	2.8
AACAGACACCATGGTGCACCTGACTC|---AGGAGAAGTCTGCCGTTACTGCC	3-bp deletion	2.7
AACAGACACCATGGTGCACCTGA---|---------AGTCTGCCGTTACTGCC	12-bp deletion	1.7
AACAGACACCATGGTG----------|---AGGAGAAGTCTGCCGTTACTGCC	13-bp deletion	1.3
AACAGACACCATGGTGCACCTGACTC|-------------TGCCGTTACTGCC	13-bp deletion	1.3
AACAGACACCATGGTGCACCTGACT-|-TGAGGAGAAGTCTGCCGTTACTGCC	2-bp deletion	1.2

CRISPRESSO

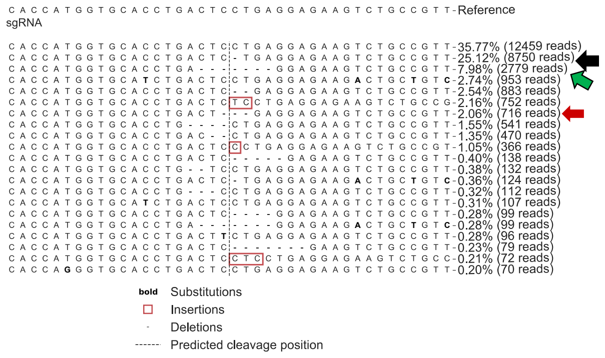

HBB2

InDelphi

Summary of predictions at target site with gRNA: ACTTCTCCTCAGGAGTCAGG

Alignment	Category	%
TAACGGCAGACTTCTCCTCAGGAGTC|AGGTGCACCATGGTGTCTGTTTGAGG	Reference	-
TAACGGCAGACTTCTCCTCAGG----|---TGCACCATGGTGTCTGTTTGAGG	7-bp deletion	21.5
TAACGGCAGACTTCTCCTCAGGAGTCCAGGTGCACCATGGTGTCTGTTTGAGG	1-bp insertion	12.8
TAACGGCAGACTTCTCCTCAGGAG--|--GTGCACCATGGTGTCTGTTTGAGG	4-bp deletion	12.7
TAACGGCAGACTTCTCCTCAGGAGT-|----GCACCATGGTGTCTGTTTGAGG	5-bp deletion	9.9
TAACGGCAGACTTCTCCTCAGGAGTCTAGGTGCACCATGGTGTCTGTTTGAGG	1-bp insertion	3.7
TAACGGCAGACTTCTCCTCAGGAGTCAAGGTGCACCATGGTGTCTGTTTGAGG	1-bp insertion	3.0
TAACGGCAGACTTCTCCTCAGGAGT-|AGGTGCACCATGGTGTCTGTTTGAGG	1-bp deletion	2.4
TAACGGCAGACTTCTCCTCAGGAGTC|-GGTGCACCATGGTGTCTGTTTGAGG	1-bp deletion	2.4
TAACGGCAGACTTCTCCTCAGGAGTCGAGGTGCACCATGGTGTCTGTTTGAGG	1-bp insertion	1.8
TAACGGCAGACTTCTCCTCAGGAGTC|-----------------TGTTTGAGG	17-bp deletion	1.8

CRISPRESSO

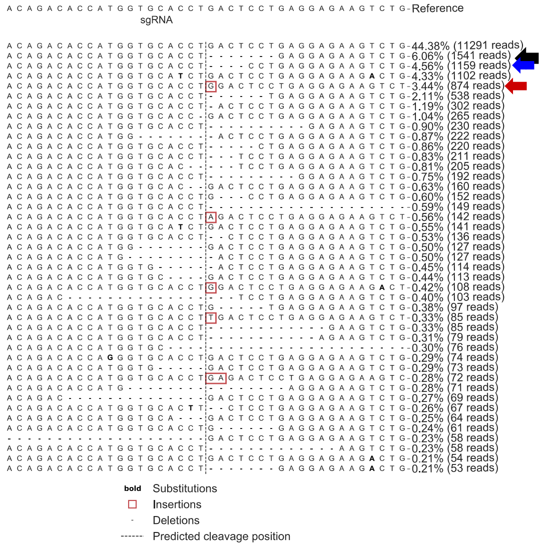

HBB3

InDelphi

Summary of predictions at target site with gRNA: GTAACGGCAGACTTCTCCTC

```
Alignment                                                      Category          %
CACAGGGCAGTAACGGCAGACTTCTC|CTCAGGAGTCAGGTGCACCATGGTGT         Reference         -
CACAGGGCAGTAACGGCAGACTTCTC|---AGGAGTCAGGTGCACCATGGTGT         3-bp deletion     47.9
CACAGGGCAGTAACGGCAGACTTC--|---AGGAGTCAGGTGCACCATGGTGT         5-bp deletion     7.7
CACAGGGCAGTAACGGCAGACTTCTC|-TCAGGAGTCAGGTGCACCATGGTGT         1-bp deletion     7.6
CACAGGGCAGTAACGGCAGACT----|--CAGGAGTCAGGTGCACCATGGTGT         6-bp deletion     6.0
CACAGGGCAGTAACGGCAG-------|-----GAGTCAGGTGCACCATGGTGT         12-bp deletion    4.8
CACAGGGCAGTAACGGCAGACTTCTCCCTCAGGAGTCAGGTGCACCATGGTGT         1-bp insertion    4.1
CACAGGGCAGTAACGGCAGACTTCTC|----------AGGTGCACCATGGTGT         10-bp deletion    2.2
CACAGGGCAGTAACGGCAGACTTC--|----------AGGTGCACCATGGTGT         12-bp deletion    1.3
CACAGGGCAGTAACGGCAGACTTCTCTCTCAGGAGTCAGGTGCACCATGGTGT         1-bp insertion    1.2
CACAGGGCAGTAACGGCAGA------|-------GTCAGGTGCACCATGGTGT         13-bp deletion    1.0
```

CRISPRESSO

```
TGGTGCATCTGACTCCTGAGGAGAAGTCTGCCGTTACTGC-Reference
sgRNA

TGGTGCACCTGACTCCTGAG|GAGAAGTCTGCCGTTACTGC-34.87% (19581 reads)
TGGTGCACCTGACTCCTGAG|-AGAAGTCTGCCGTTACTGC-20.88% (11724 reads)
TGGTGCACCTGACTCCTGAG|---AAGTCTGCCGTTACTGC-14.60% (8196 reads)
TGGTGCATCTGACTCCTGAG|GAGAAGACTGCTGTCAATGC-3.92% (2201 reads)
TGGTGCACCTGACTCCTG--|GAGAAGTCTGCCGTTACTGC-2.46% (1381 reads)
TGGTGCACCTGACTCCTGAG|--GAAGTCTGCCGTTACTGC-1.66% (934 reads)
TGGTGCACCTGACTCCTGAG|------TCTGCCGTTACTGC-1.18% (661 reads)
TGGTGCACCTGACTCCTG--|----------CCGTTACTGC-1.03% (580 reads)
TGGTGCACCTGACTCCTGAG|GGAGAAGTCTGCCGTTACTG-0.99% (554 reads)
TGGTGCACCTGACTCCTGA-|----AGTCTGCCGTTACTGC-0.74% (415 reads)
TGGTGCACCTGACTCCTGAG|-----GTCTGCCGTTACTGC-0.72% (402 reads)
TGGTGCACCTGACTCCTGAG|GAGAAGACTGCTGTCAATGC-0.63% (353 reads)
TGGTGCACCTGACTCCTGAG|GAGAGAAGTCTGCCGTTACT-0.59% (330 reads)
TGGTGCACCTGACTCCTGAG|----AGTCTGCCGTTACTGC-0.47% (263 reads)
TGGTGCACCTGACTC-----|GAGAAGTCTGCCGTTACTGC-0.41% (231 reads)
TGGTGCACCTG---------|GAGAAGTCTGCCGTTACTGC-0.27% (151 reads)
TGGTGCACCTG---------|----------CCGTTACTGC-0.25% (140 reads)
TGGTGCACCTGA--------|--GAAGTCTGCCGTTACTGC-0.23% (128 reads)
TGGTGCACCTGACTCC----|GAGAAGTCTGCCGTTACTGC-0.22% (125 reads)
TGGTGCACCTGA--------|GAGAAGTCTGCCGTTACTGC-0.20% (114 reads)
```

bold Substitutions
□ Insertions
\- Deletions
------ Predicted cleavage position

HBB4

InDelphi

Summary of predictions at target site with gRNA: CTGACTCCTGAGGAGAAGTC

```
Alignment                                                      Category          %
ATGGTGCACCTGACTCCTGAGGAGAA|GTCTGCCGTTACTGCCCTGTGGGGCA         Reference         -
ATGGTGCACCTGACTCCTGAGGAGAAAGTCTGCCGTTACTGCCCTGTGGGGCA         1-bp insertion    30.6
ATGGTGCACCTGACTCCTG-------|-----CCGTTACTGCCCTGTGGGGCA         12-bp deletion    9.3
ATGGTGCACCTGACTCCTGAGGAG--|-TCTGCCGTTACTGCCCTGTGGGGCA         3-bp deletion     4.5
ATGGTGCACCTGACTCCTGAGGAGAA|-TCTGCCGTTACTGCCCTGTGGGGCA         1-bp deletion     3.6
ATGGTGCACCTGACTCCTGAGGAGA-|GTCTGCCGTTACTGCCCTGTGGGGCA         1-bp deletion     3.6
ATGGTGCACCTGACTCCTGAGGAGAATGTCTGCCGTTACTGCCCTGTGGGGCA         1-bp insertion    3.1
ATGGTGCACCTGACTCCTGAGG----|-TCTGCCGTTACTGCCCTGTGGGGCA         5-bp deletion     2.7
ATGGTGCACCTGACTCCTGAG-----|-TCTGCCGTTACTGCCCTGTGGGGCA         6-bp deletion     2.1
ATGGTGCACCTGACTCCTGAGGAGAA|---TGCCGTTACTGCCCTGTGGGGCA         3-bp deletion     2.1
ATGGTGCACCTGACTCCTGAGGAGA-|--CTGCCGTTACTGCCCTGTGGGGCA         3-bp deletion     2.1
```

CRISPRESSO

```
CATCTGACTCCTGAGGAGAAGTCTGCCGTTACTGCCCTGT-Reference
sgRNA

CACCTGACTCCTGAGGAGAA|GTCTGCCGTTACTGCCCTGT-87.37% (62776 reads)
CATCTGACTCCTGAGGAGAA|GACTGCTGTCAATGCCCTGT-2.99% (2145 reads)
CACCTGACTCCTGAGGAGAA|AGTCTGCCGTTACTGCCCTG-1.26% (905 reads)
CACCTGACTCCTGAGGAGA-|GTCTGCCGTTACTGCCCTGT-0.73% (522 reads)
CACCTGACTCCTGAGGAGAA|GACTGCTGTCAATGCCCTGT-0.47% (341 reads)
CACCTGACTCCTGAGGAG--|-TCTGCCGTTACTGCCCTGT-0.44% (313 reads)
```

bold Substitutions
□ Insertions
\- Deletions
------ Predicted cleavage position

HBB5

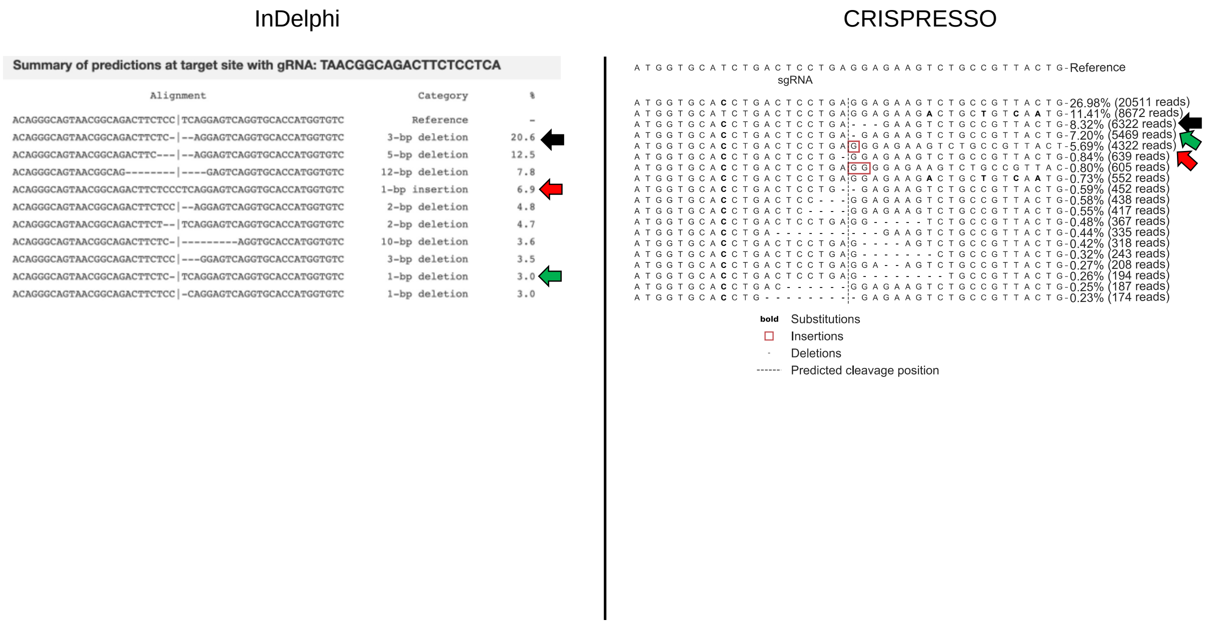
InDelphi
CRISPRESSO
Summary of predictions at target site with gRNA: TAACGGCAGACTTCTCCTCA
Alignment
Category
%
ACAGGGCAGTAACGGCAGACTTCTCC|TCAGGAGTCAGGTGCACCATGGTGTC Reference -
ACAGGGCAGTAACGGCAGACTTCTC-|--AGGAGTCAGGTGCACCATGGTGTC 3-bp deletion 20.6
ACAGGGCAGTAACGGCAGACTTC---|--AGGAGTCAGGTGCACCATGGTGTC 5-bp deletion 12.5
ACAGGGCAGTAACGGCAG--------|----GAGTCAGGTGCACCATGGTGTC 12-bp deletion 7.8
ACAGGGCAGTAACGGCAGACTTCTCCCTCAGGAGTCAGGTGCACCATGGTGTC 1-bp insertion 6.9
ACAGGGCAGTAACGGCAGACTTCTCC|--AGGAGTCAGGTGCACCATGGTGTC 2-bp deletion 4.8
ACAGGGCAGTAACGGCAGACTTCT--|TCAGGAGTCAGGTGCACCATGGTGTC 2-bp deletion 4.7
ACAGGGCAGTAACGGCAGACTTCTC-|---------AGGTGCACCATGGTGTC 10-bp deletion 3.6
ACAGGGCAGTAACGGCAGACTTCTCC|---GGAGTCAGGTGCACCATGGTGTC 3-bp deletion 3.5
ACAGGGCAGTAACGGCAGACTTCTC-|TCAGGAGTCAGGTGCACCATGGTGTC 1-bp deletion 3.0
ACAGGGCAGTAACGGCAGACTTCTCC|-CAGGAGTCAGGTGCACCATGGTGTC 1-bp deletion 3.0
Reference
sgRNA
26.98% (20511 reads)
11.41% (8672 reads)
8.32% (6322 reads)
7.20% (5469 reads)
5.69% (4322 reads)
0.84% (639 reads)
0.80% (605 reads)
0.73% (552 reads)
0.59% (452 reads)
0.58% (438 reads)
0.55% (417 reads)
0.48% (367 reads)
0.44% (335 reads)
0.42% (318 reads)
0.32% (243 reads)
0.27% (208 reads)
0.26% (194 reads)
0.25% (187 reads)
0.23% (174 reads)
bold Substitutions
Insertions
Deletions
Predicted cleavage position

HEK2

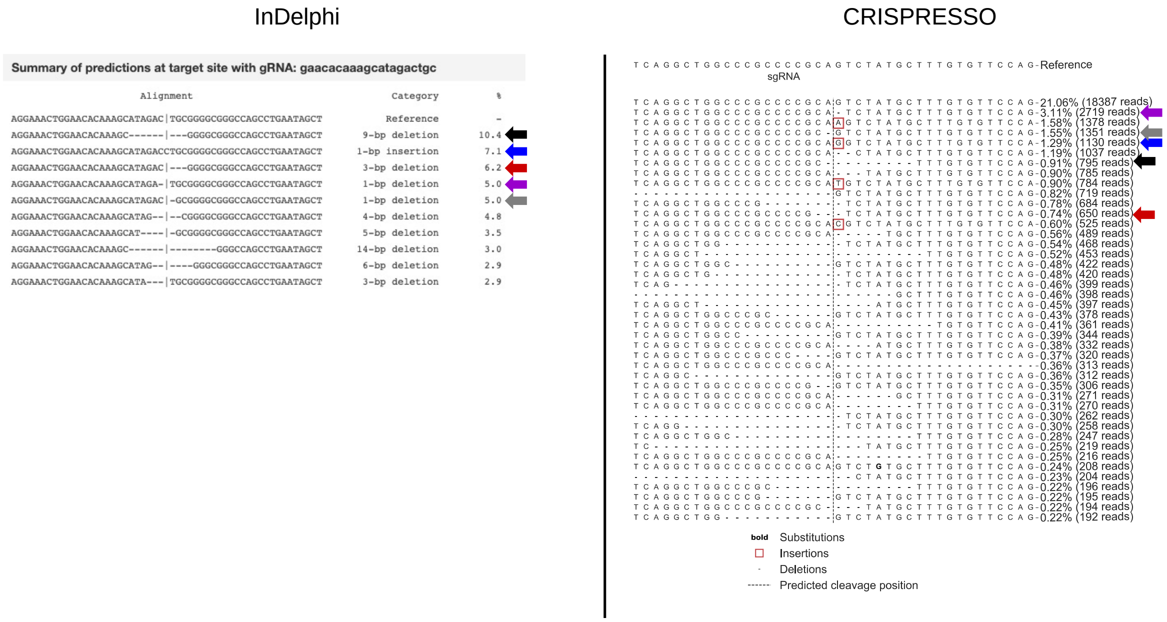
InDelphi
CRISPRESSO
Summary of predictions at target site with gRNA: gaacacaaagcatagactgc
Alignment
Category
%
AGGAAACTGGAACACAAAGCATAGAC|TGCGGGGCGGGCCAGCCTGAATAGCT Reference -
AGGAAACTGGAACACAAAGC------|---GGGGCGGGCCAGCCTGAATAGCT 9-bp deletion 10.4
AGGAAACTGGAACACAAAGCATAGACCTGCGGGGCGGGCCAGCCTGAATAGCT 1-bp insertion 7.1
AGGAAACTGGAACACAAAGCATAGAC|---GGGGCGGGCCAGCCTGAATAGCT 3-bp deletion 6.2
AGGAAACTGGAACACAAAGCATAGA-|TGCGGGGCGGGCCAGCCTGAATAGCT 1-bp deletion 5.0
AGGAAACTGGAACACAAAGCATAGAC|-GCGGGGCGGGCCAGCCTGAATAGCT 1-bp deletion 5.0
AGGAAACTGGAACACAAAGCATAG--|--CGGGGCGGGCCAGCCTGAATAGCT 4-bp deletion 4.8
AGGAAACTGGAACACAAAGCAT----|-GCGGGGCGGGCCAGCCTGAATAGCT 5-bp deletion 3.5
AGGAAACTGGAACACAAAGC------|--------GGGCCAGCCTGAATAGCT 14-bp deletion 3.0
AGGAAACTGGAACACAAAGCATAG--|----GGGCGGGCCAGCCTGAATAGCT 6-bp deletion 2.9
AGGAAACTGGAACACAAAGCATA---|TGCGGGGCGGGCCAGCCTGAATAGCT 3-bp deletion 2.9
Reference
sgRNA
bold Substitutions
Insertions
Deletions
Predicted cleavage position

HEK3

InDelphi

Summary of predictions at target site with gRNA: GGCCCAGACTGAGCACGTGA

<table>
<tr><th>Alignment</th><th>Category</th><th>%</th></tr>
<tr><td>AATCCTTGGGGCCCAGACTGAGCACG|TGATGGCAGAGGAAAGGAAGCCCTGC</td><td>Reference</td><td>-</td></tr>
<tr><td>AATCCTTGGGGCCCAGACTGA-----|---TGGCAGAGGAAAGGAAGCCCTGC</td><td>8-bp deletion</td><td>16.7</td></tr>
<tr><td>AATCCTTGGGGCCCAGACTGAGCA--|--------GAGGAAAGGAAGCCCTGC</td><td>10-bp deletion</td><td>13.8</td></tr>
<tr><td>AATCCTTGGGGCCCAGACTGAGCACG|--ATGGCAGAGGAAAGGAAGCCCTGC</td><td>2-bp deletion</td><td>5.2</td></tr>
<tr><td>AATCCTTGGGGCCCAGACTGAGCA--|TGATGGCAGAGGAAAGGAAGCCCTGC</td><td>2-bp deletion</td><td>5.1</td></tr>
<tr><td>AATCCTTGGGGCCCAGACTGAG----|-----------GAAAGGAAGCCCTGC</td><td>15-bp deletion</td><td>3.9</td></tr>
<tr><td>AATCCTTGGGGCCCAGACTGAGCACG|-GATGGCAGAGGAAAGGAAGCCCTGC</td><td>1-bp deletion</td><td>3.3</td></tr>
<tr><td>AATCCTTGGGGCCCAGACTGAGCAC-|TGATGGCAGAGGAAAGGAAGCCCTGC</td><td>1-bp deletion</td><td>3.3</td></tr>
<tr><td>AATCCTTGGGGCCCAGACTGAGCACGGTGATGGCAGAGGAAAGGAAGCCCTGC</td><td>1-bp insertion</td><td>3.1</td></tr>
<tr><td>AATCCTTGGGGCCCAGACTG------|-----GCAGAGGAAAGGAAGCCCTGC</td><td>11-bp deletion</td><td>3.0</td></tr>
<tr><td>AATCCTTGGGGCCCAGA---------|----------GGAAAGGAAGCCCTGC</td><td>19-bp deletion</td><td>2.8</td></tr>
</table>

CRISPRESSO

mutation in cell line

Sequence	% (reads)
TGGGGCCCAGACTGAGCACGTGATGGCAGAGGAAAGGAAG	Reference
sgRNA	
TGGGGCCCAGACTGAGCACGTGATGGCAGAGGAAAGGAAG	19.05% (11781 reads)
TGGGGCCCAGACTGAGCACGTGATGGCAAAGGAAAGGAAG	8.58% (5304 reads)
TGGGGCCCAGACTGAGCA--TGATGGCAGAGGAAAGGAAG	8.05% (4979 reads)
TGGGGCCCAGACTGAGCAC-TGATGGCAGAGGAAAGGAAG	6.86% (4240 reads)
TGGGGCCCAGACTGAGCA--TGATGGCAAAGGAAAGGAAG	4.29% (2655 reads)
TGGGGCCCAGACTGAGCACG--ATGGCAGAGGAAAGGAAG	3.28% (2031 reads)
TGGGGCCCAGACTGAGCAC-TGATGGCAAAGGAAAGGAAG	3.04% (1881 reads)
TGGGGCCCAGACTGAGCACGGTGATGGCAGAGGAAAGGAA	2.39% (1478 reads)
TGGGGCCCAGACTGA--------TGGCAGAGGAAAGGAAG	1.99% (1230 reads)
TGGGGCCCAGACTGAGCACG--ATGGCAAAGGAAAGGAAG	1.82% (1128 reads)
TGGGGCCCAGACTGAGCA----------GAGGAAAGGAAG	1.47% (907 reads)
TGGGGCCCAGACTGAGCACGGTGATGGCAAAGGAAAGGAA	1.34% (826 reads)
TGGGGCCCAGACTGAGCACGTTGATGGCAGAGGAAAGGAA	1.31% (810 reads)
TGGGGCCCAGACTGA--------TGGCAAAGGAAAGGAAG	1.30% (802 reads)
TGGGGCCCAGACTGAGCAA----------AGGAAAGGAAG	0.96% (592 reads)
TGGGGCCCAGACTGAGCACGATGATGGCAGAGGAAAGGAA	0.61% (379 reads)
TGGGGCCCAGACTGAGCACGTTGATGGCAAAGGAAAGGAA	0.58% (356 reads)
TGGGGCCCAGACTGAGCACG------CAGAGGAAAGGAAG	0.45% (278 reads)
TGGGGCCCAGACTGAG----TGATGGCAGAGGAAAGGAAG	0.39% (239 reads)
TGGGGCCCAGACTGAGC---TGATGGCAGAGGAAAGGAAG	0.36% (221 reads)
TGGGGCCCAGACTGAGCACG------CAAAGGAAAGGAAG	0.33% (207 reads)
TGGGGCCCAGACTGAGCACG-GATGGCAAAGGAAAGGAAG	0.31% (194 reads)
TGGGGCCCAGACTGAGCAA-TGATGGCAGAGGAAAGGAAG	0.30% (188 reads)
TGGGGC--------------TGATGGCAGAGGAAAGGAAG	0.29% (182 reads)
TGGGGCCCAGACTGAGCACG-GATGGCAGAGGAAAGGAAG	0.29% (179 reads)
TGGGGCCCAGACTGAG---------------GAAAGGAAG	0.26% (161 reads)
TGGGGCCCAGACTG-----------GCAGAGGAAAGGAAG	0.25% (157 reads)
TGGGGCCCAGACTGAG----TGATGGCAAAGGAAAGGAAG	0.25% (156 reads)
TGGGGCCCAGACTGAGCACGATGATGGCAAAGGAAAGGAA	0.25% (153 reads)
TGGGGCC-------------TGATGGCAGAGGAAAGGAAG	0.24% (149 reads)
TGGGGCCCAGACTGAG---GTGATGGCAGAGGAAAGGAAG	0.23% (144 reads)
TGGGGCCCAGACTGAGC---TGATGGCAAAGGAAAGGAAG	0.21% (132 reads)

bold Substitutions
□ Insertions
\- Deletions
------ Predicted cleavage position

HEXA1

InDelphi

Summary of predictions at target site with gRNA: TACCTGAACCGTATATCCTA

<table>
<tr><th>Alignment</th><th>Category</th><th>%</th></tr>
<tr><td>GCCCCCTGGTACCTGAACCGTATATC|CTATGGCCCTGACTGGAAGGATTTCT</td><td>Reference</td><td>-</td></tr>
<tr><td>GCCCCCTGGTACCTGAACCGTATAT-|----GGCCCTGACTGGAAGGATTTCT</td><td>5-bp deletion</td><td>25.7</td></tr>
<tr><td>GCCCCCTGGTACCTGAACCGTAT---|----GGCCCTGACTGGAAGGATTTCT</td><td>7-bp deletion</td><td>15.6</td></tr>
<tr><td>GCCCCCTGGTACCTGAACCGTATATC|-TATGGCCCTGACTGGAAGGATTTCT</td><td>1-bp deletion</td><td>12.4</td></tr>
<tr><td>GCCCCCTGGTACCTGAACCGTATAT-|--ATGGCCCTGACTGGAAGGATTTCT</td><td>3-bp deletion</td><td>3.6</td></tr>
<tr><td>GCCCCCTGGTACCTGAACCGTATATCCCTATGGCCCTGACTGGAAGGATTTCT</td><td>1-bp insertion</td><td>3.1</td></tr>
<tr><td>GCCCCCTGGTACCTGA----------|------------CTGGAAGGATTTCT</td><td>22-bp deletion</td><td>3.1</td></tr>
<tr><td>GCCCCCTGGTACCTGAACCGTAT---|CTATGGCCCTGACTGGAAGGATTTCT</td><td>3-bp deletion</td><td>1.8</td></tr>
<tr><td>GCCCCCTGGTACCTGAACCGTATATC|---TGGCCCTGACTGGAAGGATTTCT</td><td>3-bp deletion</td><td>1.8</td></tr>
<tr><td>GCCCCCTGGTACCTGAACCGTATA--|CTATGGCCCTGACTGGAAGGATTTCT</td><td>2-bp deletion</td><td>1.6</td></tr>
<tr><td>GCCCCCTGGTACCTGAACCGTATAT-|-TATGGCCCTGACTGGAAGGATTTCT</td><td>2-bp deletion</td><td>1.6</td></tr>
</table>

CRISPRESSO

Sequence	% (reads)
TGGTACCTGAACCGTATATCCTATGGCCCTGACTGGAAGG	Reference
sgRNA	
TGGTACCTGAACCGTATATCCTATGGCCCTGACTGGAAGG	47.70% (22157 reads)
TGGTACCTGAACCGTATATC-TATGGCCCTGACTGGAAGG	14.64% (6801 reads)
TGGTACCTGAACCGTATAT------GGCCCTGACTGGAAGG	5.26% (2441 reads)
TGGTACCTGAACCGTAT--------GGCCCTGACTGGAAGG	2.98% (1382 reads)
TGGTACCTGAACCGTAT---CTATGGCCCTGACTGGAAGG	1.81% (839 reads)
TGGTACCTGAACCGTATA--CTATGGCCCTGACTGGAAGG	1.52% (704 reads)
TGGTACCTGAACCGTATA-CCTATGGCCCTGACTGGAAGG	1.45% (674 reads)
TGGTACCTGAACCGTATATCCCTATGGCCCTGACTGGAAG	1.40% (650 reads)
TGGTACCTGAACCGTA----CTATGGCCCTGACTGGAAGG	1.16% (539 reads)
TGGTACCTGA----------------------CTGGAAGG	0.85% (396 reads)
TGGTACCTGAACC--------TATGGCCCTGACTGGAAGG	0.80% (373 reads)
TGGTACCTGAACCGTATATCTCCTATGGCCCTGACTGGAA	0.74% (346 reads)
TGGTACCTGAACCGTATAT---ATGGCCCTGACTGGAAGG	0.69% (321 reads)
TGGTACCTGAACCGTATATC--ATGGCCCTGACTGGAAGG	0.50% (230 reads)
TGGTACCTGAACCGTAT--CCTATGGCCCTGACTGGAAGG	0.47% (216 reads)
TGGTACCTGAACCGTATATC---------TGACTGGAAGG	0.45% (208 reads)
TGGTACCTGAAC---------------------TGGAAGG	0.43% (202 reads)
TGGTACCTGAACCGTATATCTCTATGGCCCTGACTGGAAG	0.43% (202 reads)
TGGTACCTGAACCGT-----CTATGGCCCTGACTGGAAGG	0.43% (201 reads)
TGG-----------------------CCCTGACTGGAAGG	0.43% (200 reads)
TGGTACCTG----------------GCCCTGACTGGAAGG	0.39% (179 reads)
TGGTACCTGAACCGTATAT**T**CTATGGCCCTGACTGGAAGG	0.37% (171 reads)
TGGTACCTGAACCGTATATC-----GCCCTGACTGGAAGG	0.32% (148 reads)
TGGTACCTGAACCGTA---CCTATGGCCCTGACTGGAAGG	0.30% (140 reads)
TGGTACCTGAACC---------------CTGACTGGAAGG	0.28% (132 reads)
TGGTACCTGAACCGTATATC--------------------	0.24% (112 reads)
TGGTACCTGAACCGTATATC----------GACTGGAAGG	0.23% (109 reads)
TGGTACCTGAACCGTATATC-------CCTGACTGGAAGG	0.23% (108 reads)
TGGTACCTGAACCGTATATC--------CTGACTGGAAGG	0.23% (106 reads)
TGGTACCT--------------ATGGCCCTGACTGGAAGG	0.21% (99 reads)
----------------------------CTGACTGGAAGG	0.21% (96 reads)

bold Substitutions
□ Insertions
\- Deletions
------ Predicted cleavage position

HEXA2

InDelphi

Summary of predictions at target site with gRNA: GTATATCCTATGGCCCTGAC

Alignment	Category	%
ACCTGAACCGTATATCCTATGGCCCT|GACTGGAAGGATTTCTACATAGTGGA	Reference	-
ACCTGAACCGTATATCCTATGGCCCTTGACTGGAAGGATTTCTACATAGTGGA	1-bp insertion	42.8
ACCTGAACCGTATATCCTATGGCCCT|----GGAAGGATTTCTACATAGTGGA	4-bp deletion	12.3
ACCTGAACCGTATATCCTATGG----|------AAGGATTTCTACATAGTGGA	10-bp deletion	9.8
ACCTGAACCGTATATCCTATGGCCC-|GACTGGAAGGATTTCTACATAGTGGA	1-bp deletion	2.3
ACCTGAACCGTATATCCTATGGCCCT|-ACTGGAAGGATTTCTACATAGTGGA	1-bp deletion	2.3
ACCTGAACCGTATATCCTATGG----|-ACTGGAAGGATTTCTACATAGTGGA	5-bp deletion	1.8
ACCTGAACCGTATATCCTATGGCC--|---TGGAAGGATTTCTACATAGTGGA	5-bp deletion	1.8
ACCTGAACCGTATATCCT--------|----GGAAGGATTTCTACATAGTGGA	12-bp deletion	1.7
ACCTGAACCGTATATCCTATGGCCCTAGACTGGAAGGATTTCTACATAGTGGA	1-bp insertion	1.4
ACCTGAACCGTATATCCTATGG----|----------ATTTCTACATAGTGGA	14-bp deletion	1.4

CRISPRESSO

Allele	Frequency
ACCGTATATCCTATGGCCCTGACTGGAAGGATTTCTACGT	Reference
sgRNA	
ACCGTATATCCTATGGCCCTGACTGGAAGGATTTCTACGT	42.94% (20917 reads)
ACCGTATATCCTATGGCCCTTGACTGGAAGGATTTCTACG	28.79% (14023 reads)
ACCGTATATCCTATG------ACTGGAAGGATTTCTACGT	2.63% (1279 reads)
ACCGTATATCCTATGG----------AAGGATTTCTACGT	2.39% (1164 reads)
ACCGTATATCCTATGGCC-TGACTGGAAGGATTTCTACGT	1.60% (778 reads)
ACCGTATATCCTATGGC--TGACTGGAAGGATTTCTACGT	1.43% (695 reads)
ACCGTATATCCTATGGCCC-GACTGGAAGGATTTCTACGT	1.28% (623 reads)
ACCGTATATCCTATGGC------TGGAAGGATTTCTACGT	1.11% (540 reads)
ACCGTATATCCTAT-----TGACTGGAAGGATTTCTACGT	0.77% (375 reads)
ACCGTATATCCTATGGCCCTTTGACTGGAAGGATTTCTAC	0.77% (373 reads)
ACCGTATATCCTATG----TGACTGGAAGGATTTCTACGT	0.71% (347 reads)
ACCGTATATCCTATGG---TGACTGGAAGGATTTCTACGT	0.62% (304 reads)
ACCGTATATCCTATGGCCCT----GGAAGGATTTCTACGT	0.59% (289 reads)
ACCGTATATCCTATGGCCCTCTGACTGGAAGGATTTCTAC	0.47% (230 reads)
ACCGTATATCCTATGG-----ACTGGAAGGATTTCTACGT	0.43% (209 reads)
ACCGTATATCCT------------GGAAGGATTTCTACGT	0.40% (196 reads)
ACCGTATATCCTATGGCC--GACTGGAAGGATTTCTACGT	0.34% (164 reads)
ACCGTATATCCTATGGCCCT-ACTGGAAGGATTTCTACGT	0.24% (118 reads)
-------------------------GAAGGATTTCTACGT	0.23% (114 reads)

bold Substitutions
□ Insertions
\- Deletions
------ Predicted cleavage position

HEXA3

InDelphi

Summary of predictions at target site with gRNA: TATACGGTTCAGGTACCAGG

Alignment	Category	%
GCCATAGGATATACGGTTCAGGTACC|AGGGGGCAGAGAGAAGGGCCCGGAAG	Reference	-
GCCATAGGATATACGGTTCAGG----|---GGGCAGAGAGAAGGGCCCGGAAG	7-bp deletion	21.9
GCCATAGGATATACGGTTCAGGTACCCAGGGGGCAGAGAGAAGGGCCCGGAAG	1-bp insertion	13.4
GCCATAGGATATACGGTTCAGG----|----GGCAGAGAGAAGGGCCCGGAAG	8-bp deletion	6.5
GCCATAGGATATACGGTTCAGG----|-----GCAGAGAGAAGGGCCCGGAAG	9-bp deletion	5.1
GCCATAGGATATACGGTTCAGG----|------CAGAGAGAAGGGCCCGGAAG	10-bp deletion	3.9
GCCATAGGATATACGGTTCAGGTACCTAGGGGGCAGAGAGAAGGGCCCGGAAG	1-bp insertion	3.8
GCCATAGGATATACGGTTCAG-----|---------AGAGAAGGGCCCGGAAG	14-bp deletion	3.8
GCCATAGGATATACGGTTCAGGTACCAAGGGGGCAGAGAGAAGGGCCCGGAAG	1-bp insertion	3.2
GCCATAGGATATACGGTTCAGGTA--|-GGGGCAGAGAGAAGGGCCCGGAAG	3-bp deletion	2.8
GCCATAGGATATACGGTTCAGGTAC-|AGGGGGCAGAGAGAAGGGCCCGGAAG	1-bp deletion	2.5

CRISPRESSO

Allele	Frequency
GGCCCTTCTCTCTGCCCCCTGGTACCTGAACCGTATATCC	Reference
sgRNA	
GGCCCTTCTCTCTGCCCCCTGGTACCTGAACCGTATATCC	42.43% (19632 reads)
GGCCCTTCTCTCTGCCCCCT-GTACCTGAACCGTATATCC	11.41% (5279 reads)
GGCCCTTCTCTCTGCCCCCTGGGTACCTGAACCGTATATC	10.62% (4915 reads)
GGCCCTTCTCTCTGCCCCCT-------GAACCGTATATCC	7.95% (3679 reads)
GGCCCTTCTCTCTGCCCCC-GGTACCTGAACCGTATATCC	5.62% (2601 reads)
GGCCCTTCTCTCTGCCCCCTG---CCTGAACCGTATATCC	1.52% (702 reads)
GGCCCTTCTCTCTGCCCCCT--------------------	1.10% (508 reads)
GGCCCTTCTCTCTGCCCCCTG--ACCTGAACCGTATATCC	1.05% (485 reads)
GGCCCTTCTCTCTGCCCCCTGG------AACCGTATATCC	0.85% (395 reads)
GGCCCTTCTCTCTGCCCCCTGGGGTACCTGAACCGTATAT	0.75% (347 reads)
GGCCCTTCTCTCTGCCCCCT---ACCTGAACCGTATATCC	0.71% (329 reads)
GGCCCTTCTCTCTGCCCC--------TGAACCGTATATCC	0.57% (266 reads)
GGCCCTTCTCTCTGCCCCCTG-----TGAACCGTATATCC	0.53% (244 reads)
GGCCCTTCTCTCTGCC----------TGAACCGTATATCC	0.45% (207 reads)
GGCCCTTCTCTCTG--------------AACCGTATATCC	0.41% (190 reads)
GGCCCTTCTCTCTGCCCCCTG----CTGAACCGTATATCC	0.34% (156 reads)
GGCCCTTCTCTCTG--------TACCTGAACCGTATATCC	0.34% (155 reads)
GGCCCTTCTCTCTGCCCC--------------GTATATCC	0.25% (116 reads)
GGCCCTTCTCTCTGCCCCCT-----CTGAACCGTATATCC	0.22% (104 reads)
--------------------------TGAACCGTATATCC	0.22% (104 reads)
GGCCCTTCTCTCTGCCCCC-------------GTATATCC	0.21% (99 reads)
---------------------------------TATATCC	0.20% (93 reads)

bold Substitutions
□ Insertions
\- Deletions
------ Predicted cleavage position

HEXA4

InDelphi

Summary of predictions at target site with gRNA: TCCTTCCAGTCAGGGCCATA

<table>
<tr><th>Alignment</th><th>Category</th><th>%</th></tr>
<tr><td>ATGTAGAAATCCTTCCAGTCAGGGCC|ATAGGATATACGGTTCAGGTACCAGG</td><td>Reference</td><td>-</td></tr>
<tr><td>ATGTAGAAATCCTTCCAGTCAGG---|-----ATATACGGTTCAGGTACCAGG</td><td>8-bp deletion</td><td>22.2</td></tr>
<tr><td>ATGTAGAAATCCTTCCAGTCAGGGCCCATAGGATATACGGTTCAGGTACCAGG</td><td>1-bp insertion</td><td>13.2</td></tr>
<tr><td>ATGTAGAAATCCTTCCAGTCAGGG--|-----ATATACGGTTCAGGTACCAGG</td><td>7-bp deletion</td><td>10.9</td></tr>
<tr><td>ATGTAGAAATCCTTCCAGTCAGGGCCTATAGGATATACGGTTCAGGTACCAGG</td><td>1-bp insertion</td><td>3.8</td></tr>
<tr><td>ATGTAGAAATCCTTCCAGTCAGGGCC|-TAGGATATACGGTTCAGGTACCAGG</td><td>1-bp deletion</td><td>3.2</td></tr>
<tr><td>ATGTAGAAATCCTTCCAGTCAGGGC-|ATAGGATATACGGTTCAGGTACCAGG</td><td>1-bp deletion</td><td>3.2</td></tr>
<tr><td>ATGTAGAAATCCTTCCAGTCAGG---|-------------------TACCAGG</td><td>22-bp deletion</td><td>3.2</td></tr>
<tr><td>ATGTAGAAATCCTTCCAGTCAGGGCCAATAGGATATACGGTTCAGGTACCAGG</td><td>1-bp insertion</td><td>3.1</td></tr>
<tr><td>ATGTAGAAATCCTTCCAG--------|----GATATACGGTTCAGGTACCAGG</td><td>12-bp deletion</td><td>2.3</td></tr>
<tr><td>ATGTAGAAATCCTTCCAGTCAGGG--|----GATATACGGTTCAGGTACCAGG</td><td>6-bp deletion</td><td>1.9</td></tr>
</table>

CRISPRESSO

Sequence	
ACCTGAACCGTATATCCTATGGCCCTGACTGGAAGGATTT-	Reference
sgRNA	
ACCTGAACCGTATATCCTATGGCCCTGACTGGAAGGATTT-	66.12% (32176 reads)
ACCTGAACCGTATATCCTAT[G]GGCCCTGACTGGAAGGATT-	12.19% (5932 reads)
ACCTGAACCGTATATCCT--------GACTGGAAGGATTT-	2.71% (1319 reads)
ACCTGAACCGTATATCCTAT-GCCCTGACTGGAAGGATTT-	2.33% (1135 reads)
ACCTGAACCGTATATCC--------CTGACTGGAAGGATTT-	1.73% (844 reads)
ACCTGAACCGTATATCCTATGG---------GAAGGATTT-	0.38% (185 reads)
ACCTGAACCGTATATCCTAT------GACTGGAAGGATTT-	0.28% (136 reads)
ACCTGA----------------------CTGGAAGGATTT-	0.26% (127 reads)
ACCTGAACCGTATATCCTAT----------GGAAGGATTT-	0.23% (114 reads)
ACCTGAACCGTATATCCT--GGCCCTGACTGGAAGGATTT-	0.22% (108 reads)
ACCTGAACCGTATA--------CCCTGACTGGAAGGATTT-	0.22% (105 reads)

bold Substitutions
□ Insertions
\- Deletions
------ Predicted cleavage position

HEXA5

InDelphi

Summary of predictions at target site with gRNA: AGTCAGGGCCATAGGATATA

<table>
<tr><th>Alignment</th><th>Category</th><th>%</th></tr>
<tr><td>AATCCTTCCAGTCAGGGCCATAGGAT|ATACGGTTCAGGTACCAGGGGGCAGA</td><td>Reference</td><td>-</td></tr>
<tr><td>AATCCTTCCAGTCAGGGCCATAGGATTATACGGTTCAGGTACCAGGGGGCAGA</td><td>1-bp insertion</td><td>38.1</td></tr>
<tr><td>AATCCTTCCAGTCAGGGCCATAGGAT|--ACGGTTCAGGTACCAGGGGGCAGA</td><td>2-bp deletion</td><td>15.3</td></tr>
<tr><td>AATCCTTCCAGTCAGGGCCATA----|---CGGTTCAGGTACCAGGGGGCAGA</td><td>7-bp deletion</td><td>9.6</td></tr>
<tr><td>AATCCTTCCAGTCAGGGCCATAGG--|------TTCAGGTACCAGGGGGCAGA</td><td>8-bp deletion</td><td>5.1</td></tr>
<tr><td>AATCCTTCCAGTCAGGGCCATAGG--|------------TACCAGGGGGCAGA</td><td>14-bp deletion</td><td>2.9</td></tr>
<tr><td>AATCCTTCCAGTCAGGGCCATAGGA-|ATACGGTTCAGGTACCAGGGGGCAGA</td><td>1-bp deletion</td><td>1.9</td></tr>
<tr><td>AATCCTTCCAGTCAGGGCCATAGGAT|-TACGGTTCAGGTACCAGGGGGCAGA</td><td>1-bp deletion</td><td>1.9</td></tr>
<tr><td>AATCCTTCCAGTCAGG----------|------------TACCAGGGGGCAGA</td><td>22-bp deletion</td><td>1.9</td></tr>
<tr><td>AATCCTTCCAGTCAGGGCCATAGGA-|---CGGTTCAGGTACCAGGGGGCAGA</td><td>4-bp deletion</td><td>1.7</td></tr>
<tr><td>AATCCTTCCAGTCAGGGCCATA----|-TACGGTTCAGGTACCAGGGGGCAGA</td><td>5-bp deletion</td><td>1.3</td></tr>
</table>

CRISPRESSO

Sequence	
CCCTGGTACCTGAACCGTATATCCTATGGCCCTGACTGGA-	Reference
sgRNA	
CCCTGGTACCTGAACCGTATATCCTATGGCCCTGACTGGA-	55.92% (27123 reads)
CCCTGGTACCTGAACCGTAT[A]ATCCTATGGCCCTGACTGG-	20.54% (9961 reads)
CCCTGGTACCTGAACCGTAT-------GGCCCTGACTGGA-	4.08% (1981 reads)
CCCTGGTACCTGAACC--------TATGGCCCTGACTGGA-	3.25% (1576 reads)
CCCTGGTACCTGAACCGTAT--CCTATGGCCCTGACTGGA-	1.12% (543 reads)
CCCTGGTACCTGAACCGTATAT---ATGGCCCTGACTGGA-	0.57% (275 reads)
CCCTGGTACCTGAACCGTATA-CCTATGGCCCTGACTGGA-	0.52% (251 reads)
CCCTGGTACCTGAACCGTATA--CTATGGCCCTGACTGGA-	0.48% (231 reads)
CCCTGGTACCTGAACCGTAT-TCCTATGGCCCTGACTGGA-	0.42% (204 reads)
CCCTGGTACCTGA----------------------CTGGA-	0.36% (177 reads)
CCCTGGTACCTGAACCGTAT[AT]ATCCTATGGCCCTGACTG-	0.35% (168 reads)
CCCTGGTACCTGAACCGTAT-----ATGGCCCTGACTGGA-	0.33% (160 reads)
CCCTGGTACCT--------------ATGGCCCTGACTGGA-	0.33% (159 reads)
CCCTGGTACCTGAAC---------------------TGGA-	0.26% (128 reads)
CCCTGGTACCTGAA-------TCCTATGGCCCTGACTGGA-	0.26% (126 reads)
CCCTGGTACCTGAACCGTAT[AA]ATCCTATGGCCCTGACTG-	0.26% (124 reads)
CCCTGGTACCTGA--------TCCTATGGCCCTGACTGGA-	0.24% (117 reads)
CCCTGGTACCTGAACCGTAT---CTATGGCCCTGACTGGA-	0.23% (110 reads)

bold Substitutions
□ Insertions
\- Deletions
------ Predicted cleavage position

HIRA

InDelphi

Summary of predictions at target site with gRNA: GAAGACCAAGGATAGACTGC

Alignment	Category	%
GGGCAAAGTGAAGACCAAGGATAGAC|TGCTGGGCTTGACAGCATGGAGGGTG	Reference	-
GGGCAAAGTGAAGACCAAGGATAGAC|-------------AGCATGGAGGGTG	13-bp deletion	9.4
GGGCAAAGTGAAGACCAAGGATAGACCTGCTGGGCTTGACAGCATGGAGGGTG	1-bp insertion	7.1
GGGCAAAGTGAAGACCAAGGATAGAC|---TGGGCTTGACAGCATGGAGGGTG	3-bp deletion	5.9
GGGCAAAGTGAAGACCAAGGATAGAC|-GCTGGGCTTGACAGCATGGAGGGTG	1-bp deletion	4.8
GGGCAAAGTGAAGACCAAGGATAGA-|TGCTGGGCTTGACAGCATGGAGGGTG	1-bp deletion	4.8
GGGCAAAGTGAAGACCAAGG------|------GCTTGACAGCATGGAGGGTG	12-bp deletion	4.6
GGGCAAAGTGAAGACCAAGGATAG--|--CTGGGCTTGACAGCATGGAGGGTG	4-bp deletion	4.6
GGGCAAAGTGAAGACCAAGG------|-------CTTGACAGCATGGAGGGTG	13-bp deletion	3.6
GGGCAAAGTGAAGACCAAGGAT----|-GCTGGGCTTGACAGCATGGAGGGTG	5-bp deletion	3.3
GGGCAAAGTGAAGACCAAGGATAG--|-GCTGGGCTTGACAGCATGGAGGGTG	3-bp deletion	2.8

CRISPRESSO

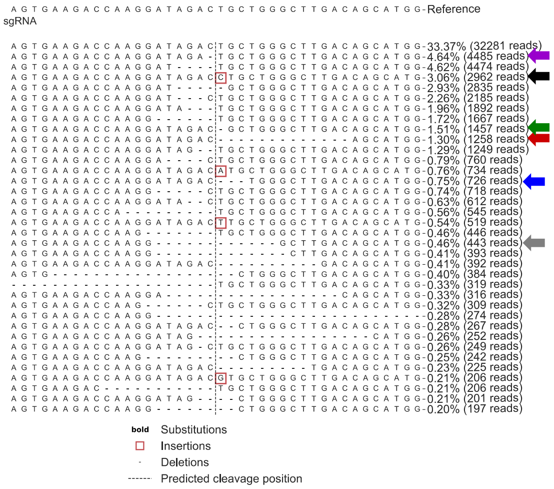

LMNA

InDelphi

Summary of predictions at target site with gRNA: GGAGCTCAATGATCGCTTGG

Alignment	Category	%
GGACCTGCAGGAGCTCAATGATCGCT|TGGCGGTCTACATCGACCGTGTGCGC	Reference	-
GGACCTGCAGGAGCTCAATGATCGCTTTGGCGGTCTACATCGACCGTGTGCGC	1-bp insertion	23.0
GGACCTGCAGGAGCTCAATGATCGC-|----GGTCTACATCGACCGTGTGCGC	5-bp deletion	14.3
GGACCTGCAGGAGCTCAATGATCGCT|-GGCGGTCTACATCGACCGTGTGCGC	1-bp deletion	9.9
GGACCTGCAGGAGCTCAATGATCG--|-----GTCTACATCGACCGTGTGCGC	7-bp deletion	8.7
GGACCTGCAGGAGCTCAATG------|--GCGGTCTACATCGACCGTGTGCGC	8-bp deletion	4.9
GGACCTGCAGGAGCTCAATGATCGCT|---------ACATCGACCGTGTGCGC	9-bp deletion	3.8
GGACCTGCAGGAGCTCAATGATCG--|---------------ACCGTGTGCGC	17-bp deletion	3.6
GGACCTGCAGGAGCTCAATGATCG--|--GCGGTCTACATCGACCGTGTGCGC	4-bp deletion	2.4
GGACCTGCAGGAGCTCAATGATC---|--------TACATCGACCGTGTGCGC	11-bp deletion	2.3
GGACCTGCAGGAGCTCAATGAT----|-GGCGGTCTACATCGACCGTGTGCGC	5-bp deletion	1.8

CRISPRESSO

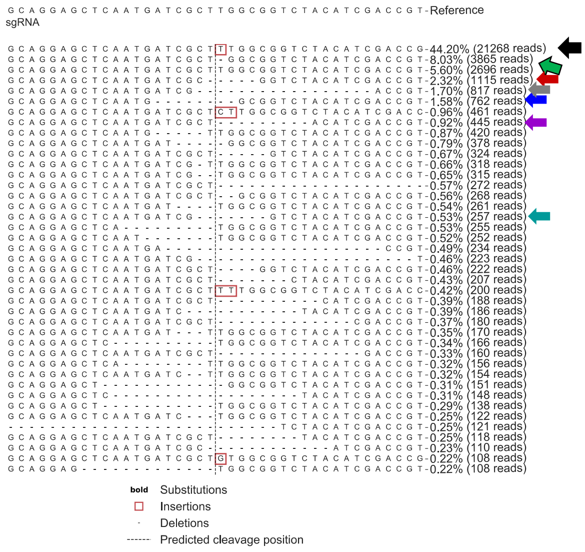

MMACHC

InDelphi

HEK293T

Summary of predictions at target site with gRNA: TTTCTATCTGCAGCTCTGGG

<table>
<tr><th>Alignment</th><th>Category</th><th>%</th></tr>
<tr><td>CAGCAATGATTTCTATCTGCAGCTCT|GGGAGGCTCTGGAACAGTGGACAAAA</td><td>Reference</td><td>-</td></tr>
<tr><td>CAGCAATGATTTCTATCTGCAGCTCTTGGGAGGCTCTGGAACAGTGGACAAAA</td><td>1-bp insertion</td><td>39.2</td></tr>
<tr><td>CAGCAATGATTTCTATCTGCAGCTCT|----------GGAACAGTGGACAAAA</td><td>10-bp deletion</td><td>31.4</td></tr>
<tr><td>CAGCAATGATTTCTATCTGCAGCTCT|--------CTGGAACAGTGGACAAAA</td><td>8-bp deletion</td><td>3.0</td></tr>
<tr><td>CAGCAATGATTTCTATCTGCAG----|-----GCTCTGGAACAGTGGACAAAA</td><td>9-bp deletion</td><td>2.4</td></tr>
<tr><td>CAGCAATGATTTCTATCTG-------|-----------GAACAGTGGACAAAA</td><td>18-bp deletion</td><td>1.7</td></tr>
<tr><td>CAGCAATGATTTCTATCTGCAGCTC-|GGGAGGCTCTGGAACAGTGGACAAAA</td><td>1-bp deletion</td><td>1.6</td></tr>
<tr><td>CAGCAATGATTTCTATCTGCAGCTCT|-GGAGGCTCTGGAACAGTGGACAAAA</td><td>1-bp deletion</td><td>1.6</td></tr>
<tr><td>CAGCAATGATTTCTATCTGCAGCTCTAGGGAGGCTCTGGAACAGTGGACAAAA</td><td>1-bp insertion</td><td>1.3</td></tr>
<tr><td>CAGCAATGATTTCTATCTGC------|-------TCTGGAACAGTGGACAAAA</td><td>13-bp deletion</td><td>1.2</td></tr>
<tr><td>CAGCAATGATTTCTATCTGCAG----|-GGAGGCTCTGGAACAGTGGACAAAA</td><td>5-bp deletion</td><td>1.2</td></tr>
</table>

K562

Summary of predictions at target site with gRNA: TTTCTATCTGCAGCTCTGGG

<table>
<tr><th>Alignment</th><th>Category</th><th>%</th></tr>
<tr><td>CAGCAATGATTTCTATCTGCAGCTCT|GGGAGGCTCTGGAACAGTGGACAAAA</td><td>Reference</td><td>-</td></tr>
<tr><td>CAGCAATGATTTCTATCTGCAGCTCT|----------GGAACAGTGGACAAAA</td><td>10-bp deletion</td><td>35.5</td></tr>
<tr><td>CAGCAATGATTTCTATCTGCAGCTCTTGGGAGGCTCTGGAACAGTGGACAAAA</td><td>1-bp insertion</td><td>32.8</td></tr>
<tr><td>CAGCAATGATTTCTATCTGCAGCTCT|--------CTGGAACAGTGGACAAAA</td><td>8-bp deletion</td><td>3.4</td></tr>
<tr><td>CAGCAATGATTTCTATCTGCAG----|-----GCTCTGGAACAGTGGACAAAA</td><td>9-bp deletion</td><td>2.7</td></tr>
<tr><td>CAGCAATGATTTCTATCTG-------|-----------GAACAGTGGACAAAA</td><td>18-bp deletion</td><td>1.9</td></tr>
<tr><td>CAGCAATGATTTCTATCTGCAGCTC-|GGGAGGCTCTGGAACAGTGGACAAAA</td><td>1-bp deletion</td><td>1.8</td></tr>
<tr><td>CAGCAATGATTTCTATCTGCAGCTCT|-GGAGGCTCTGGAACAGTGGACAAAA</td><td>1-bp deletion</td><td>1.8</td></tr>
<tr><td>CAGCAATGATTTCTATCTGC------|-------TCTGGAACAGTGGACAAAA</td><td>13-bp deletion</td><td>1.3</td></tr>
<tr><td>CAGCAATGATTTCTATCTGCAG----|-GGAGGCTCTGGAACAGTGGACAAAA</td><td>5-bp deletion</td><td>1.3</td></tr>
<tr><td>CAGCAATGATTTCTATCTGCAGCT--|----------GGAACAGTGGACAAAA</td><td>12-bp deletion</td><td>1.3</td></tr>
</table>

CRISPRESSO

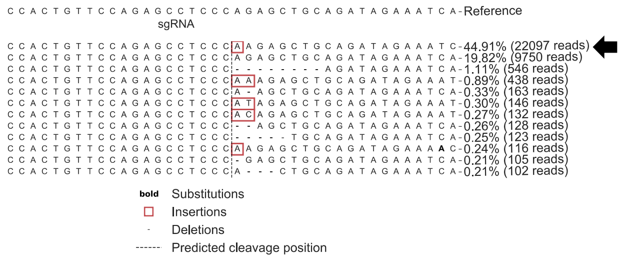

PCSK

InDelphi

Summary of predictions at target site with gRNA: AGGCTGCAGCTCCCACTGGG

<table>
<tr><th>Alignment</th><th>Category</th><th>%</th></tr>
<tr><td>CTCTGCCCCAGGCTGCAGCTCCCACT|GGGAGGTGGAGGACCTTGGCACCCAC</td><td>Reference</td><td>-</td></tr>
<tr><td>CTCTGCCCCAGGCTGCAGCTCCCACTTGGGAGGTGGAGGACCTTGGCACCCAC</td><td>1-bp insertion</td><td>37.8</td></tr>
<tr><td>CTCTGCCCCAGGCTGCAGCTCCCAC-|GGGAGGTGGAGGACCTTGGCACCCAC</td><td>1-bp deletion</td><td>4.5</td></tr>
<tr><td>CTCTGCCCCAGGCTGCAGCTCCCACT|-GGAGGTGGAGGACCTTGGCACCCAC</td><td>1-bp deletion</td><td>4.5</td></tr>
<tr><td>CTCTGCCCCAGGCTGCAG--------|-----GTGGAGGACCTTGGCACCCAC</td><td>13-bp deletion</td><td>2.5</td></tr>
<tr><td>CTCTGCCCCAGGCTGCAGCTCCCA--|----GGTGGAGGACCTTGGCACCCAC</td><td>6-bp deletion</td><td>2.4</td></tr>
<tr><td>CTCTGCCCCAGGCTGCAGCTCCCA--|GGGAGGTGGAGGACCTTGGCACCCAC</td><td>2-bp deletion</td><td>2.3</td></tr>
<tr><td>CTCTGCCCCAGGCTGCAGCTCCCACT|--GAGGTGGAGGACCTTGGCACCCAC</td><td>2-bp deletion</td><td>2.3</td></tr>
<tr><td>CTCTGCCCCAGGCTGCAGCTCCCAC-|-GGAGGTGGAGGACCTTGGCACCCAC</td><td>2-bp deletion</td><td>2.3</td></tr>
<tr><td>CTCTGCCCCAGGCTGCAGCTCCCAC-|--------------------------</td><td>27-bp deletion</td><td>1.9</td></tr>
<tr><td>CTCTGCCCCAGGCTGCAGCTCCCACT|-------GGAGGACCTTGGCACCCAC</td><td>7-bp deletion</td><td>1.9</td></tr>
</table>

CRISPRESSO

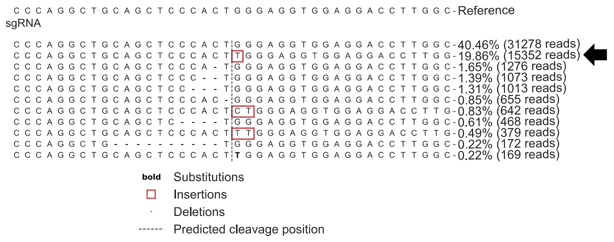

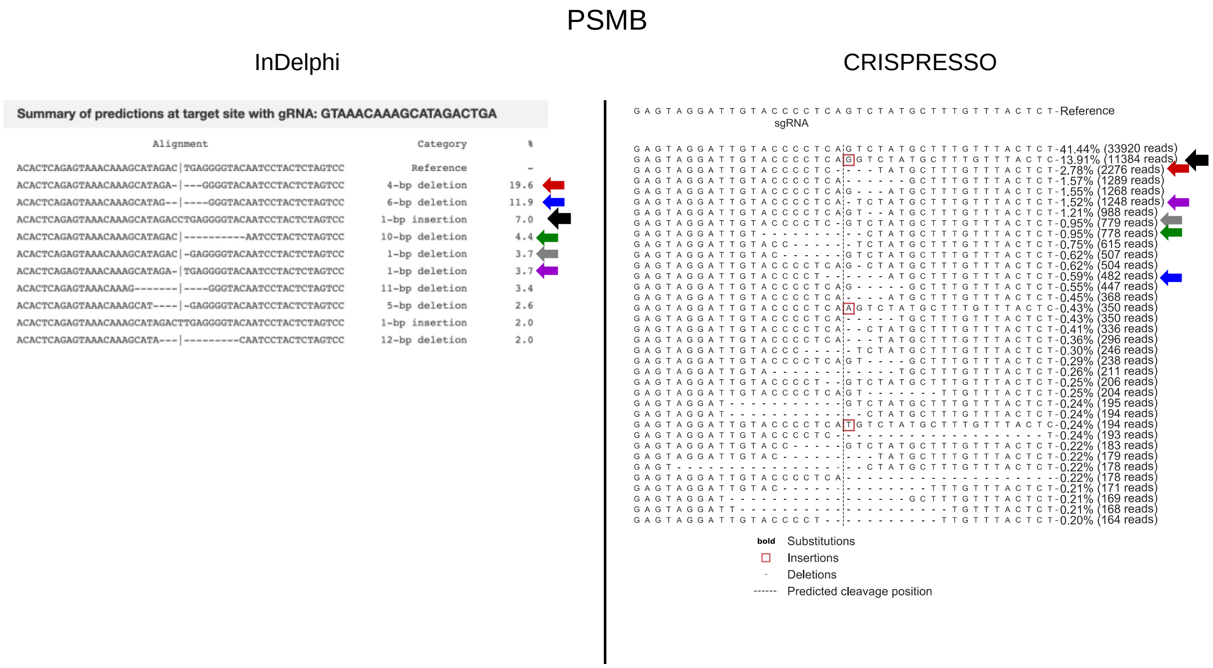

PSMB
InDelphi
CRISPRESSO
Summary of predictions at target site with gRNA: GTAAACAAAGCATAGACTGA
Alignment
Category
%
Reference
sgRNA
bold Substitutions
Insertions
Deletions
Predicted cleavage position

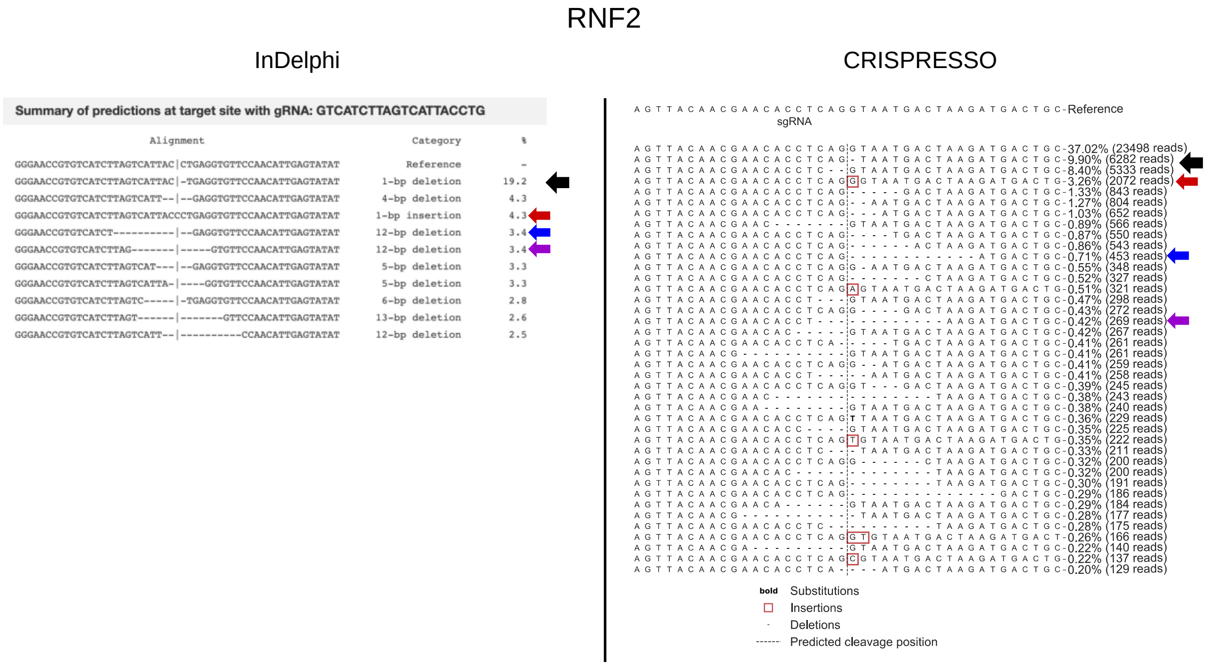

RNF2
InDelphi
CRISPRESSO
Summary of predictions at target site with gRNA: GTCATCTTAGTCATTACCTG
Alignment
Category
%
Reference
sgRNA
bold Substitutions
Insertions
Deletions
Predicted cleavage position

SEC61B

InDelphi

CRISPRESSO

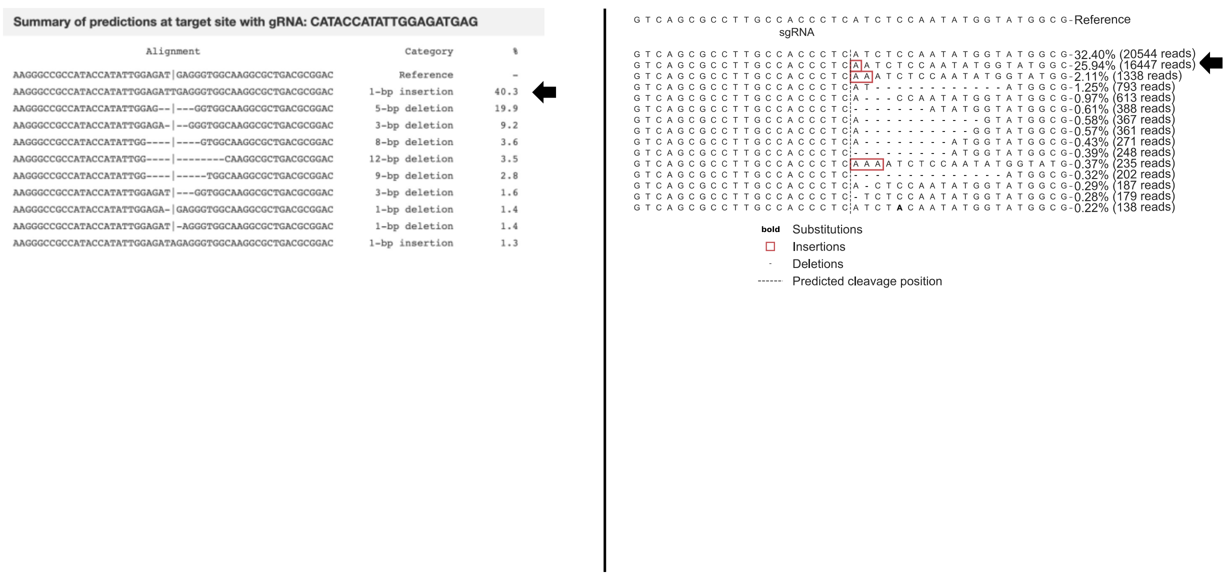

Supplementary Figure 1. InDelphi predictions (left) and experimentally determined indel sequences (right) for all genomic loci studied in this work. The InDelphi figures show predicted indel sequences in HEK293T cells, except for the *APOB1* and *MMACHC* sites, where predicted indels for both HEK293 and K562 cells are shown. The CRISPResso analysis of HTS data from treated HEK293T cells using the indel output is shown on the right. All sites studied in the paper are listed and labelled at the top. Indel sequences with rates above 5% for both the experimental samples and the InDelphi predictions are marked with arrows.

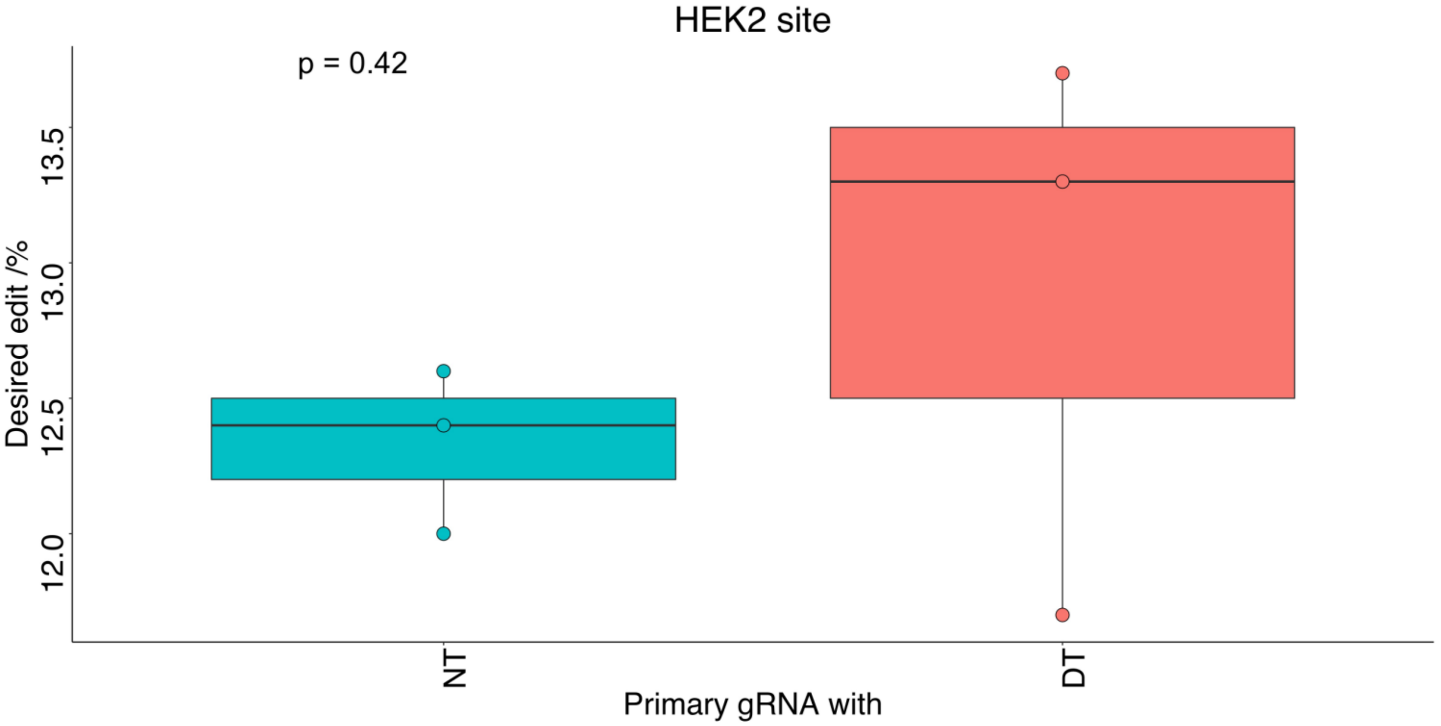

Supplementary Figure 2. Improvements in HDR-mediated genome editing with ssODNs using the double tap method at the *HEK2* site. HEK293T cells were transfected with ssODN, Cas9 plasmid, and gRNA plasmids. After 72 hours, cells were analyzed by NGS and HDR-mediated genome editing efficiencies were quantified. Shown are the percent of DNA sequencing reads with the desired modification introduced (perfect HDR products without indels) for cells treated with primary gRNA and a non-targeting gRNA (NT, left), or primary gRNA and secondary gRNA(s) (DT, right). Values on the whisker plots represent the lowest observation, lower quartile, median, upper quartile and the highest observation of three independent replicates. Each replicate is marked individually. Data were analyzed with univariate statistics (one-way ANOVA [one-sided]) and p-values are labelled on the graph. Data points are marked as circles when the ssODN encoded an extra blocking mutation, and as triangles when no additional mutation was installed.

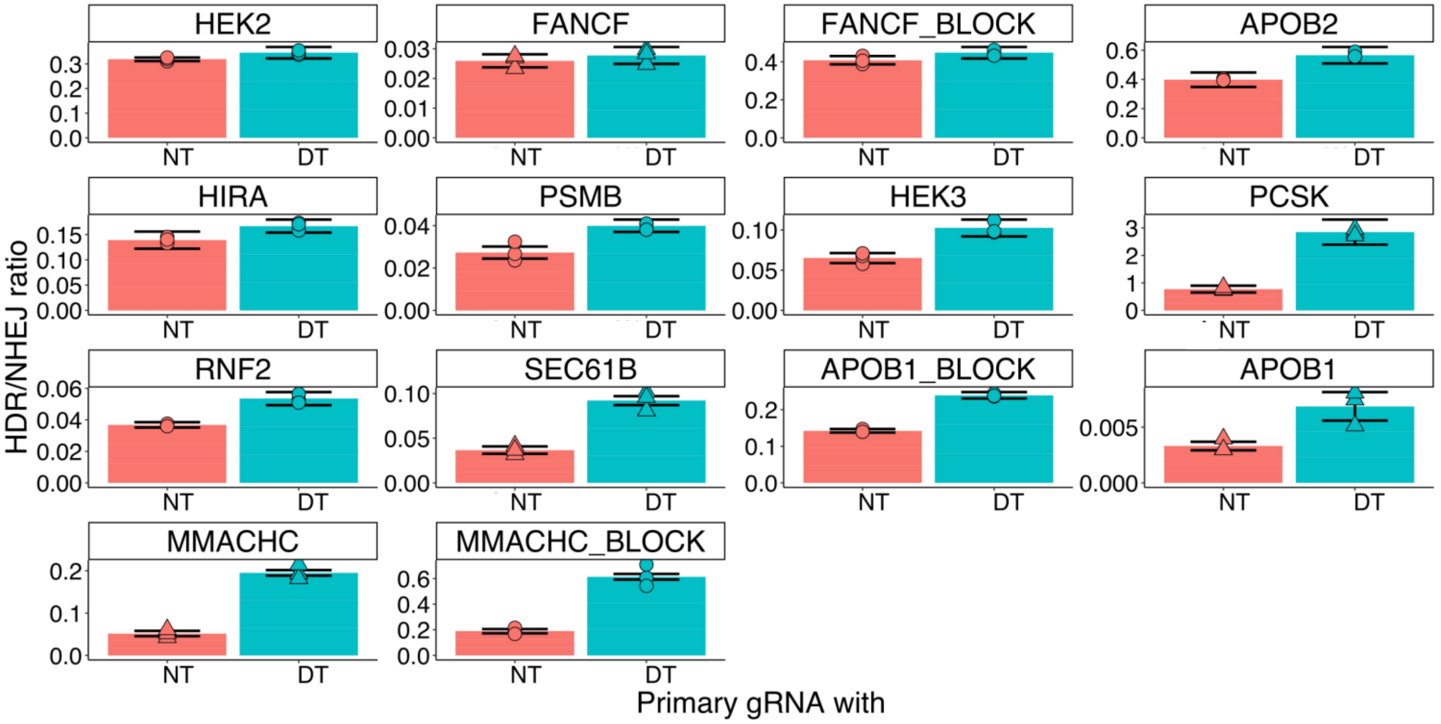

Supplementary Figure 3. Improvements in HDR to NHEJ ratios using the double tap method. HEK293T cells were transfected with ssODN, Cas9 plasmid, and gRNA plasmids. After 72 hours, cells were analyzed by NGS and HDR-mediated genome editing efficiencies and indel frequencies were quantified. Shown are the percent of DNA sequencing reads with the desired modification introduced (perfect HDR products without indels) divided by the percent of DNA sequencing reads with indels for cells treated with primary gRNA and a non-targeting gRNA (NT), or primary gRNA and secondary gRNA(s) (DT). Values and error bars represent the mean and propagation of uncertainty of the changes of the ratios of three independent replicates.

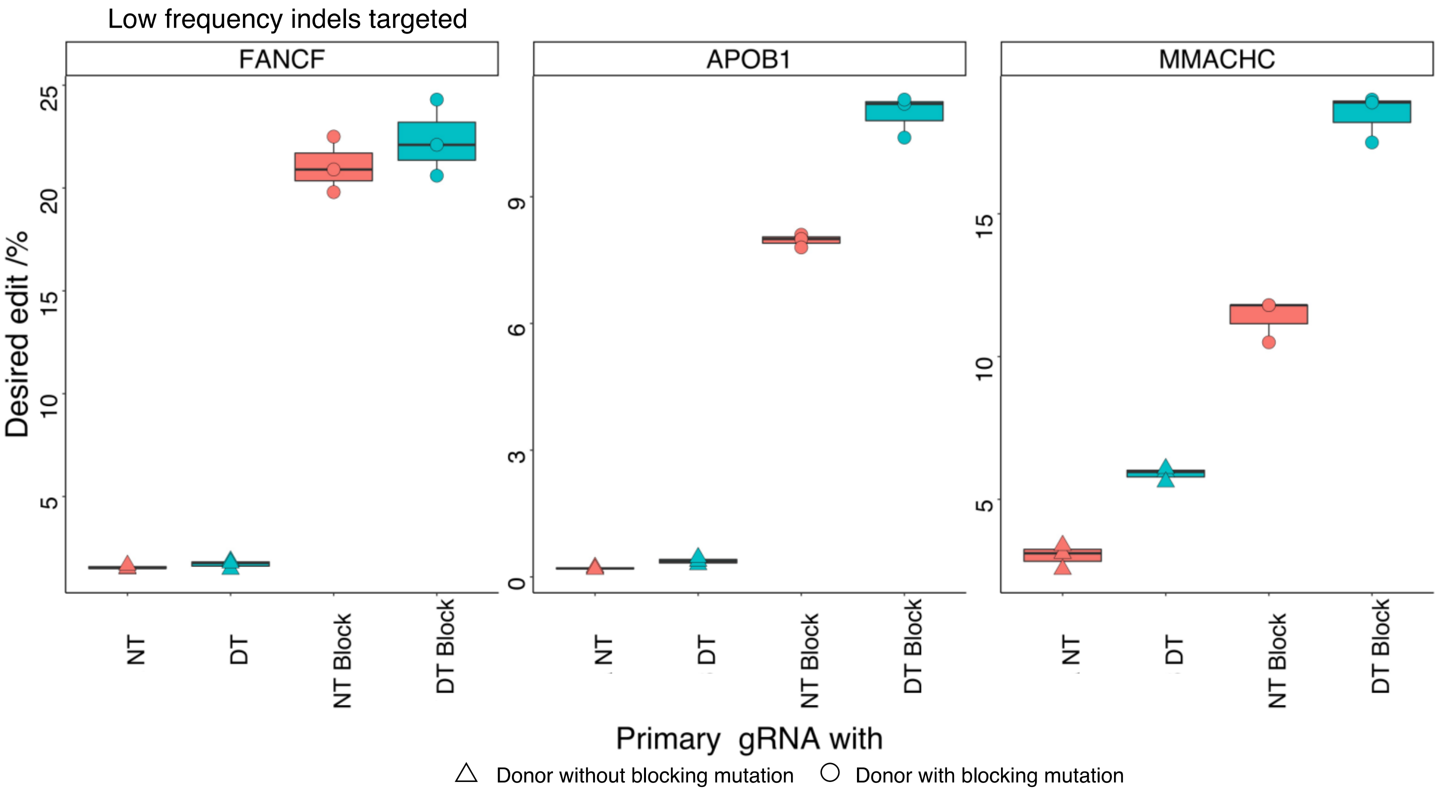

Supplementary Figure 4. Combined improvements in HDR-mediated genome editing using the double tap method and ssODN blocking mutations at the *FANCF* (in which a low-frequency indel was targeted), *APOB1* and *MMACHC* sites. HEK293T cells were transfected with ssODN, Cas9 plasmid, and gRNA plasmids. After 72 hours, cells were analyzed by NGS and HDR-mediated genome editing efficiencies were quantified. Shown are the percent of DNA sequencing reads with the desired modification introduced (perfect HDR products without indels) for cells treated with primary gRNA and a non-targeting gRNA (NT), or primary gRNA and secondary gRNA(s) (DT). Donor templates with blocking mutations (Block) and without were tested. Values on the whisker plots represent the lowest observation, lower quartile, median, upper quartile and the highest observation of three independent replicates. Each replicate is marked individually. Data points are marked as circles when the ssODN encoded an extra blocking mutation, and as triangles when no additional mutation was installed.

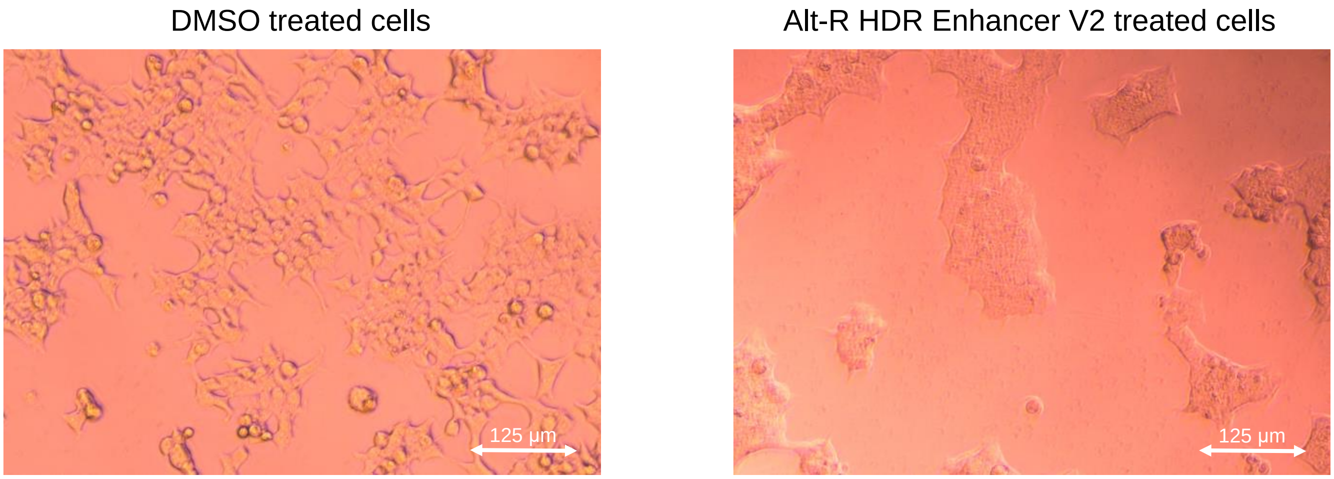

Supplementary Figure 5. Morphology changes of HEK293T cells after dimethyl sulfoxide (DMSO) and Alt-R ™ HDR Enhancer V2 treatment 24 hours after transfection. All the Alt-R ™ HDR Enhancer V2 treated samples displayed the morphological changes displayed above. Removal of the Alt-R ™ HDR Enhancer V2-containing media followed by replating of the cells resulted in a return to normal morphology after 24 hours. Shown are representative images from one replicate; the experiment was conducted three independent times.

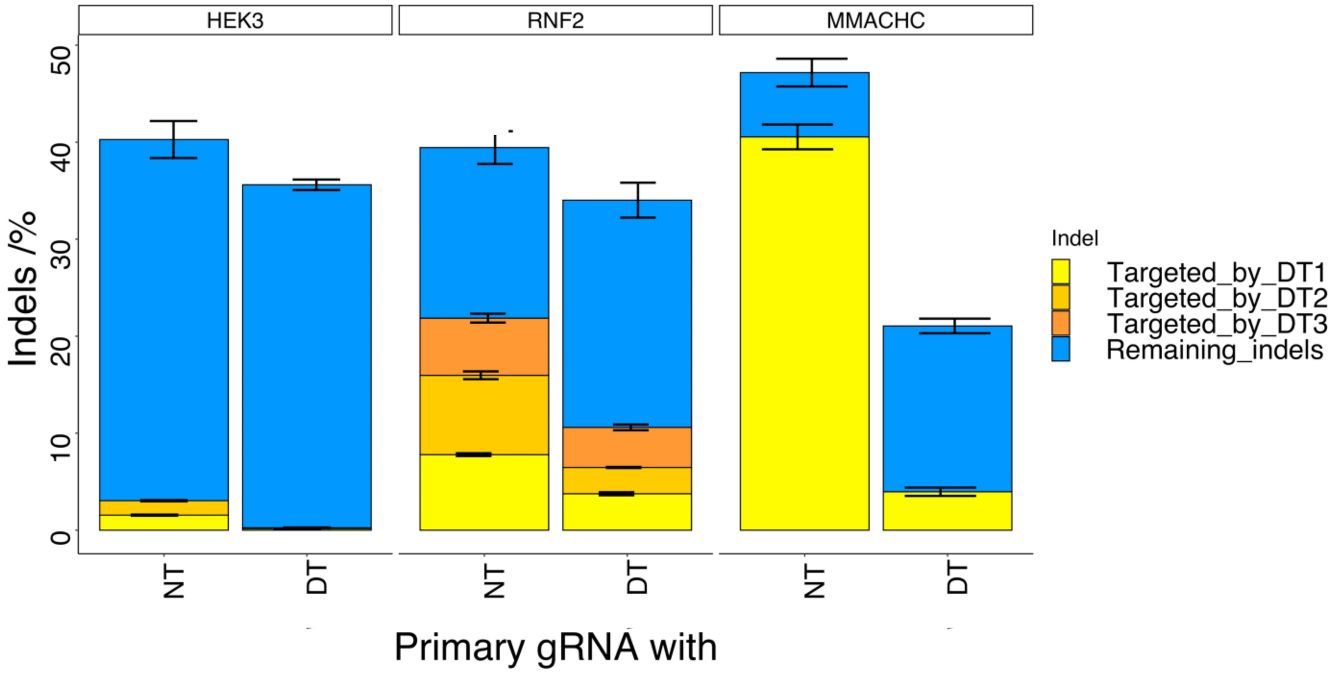

Supplementary Figure 6. Indel frequencies for Cas9 ribonucleoprotein (RNP)-treated cells. HEK293T cells were transfected with Cas9 with primary gRNA and a non-targeting gRNA (NT, left), or primary gRNA and secondary gRNA(s) (DT, right) and ssODNs. After 72 hours, cells were analyzed by NGS and indels were quantified with CRISPResso2. Blue bars show the absolute (or total) indel NGS read frequency and yellow-orange bars show the indel frequencies of potential target with double tap gRNAs. Values and error bars represent the mean of the number of sequencing reads with indel sequences divided by the total number of sequencing reads ± SD for n = 3 biological replicates.

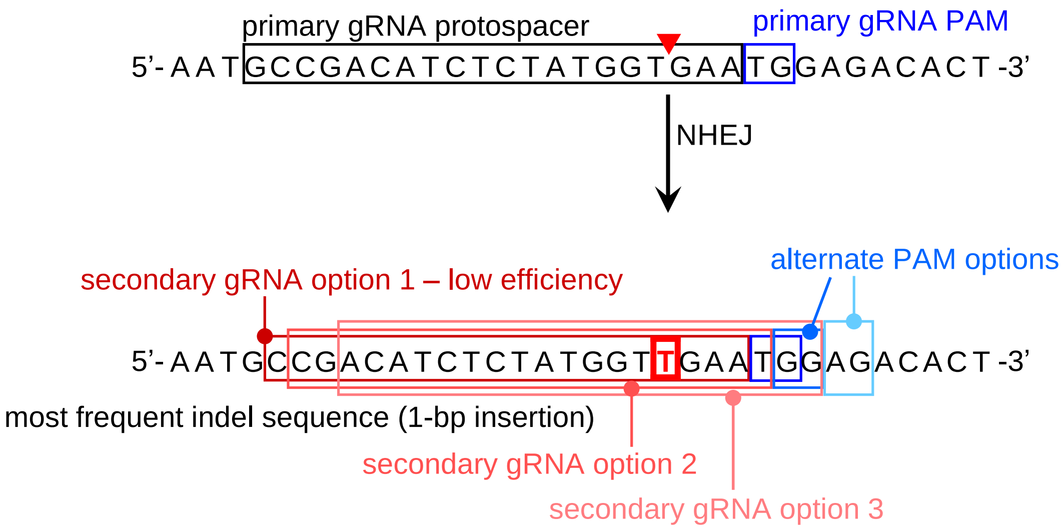

Supplementary Figure 7. Secondary and alternative secondary gRNAs for the *APOB1* site to target the most frequent indel (a 1-bp insertion product). Note for ease of design, we would use the Cas9-NG variant, which recognizes an NG PAM (relaxed from NGG).

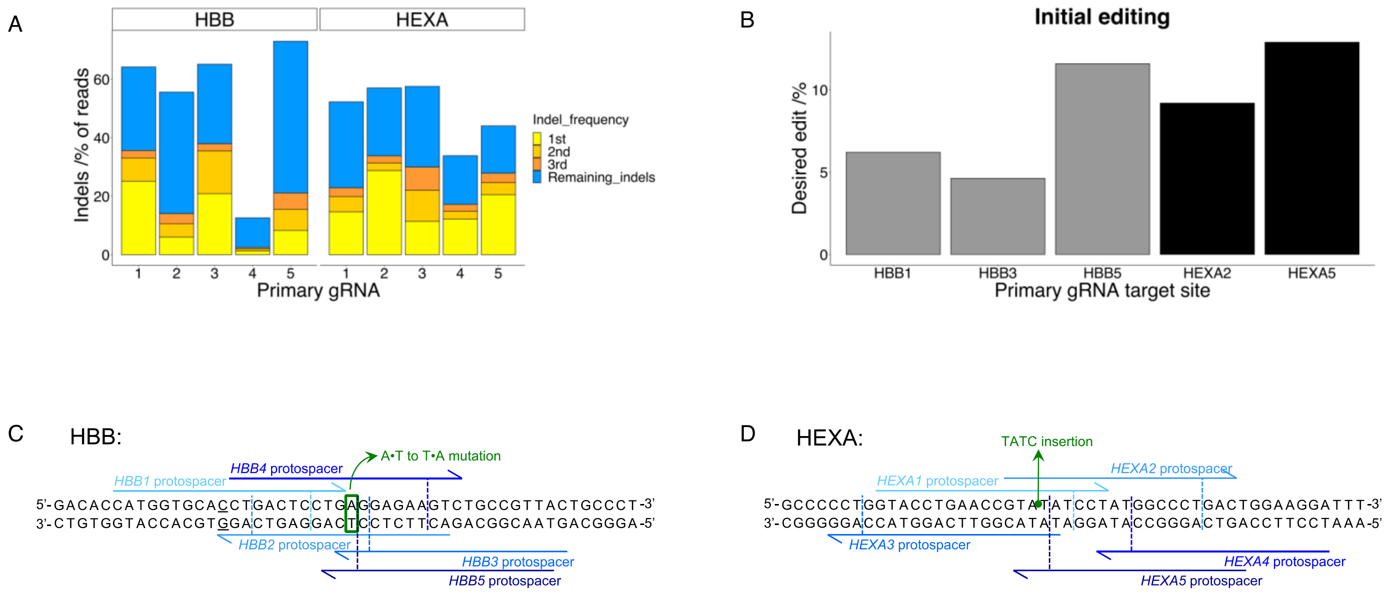

Supplementary Figure 8. Indel frequencies generated with candidate primary gRNAs at the *HBB* and *HEXA* loci. (A) HEK293T cells were transfected with Cas9 and gRNA plasmids. After 72 hours, cells were analyzed by NGS and HDR-mediated genome editing efficiencies were quantified. Shown are total indel rates of all samples, with the top three frequency indels shown in yellow, orange, and red. Blue represents the remaining indels. (B) HEK293T cells were transfected with ssODN and plasmids encoding Cas9 and candidate primary gRNAs selected from (A). After 72 hours, cells were analyzed by NGS and HDR-mediated genome editing efficiencies were quantified. Shown are the percent of DNA sequencing reads with the desired modification introduced (perfect HDR products without indels). (C and D) Genomic DNA sequences of the *HBB* (C) and *HEXA* (D) loci, with the modification of interest indicated in green, and the candidate primary gRNA protospacers indicated in blue, with their respective cut sites indicated with dotted lines. Results show a single experiment. Selected candidate primary RNAs were further tested to confirm high frequency of selected indel products. The underlined base pair indicates a SNV in the HEK293T cell line.

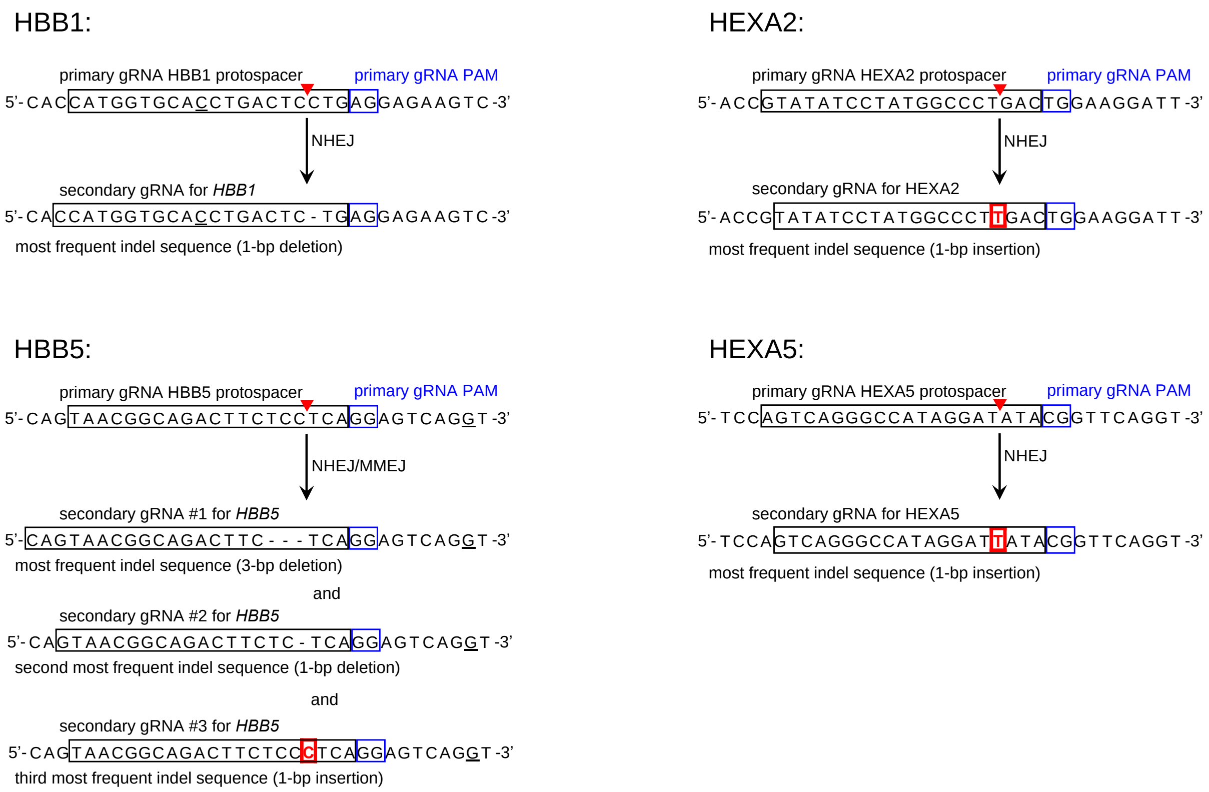

Supplementary Figure 9. Secondary gRNA designs for the *HBB1*, *HBB5*, *HEXA2* and *HEXA5* primary gRNAs. The underlined base pair indicates a SNV in the HEK293T cell line.

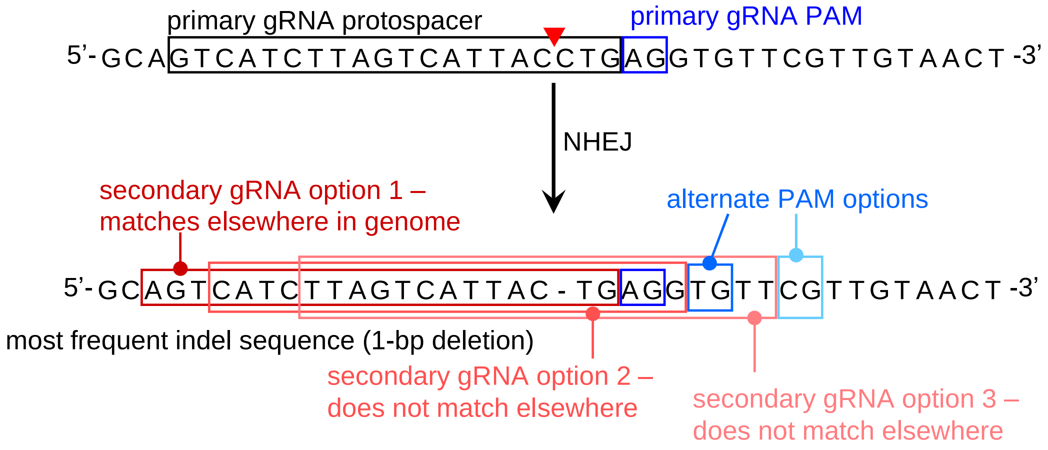

Supplementary Figure 10. Alternative secondary gRNA designs at the *RNF2* site to avoid unwanted off-target editing.

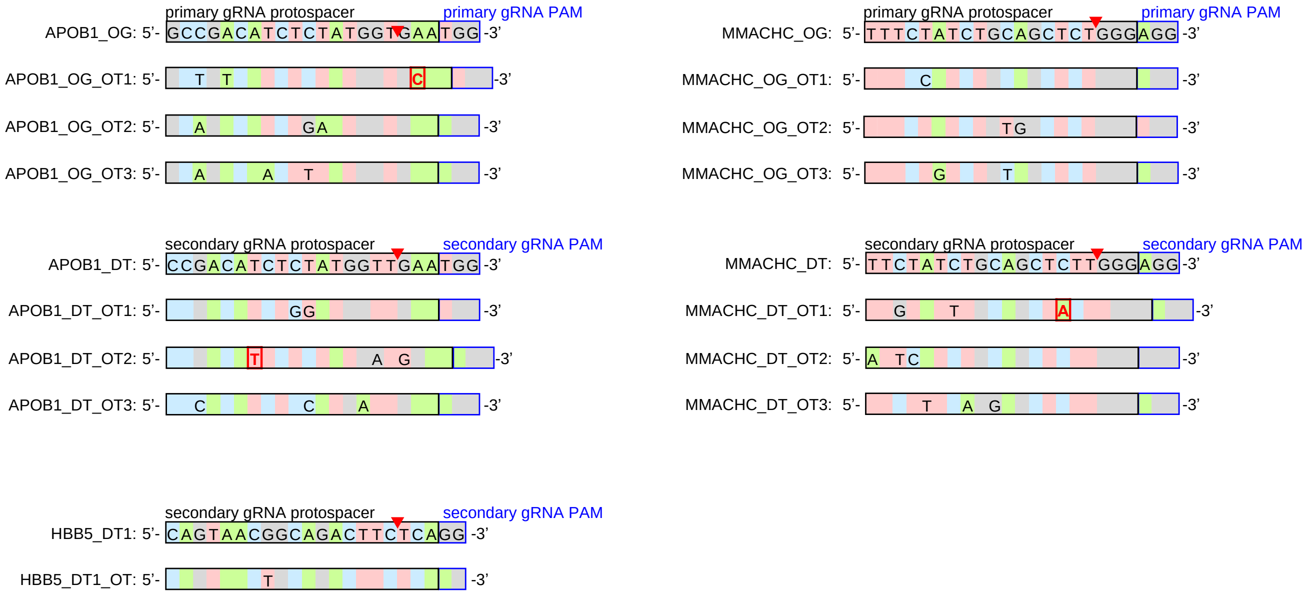

Supplementary Figure 11. Sequences of primary and secondary protospacers and PAMs, and their respective off-target sites that we evaluated for the *APOB1*, *MMACHC* and *HBB5* sites. Bases that are explicitly written in the off-target sites represent a mismatch, and bases in bolded red indicated bulges.

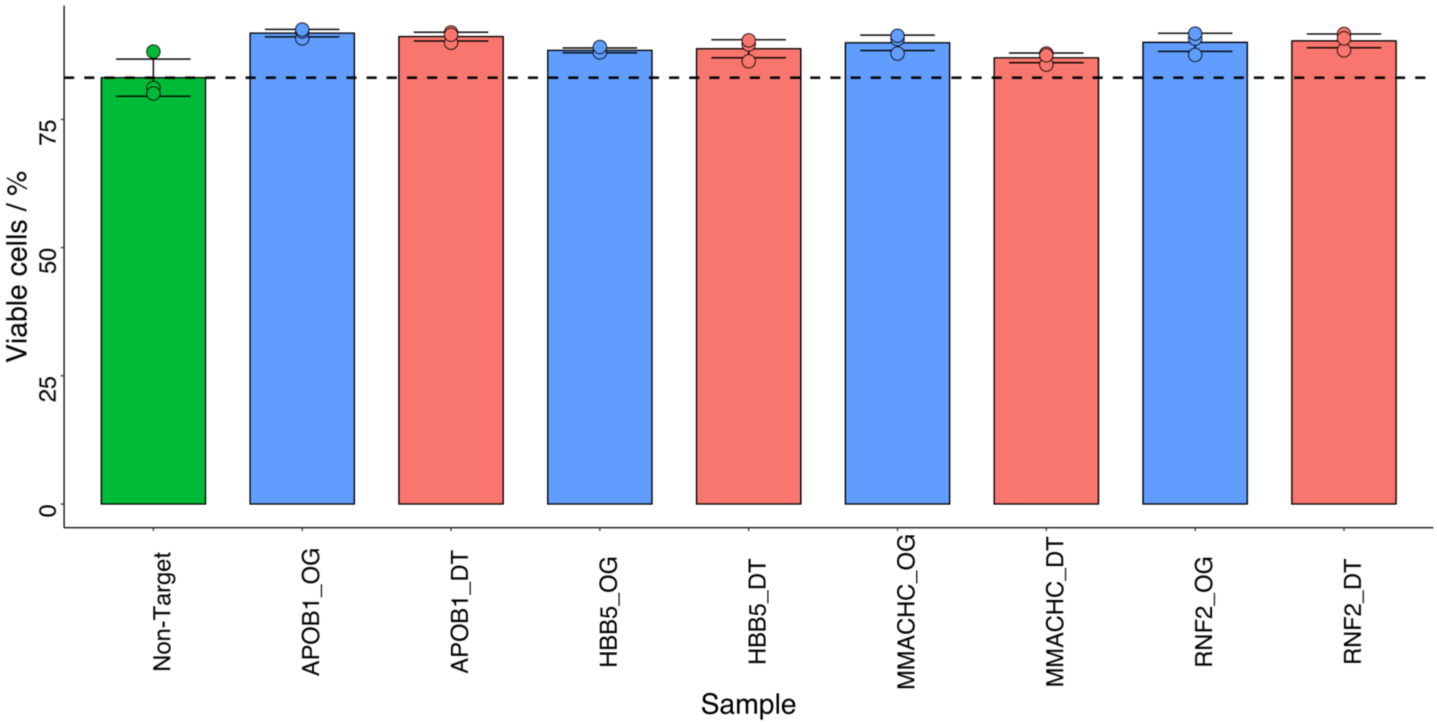

Supplementary Figure 12. Assessment of the effect of secondary gRNAs on cell viability. HEK293T cells were transfected with plasmids encoding Cas9-P2A-GFP, primary gRNA, and either non-targeting gRNA or secondary gRNA(s). As a control, HEK293T were transfected with plasmids encoding Cas9-P2A-GFP and non-targeting gRNA only. After 72 hours cells were stained with propidium iodide to quantify cell viability FACS. The percentage of all cells (both transfected and non-transfected) that were viable are plotted with respect to the primary gRNA used (*RNF2*, *HBB5*, *APOB1* and *MMACHC*). Samples with primary and non-targeting gRNAs are shown in blue, while those with primary and secondary gRNAs are in pink. The sample with non-targeting gRNA only is in green. OG stands for primary gRNA and DT stands for secondary gRNA. Values and error bars represent the mean and standard deviation of viable cells within the transfected population for n = 3 biological replicates.

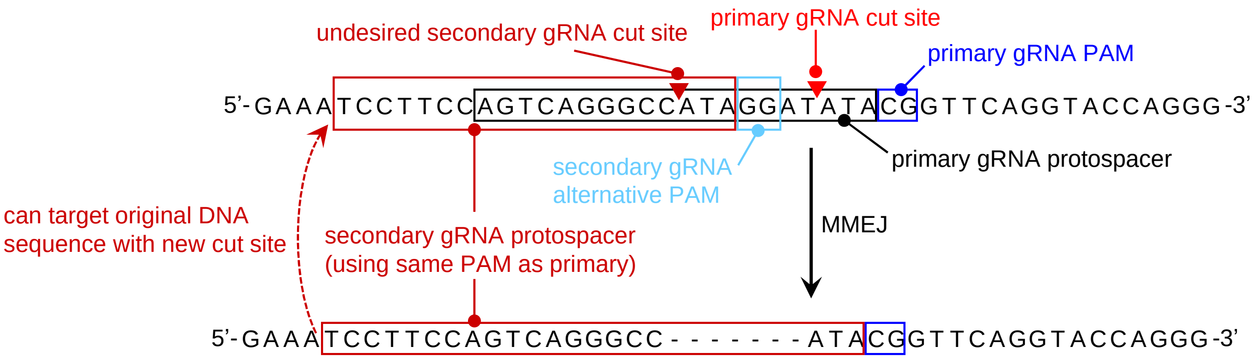

Supplementary Figure 13. The design of secondary gRNAs when indels with small deletions (likely facilitated by MMEJ) are targeted can result in a secondary gRNA that targets the original DNA sequence, but with an undesired alternate cut site. One such example (the *HEXA5* primary gRNA produces a 7-bp deletion indel with a high frequency) is explicitly shown. If a secondary gRNA is designed for the indel shown using the same PAM as the primary gRNA, it can target the original DNA sequence using a different PAM. To avoid this, an alternative PAM can be used.

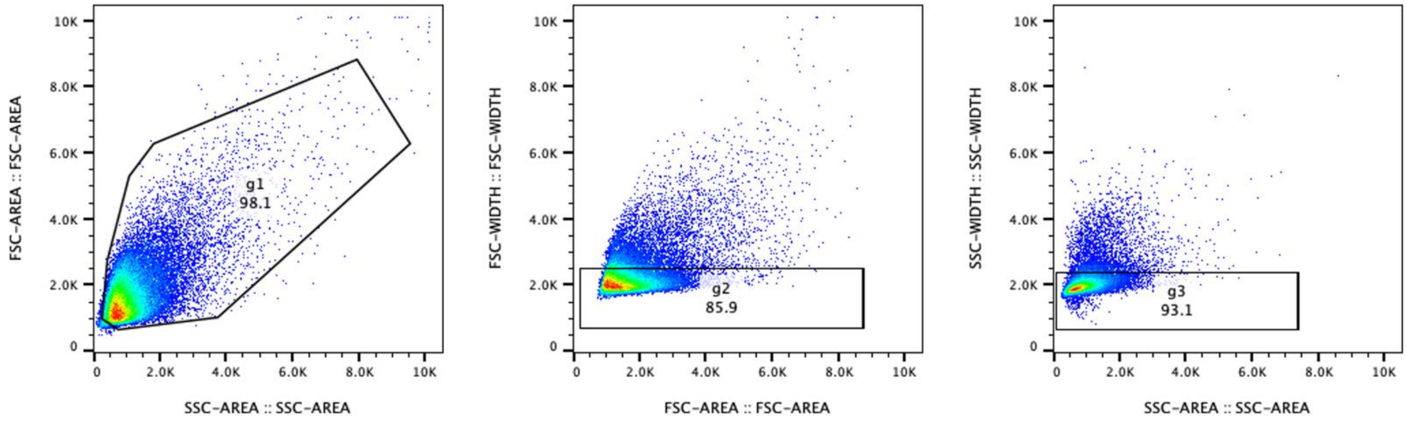

Supplementary Figure 14. Example of flow cytometry and FACS gating. Doublets were gated out using forward and side scattering width against area, and GFP gates were set using untransfected cells.
