# Supplementary material for: Targeting double-strand break indel byproducts with secondary guide RNAs improves Cas9 HDR-mediated genome editing efficiencies
Source: Nat Commun. 2022 May 9;13:2351. doi: 10.1038/s41467-022-29989-9 (PMC9085776; doi:10.1038/s41467-022-29989-9)
Supplement: Supplementary file 2 — Reporting Summary [file 41467_2022_29989_MOESM2_ESM.pdf]

nature portfolio

Corresponding author(s): Komor, Alexis C.

Last updated by author(s): Feb 22, 2022

Reporting Summary

Nature Portfolio wishes to improve the reproducibility of the work that we publish. This form provides structure for consistency and transparency in reporting. For further information on Nature Portfolio policies, see our Editorial Policies and the Editorial Policy Checklist.

Statistics

For all statistical analyses, confirm that the following items are present in the figure legend, table legend, main text, or Methods section.

n/a	Confirmed	
☐	☒	The exact sample size (*n*) for each experimental group/condition, given as a discrete number and unit of measurement
☐	☒	A statement on whether measurements were taken from distinct samples or whether the same sample was measured repeatedly
☐	☒	The statistical test(s) used AND whether they are one- or two-sided<br>*Only common tests should be described solely by name; describe more complex techniques in the Methods section.*
☐	☒	A description of all covariates tested
☒	☐	A description of any assumptions or corrections, such as tests of normality and adjustment for multiple comparisons
☐	☒	A full description of the statistical parameters including central tendency (e.g. means) or other basic estimates (e.g. regression coefficient) AND variation (e.g. standard deviation) or associated estimates of uncertainty (e.g. confidence intervals)
☐	☒	For null hypothesis testing, the test statistic (e.g. *F*, *t*, *r*) with confidence intervals, effect sizes, degrees of freedom and *P* value noted<br>*Give P values as exact values whenever suitable.*
☒	☐	For Bayesian analysis, information on the choice of priors and Markov chain Monte Carlo settings
☒	☐	For hierarchical and complex designs, identification of the appropriate level for tests and full reporting of outcomes
☒	☐	Estimates of effect sizes (e.g. Cohen's *d*, Pearson's *r*), indicating how they were calculated

Our web collection on statistics for biologists contains articles on many of the points above.

Software and code

Policy information about availability of computer code

Data collection	Illumina Miniseq control softwares were used to collect high throughput sequencing data.
Data analysis	Software used for data analysis:<br>R Studio 1.4.1717<br>FlowJo version 10.7.2<br>CRISPResso2 version 2.0.20b––https://github.com/pinellolab/CRISPResso2 and https://crispresso.pinellolab.partners.org/submission<br>InDelphi version 0.18.1- https://indelphi.giffordlab.mit.edu/

For manuscripts utilizing custom algorithms or software that are central to the research but not yet described in published literature, software must be made available to editors and reviewers. We strongly encourage code deposition in a community repository (e.g. GitHub). See the Nature Portfolio guidelines for submitting code & software for further information.

Data

Policy information about availability of data

All manuscripts must include a data availability statement. This statement should provide the following information, where applicable:
- Accession codes, unique identifiers, or web links for publicly available datasets
- A description of any restrictions on data availability
- For clinical datasets or third party data, please ensure that the statement adheres to our policy

High-throughput sequencing data have been deposited in the NCBI Sequencing Read Archive database under Accession Number PRJNA819982

Field-specific reporting

Please select the one below that is the best fit for your research. If you are not sure, read the appropriate sections before making your selection.

- [x] Life sciences
- [ ] Behavioural & social sciences
- [ ] Ecological, evolutionary & environmental sciences

For a reference copy of the document with all sections, see nature.com/documents/nr-reporting-summary-flat.pdf

Life sciences study design

All studies must disclose on these points even when the disclosure is negative.

Sample size	No statistical methods were used to predetermine sample size. Sample sizes were determined (n = 3) based on literature precedence for genome editing experiments (e.g. Rees et al., Nat Comms 2019, https://www.nature.com/articles/s41467-019-09983-4).
Data exclusions	No data was excluded.
Replication	All experiments were performed as at least three independent biological experiments, as specified for each experiment in the manuscript figure captions. All replications were successful.
Randomization	Mammalian cells used in this study were grown under identical conditions; no randomization was used.
Blinding	Mammalian cells used in this study were grown under identical conditions; no blinding was used.

Reporting for specific materials, systems and methods

We require information from authors about some types of materials, experimental systems and methods used in many studies. Here, indicate whether each material, system or method listed is relevant to your study. If you are not sure if a list item applies to your research, read the appropriate section before selecting a response.

Materials & experimental systems

n/a	Involved in the study	
x		Antibodies
	x	Eukaryotic cell lines
x		Palaeontology and archaeology
x		Animals and other organisms
x		Human research participants
x		Clinical data
x		Dual use research of concern

Methods

n/a	Involved in the study	
x		ChIP-seq
	x	Flow cytometry
x		MRI-based neuroimaging

Eukaryotic cell lines

Policy information about cell lines

Cell line source(s)	HEK293T, HeLa and K562 from ATCC.
Authentication	Authenticated by supplier by STR analysis.
Mycoplasma contamination	Cells were tested both by ATCC and our lab for mycoplasma contamination. Cells are regularly tested (every few months) to monitor mycoplasma contamination. All test were negative for contamination.
Commonly misidentified lines (See ICLAC register)	No commonly misidentified cell lines were used in this study.

Flow Cytometry

Plots

Confirm that:

- [x] The axis labels state the marker and fluorochrome used (e.g. CD4-FITC).
- [x] The axis scales are clearly visible. Include numbers along axes only for bottom left plot of group (a 'group' is an analysis of identical markers).
- [x] All plots are contour plots with outliers or pseudocolor plots.
- [x] A numerical value for number of cells or percentage (with statistics) is provided.

Methodology

Sample preparation

HEK293T cells were analyzed via flow cytometry to assess GFP knock-in efficiency fourteen days after transfection. Cells were washed with 250 µL phosphate buffered saline (PBS, Gibco #10010-023) in the plate and then detached from the plate with Accumax (Innovative-Cell Technology #AM-105) according to the manufacturer's instructions. After harvesting, cells were resuspended in 500 µL PBS. Samples were filtered into FACS tubes (Falcon, #352235) and kept on ice until analysis. HEK293T cell viability for off-target experiments was also analyzed via flow cytometry 72 hours after transfection. Cells were washed with 250 µL PBS on the plate and then detached from the plate with Accumax (Innovative-Cell Technology #AM-105) according to the manufacturer's instructions. After harvesting, cells were resuspended in propidium iodide staining buffer (PI, Invitrogen #1304MP) following the manufacturer's instructions. Samples were filtered into FACS tubes and kept on ice until analysis.

Isogenic cells for the zygosity experiment were generated using FACS. Cells were prepared for sorting as described above. Samples were gated against untransfected samples as described below. Single GFP positive cells (cells expressing Cas9) were sorted into 96 well plates 48 hours post transfection. Prior to sorting, wells were filled with 200 µL of 30% (V/V) FBS DMEM media and incubated at 37ºC. After sorting, plates were kept in the incubator for 3 weeks for clonal expansion, then harvested for NGS analysis.

All HeLa and K562 cell experiments required FACS (using GFP fluorescence) before NGS analysis. HeLa cells were prepared the same as the HEK293T cells described above. For K562 cells, cells were spun down at 300g for 5 minutes, the supernatant was decanted, and cells were washed with another 500 µL PBS. Following the second wash, the cell pellets were resuspended in 500 µL PBS and kept on ice until sorting. K562 cells were collected into RPMI 1640 supplemented with 20% (V/V) FBS, and HeLa cells were collected into DMEM supplemented with 20% (V/V) FBS. Both cell lines were then spun down, washed with 500 µL PBS, and then prepped for NGS.

Instrument

Biorad S3e cell sorter and BD AriaII cell sorter.

Software

FlowJo version 10.7.2

Cell population abundance

Example of cell population is showed on Figure 3. Knock in samples had 1-5% GFP positive population. K562 cells had around 5% and HeLa around 20% GFP positive cells.

Gating strategy

The instruments were calibrated and quality control checked before each flow cytometry or FACS experiment. Single color (e.g. pool of the transfected samples for each group) and no color (untransfected cells) control cell populations were used to set up gating. Single color (e.g. GFP positive cells for knock-in) had higher intensity than the untransfected cells for the corresponding channels (e.g. GFP channel for knock-in). We selected the (e.g. GFP) population based on untransfected cells. Gates were set up or checked with the untransfected and single color controls for each flow cytometry or FACS experiment. Example of the gates are shown in Supplementary Figure 14. Doublets were gated out using forward and side scattering width against area, and 20,000 events were analyzed.

- [x] Tick this box to confirm that a figure exemplifying the gating strategy is provided in the Supplementary Information.
